# LMAP_S: Lightweight Multigene Alignment and Phylogeny eStimation

**DOI:** 10.1186/s12859-019-3292-5

**Published:** 2019-12-30

**Authors:** Emanuel Maldonado, Agostinho Antunes

**Affiliations:** 1 CIIMAR/CIMAR – Interdisciplinary Centre of Marine and Environmental Research, University of Porto, Terminal de Cruzeiros do Porto de Leixões, Av. General Norton de Matos, s/n, 4450-208 Porto, Portugal; 2 Department of Biology, Faculty of Sciences, University of Porto, Rua do Campo Alegre, 4169-007 Porto, Portugal

**Keywords:** Multiple sequence alignment, Accuracy, Uncertainty, Character coding, Phylogeny, Consensus, Software package, High-throughput, Multigene, Multi-core

## Abstract

**Background:**

Recent advances in genome sequencing technologies and the cost drop in high-throughput sequencing continue to give rise to a deluge of data available for downstream analyses. Among others, evolutionary biologists often make use of genomic data to uncover phenotypic diversity and adaptive evolution in protein-coding genes. Therefore, multiple sequence alignments (MSA) and phylogenetic trees (PT) need to be estimated with optimal results. However, the preparation of an initial dataset of multiple sequence file(s) (MSF) and the steps involved can be challenging when considering extensive amount of data. Thus, it becomes necessary the development of a tool that removes the potential source of error and automates the time-consuming steps of a typical workflow with high-throughput and optimal MSA and PT estimations.

**Results:**

We introduce LMAP_S (Lightweight Multigene Alignment and Phylogeny eStimation), a user-friendly command-line and interactive package, designed to handle an improved alignment and phylogeny estimation workflow: MSF preparation, MSA estimation, outlier detection, refinement, consensus, phylogeny estimation, comparison and editing, among which file and directory organization, execution, manipulation of information are automated, with minimal manual user intervention. LMAP_S was developed for the workstation multi-core environment and provides a unique advantage for processing multiple datasets. Our software, proved to be efficient throughout the workflow, including, the (unlimited) handling of more than 20 datasets.

**Conclusions:**

We have developed a simple and versatile LMAP_S package enabling researchers to effectively estimate multiple datasets MSAs and PTs in a high-throughput fashion. LMAP_S integrates more than 25 software providing overall more than 65 algorithm choices distributed in five stages. At minimum, one FASTA file is required within a single input directory. To our knowledge, no other software combines MSA and phylogeny estimation with as many alternatives and provides means to find optimal MSAs and phylogenies. Moreover, we used a case study comparing methodologies that highlighted the usefulness of our software. LMAP_S has been developed as an *open-source* package, allowing its integration into more complex *open-source* bioinformatics pipelines. LMAP_S package is released under GPLv3 license and is freely available at https://lmap-s.sourceforge.io/.

## Background

Recent advances in genome sequencing technologies and the cost drop in high-throughput sequencing, allowed a new era of genome science, widening the amount of data available for downstream analyses [1, 2]. As the genomes become completely sequenced and assembled, they are subsequently released to public databases, such as Ensembl [3] and/or NCBI Genbank [4]. This allows other researchers to easily build datasets to their own object of study [5]. Evolutionary biologists often make use of such (nucleotide) data to uncover phenotypic diversity and adaptive evolution in protein-coding genes [6–10]. However, to perform such studies, multiple sequence alignments (MSA) and phylogenetic trees (PT) need to be estimated. In fact, the MSA is of central importance in molecular biology, many bioinformatics analyses and other areas of study, such as comparative sequence analyses, functional motif or domain characterization, prediction of secondary or tertiary structures, sequence-based structural alignment (e.g., [11, 12]), detection of key functional residues and homology searches [13–16]. With such importance, MSAs raise relevant questions concerning their accuracy [14, 15, 17, 18] or uncertainty [19–21], which can negatively influence downstream analyses, starting with phylogeny estimations [16, 18, 22, 23].

To date several software has been developed (with different objectives and approaches [15, 16, 24]) to improve MSA estimation (e.g., *MULTAN* [25], *MUSCLE* [26], *PRANK* [27], *MO-SAStrE* [28]) and refinement (e.g., *Gblocks* [29], *GUIDANCE* [14], *TrimAl* [30], *MaxAlign* [31]). Despite the efforts for reaching optimal solutions, uncertainty and confidence in the result persists, with co-optimal solutions not being the “true” alignment and/or the “true” alignment possibly being suboptimal [14].

Beyond adaptive evolution analyses, PTs are also of great importance for various biological research, for instance, the inference of trait evolution, protein structure and function [32] or in other phylogenomics areas, e.g. gene family evolution [9, 10]. Likewise, they suffer from identical uncertainty [13, 19, 33] issues (additionally aggravated by the MSA issues [13–17, 19, 20, 23, 34]). This has taken to the development of several improvements in algorithms and heuristics leading to alternatives, such as *PAUP* [35], *PHYLIP* [36], *PhyML* [37], *RaxML* [38], *FastTree* [39], or *MrBayes* [40].

In fact, the investment in improving and developing novel MSA and/or phylogeny estimators, has reached a level where it becomes difficult to the researcher to select the appropriate MSA [24] and/or phylogeny software. For the interested reader, during our literature survey, we have encountered more than 30 MSA software published solely respecting DNA multiple alignment (a few of them also enabling other data types); beyond other cases like amino acid and RNA, exceeding in total 80 software [24]. Many of them are presently unavailable or discontinued.

Considering the large amount of data (genomes) currently and in the future available, with the evolutionary biologist requiring the analyses of datasets with multiple genes (including cases of large gene families). Additionally, considering the preparation of an initial multiple sequence file(s) (MSF) dataset and the several steps involved to achieve the result of optimal MSAs and PTs, it becomes necessary the development of a tool that automates the time-consuming steps and accelerates estimations. For a single gene, the steps involved typically include, *(i)* the preparation of the initial protein-coding gene sequences MSF (e.g., *EASER* [5]), *(ii)* MSA estimation, *(iii)* MSA refinement, *(iv)* MSA substitution saturation detection (e.g., *DAMBE* [41, 42]), *(v)* detection of data fitting evolutionary model (e.g., *JModelTest* [43, 44], *MrAIC* [45]), *(vi)* phylogeny estimation (using the detected best model) and any *(vii)* phylogenetic tree posterior editing.

Several bioinformatics tools have been developed that consider MSA estimation and/or phylogeny estimation. They can be organized in two categories: *(i)* Command-line Interfaces (CLI), such as *M-coffee* [24], *SATé* [46], *POTION* [47], *ETE* [48] and, *(ii)* Graphical user interfaces (GUI), such as *DAMBE* [42], *StatAlign* [34], *Bosque* [49], *PALM* [50], *Seaview* [51], *Armadillo* [52]. Still, to our knowledge and from the available literature, none of them covers the aforementioned steps in an automated fashion, with the purpose of *(i)* enabling MSA and phylogeny high-throughput estimations, *(ii)* providing optimal estimation strategies, and *(iii)* including the additional characteristic of generating reproducible experiments [53, 54].

Here we present LMAP_S (Lightweight Multigene Alignment and Phylogeny eStimation), a high-throughput, versatile and user-friendly software package developed in Perl [55], built on top of our recent *LMAP* [7] package platform. LMAP_S was designed to handle in seven stages: *(i)* the input nucleotide (MSF) data pre-processing (NDP); *(ii)* the MSA estimation (AE); *(iii)* the MSA outlier detection (AOD); *(iv)* the MSA refinement and consensus (ARC); *(v)* the phylogeny estimation (PE); *(vi)* the phylogeny comparison and consensus (PCC) and *(vii)* phylogeny data post-processing (PDP). LMAP_S package consists of a single application, *lmap-s.pl*, which executes the aforementioned stages in a systematic fashion and depending on the user requirements. LMAP_S conveniently requires input nucleotide datasets, thus enabling (among others) further downstream evolutionary analyses [13]. With these objectives in mind, LMAP_S integrates various software covering all stages, except in *(i)* and *(vii)*.

To enable trial and testing, we adapt the example dataset from *LMAP* [7] to the current case, consisting of the mitochondrial DNA of 20 freshwater and terrestrial turtles and provide it in LMAP_S archive. Additionally, this is complemented with a case study on mitochondrial genes from a previously studied Cephalopoda dataset [56].

In the following sections, we present LMAP_S development and scheduling of tasks executions; integrated software in relation with stage organization and file identification; phylogeny estimation and the criteria for evolutionary model detection and alternative approach to typical substitution saturation detection; and lastly, the PCC method. Next, we present the functioning of LMAP_S, discuss integrated software options, the PCC method and potential future developments. Finally, we introduce (*i*) the example dataset used to perform the benchmarking tests; and (*ii*) the case study, explored to demonstrate the usefulness of LMAP_S.

### Implementation

#### LMAP_S development

LMAP_S package was implemented in Perl [55] and has been tested in Linux/UNIX. It consists of one command-line/interactive application, *lmap-s.pl*. Additionally, seven specific LMAP_S library modules (*MyUtil.pm*, *MyISWU.pm*, *MyNotify.pm*, *MyPhyloInfo.pm, MyPPMSF.pm, MyMMAP.pm* and *MyPhylo.pm*) were developed to support its functionality.

LMAP_S requires the Comprehensive Perl Archive Network (CPAN) [57] modules in four cases: *(i)* in *MyPhylo.pm*, for parsing and editing of Newick tree files (BioPerl [58] module); *(ii)* in *MyMMAP.pm,* for interactive monitoring of parallel executions (for which is required the UNIX *screen* [59] utility program); *(iii)* in *MyNotify.pm*, for email notifications (for which is required the UNIX *sendmail* [60] utility program); and *(iv)* in all, for handling files and directories.

The *MyMMAP.pm* module was adapted and improved from the *mmap.pl* application of *LMAP* [7] to allow the parallel execution of the diversity of software here integrated and to cope with the several stages of LMAP_S execution. Its functioning was largely maintained and is re-described in Additional file 1: Section 1. Other modules adapted from *LMAP* package are the *MyUtil.pm*, *MyPhylo.pm* and the *MyNotify.pm*.

LMAP_S includes an additional application, *RYcode.pl*, to enable the RY-coding of MSAs (see following section). Beyond being part of LMAP_S, it was also designed to enable independent operation from our package.

#### LMAP_S integrated software, stage organization and file identification

In this section, we list LMAP_S integrated software, to show how they are organized into stages and subsequently how in relation file identification was designed.

With exception of the first (NDP) and last (PDP) stages, Table 1, lists the integrated software for remaining stages.

**Table 1:** LMAP_S listing of integrated software (31) and related stages

LMAP_S Stage	Integrated Software	References	Algorithms Implemented
Stage 2 (AE)	Clustal Omega (v.1.2.1)	[61]	*Default*
	ClustalW (v.2.1)	[62]	*Default*
	Dialign-tx (v.1.0.2)	[63]	(3) Dialign-tx *Default*; Dialign-tx -D option; Dialign-tx -T option
	FSA (v.1.15.9)	[64]	(2) FSA *Default*; FSA with ‘nucprot’ option
	GramAlign (v.3.0)	[65]	*Default*
	Kalign (v.2.04)	[66]	*Default*
	MACSE (v.1.0.2)	[67]	(2) MACSE *Default*, MACSE with pseudogene alignment
	MAFFT (v.7.271)	[68]	(8) MAFFT *Default*, MAFFT with ‘auto’ option, MAFFT E-INS-I, MAFFT FFT-NS-1, MAFFT FFT-NS-2, MAFFT FFT-NS-I, MAFFT G-INS-I, MAFFT L-INS-I
	MUSCLE (v.3.8.31)	[26]	*Default*
	Opal (v.2.1.3)	[69]	*Default*
	Prank (v.150803)	[27]	(6) Prank *Default*, Prank +F option, Prank ‘once’ option, Prank *CODON*, Prank *CODON* + F option, Prank *CODON* ‘once’ option.
	ProbAlign (v.1.4)	[70]	*Default*
	ProbCons (v.1.12)	[71]	*Default*
	T-COFFEE (v.11.00.8cbe486)	[72]	(4) *Default* ‘PROBA_PAIR’, ‘T_COFFEE_MSA’, ‘KTUP_MSA’, ‘PLIB_MSA’
Stage 3 (AOD)	OD-Seq (v.1.0)	[73]	*Default*
	EvalMSA (v.1.0)	[74]	*Default*
Stage 4 (ARC)	Gblocks (v.0.91b)	[29, 75]	(2) *Default* DNA, *Default* CODON
	MaxAlign (v.1.1)	[31]	*Default*
	MergeAlign (n.f.)	[76]	*Default* (#)
	Noisy (v.1.5.12)	[77]	*Default*
	PSAR-Align (v.1.0)	[78]	*Default*
	TCS (T-COFFEE) (v.11.00.8cbe486)	[79]	(3) TCS, TCS_original, TCS_FM
	TrimAl (v.1.4)	[30]	(6) TrimAl *Default*, TrimAl ‘automated1’, TrimAl ‘gappyout’, TrimAl ‘strictplus’, TrimAl ‘strict’, TrimAl ‘compareset’ (#)
	WeaveAlign (v.1.2.1)	[20]	*Default* (#)
Stage 5 (PE)	IQ-TREE (v.1.6.2)	[80, 81]	(15) IQ-TREE DNA, IQ-TREE DNA (DEG), IQ-TREE DNA (RY), IQ-TREE CODON, IQ-TREE NT2AA. Each case is available for *Default* and *Standard* / *UFBoot* [81] Bootstraps
	MPBoot (v.1.1.0)	[82]	(2) MPBoot DNA. Each case is available for *Default* and “*UFBoot”* Bootstraps
	Ninja (v.1.2.2)	[83]	*Default*
	SMS (v.1.8.1)	[84]	(4) AIC + NNI, AIC + SPR, BIC + NNI, BIC + SPR
	Degen (v.1.4) (*)	[85, 86]	*Default*
	RYcode (v.1.0.0) (*)	This work	*Default*
Stage 6 (PCC)	CONSEL (v.1.20)	[87]	*Default (includes makermt, consel and catpv)*
	TreeCmp (v.1.1)	[88]	*Default*

**Legend**: (Number) – Algorithms Implemented column, where present, indicates the total number of algorithms implemented. (n.f.) – not found. (*) – Integrated as part of Stage 5 *IQ-TREE* algorithms DNA (DEG) and DNA (RY). (#) – Stage 4 consensus algorithms. DNA (nucleotide coding), DEG (degeneracy coding), RY (puRine and pirYmidine coding), NT2AA (translated – amino acid coding). AIC (Akaike Information Criterion) [89], BIC (Bayesian Information Criterion) [90], NNI (Nearest-Neighbor Interchange), SPR (Subtree Prunning and Regrafting). dN (non-synonymous distance), dS (synonymous distance). Listed software versions (see also Additional file 2: Figure S7) are only for reference of working cases and can be replaced by newer ones

This software is the result of criteria, whose main goal was to ensure they could be properly integrated and function correctly in the necessary conditions. Three examples of such “pipeline-friendly” criteria are: *(i)* command-line options enabling automation, *(ii)* facilitated accessibility to additional input dependencies, and *(iii)* program termination.

All the stages provide options that enable algorithm selection, except AOD and PCC (Additional file 2 and 3). The “*Default*” options besides being frequently preferred [15] were also designed to allow customization by the interested researcher.

File identification has been implemented to help the researcher to recall the algorithms that have manipulated the dataset genes [19] and to further allow any comparisons. This is done in a stage-by-stage fashion by using the algorithms identification (Additional file 3). Hence, the general format for MSA file identification is [GeneName]_[STAGE2AL]_[STAGE4AL].fas and for PT file is [GeneName]_[STAGE2AL]_[STAGE4AL]_[STAGE5AL]_[STAGE7ED].nwk (without brackets). For more information on these topics, please see the LMAP_S Manual.

#### LMAP_S phylogeny estimation and evolutionary model detection

Here we present how we integrate the data-fitting evolutionary model detection step, required prior to Maximum Likelihood (ML) PT estimation.

Evolutionary model selection involves testing all substitution models available (e.g., Table 1 in [43]) and select the best according to criteria, such as Akaike Information Criterion (AIC) [89] or Bayesian Information Criterion (BIC) [90]. This typically requires the researcher to run a priori software, such as *JModelTest* [43, 44] or *MrAIC* [45], which would add further complexity to LMAP_S workflow. However, with recent advances it becomes straightforward to have both evolutionary model detection and consecutive phylogeny estimation in the same software. For this reason, we have included *IQ-TREE* [80] and *SMS* [84].

#### LMAP_S phylogeny estimation and alternative to substitution saturation detection

Following the previous section, here we present the alternative solution for the substitution saturation detection, with its foundation, and the reasoning behind the presented solution.

Substitution saturation is a mutational process that (when present) negatively affects the information contained in molecular sequences of the MSA. This process affects the codon positions and takes to a decrease in phylogenetic information/signal. This phylogenetic signal is thus important for a reliable well-defined phylogeny estimation [41, 91].

Substitution saturation test [41] is a step typically performed to detect saturation in the MSA. This is done to ensure it contains sufficient phylogenetic information/signal before the phylogenetic tree estimation [23, 41, 91]. To perform the mutational saturation test, the *DAMBE* [42] software is available. However, its integration in LMAP_S bears a few difficulties, for instance, its incompatibility with Linux/UNIX systems. On the other hand, metrics for estimating phylogenetic signal are available and provide valid results [92]. Still, we have not found any suitable software.

To overcome this adversity, we have devised a methodology that gathers *(i)* the most relevant character coding (CC) methods [93] and *(ii)* phylogeny comparison methods. Together they help decide under different conditions which phylogeny provides a better resolution and is thus optimal.

Firstly, among the possible character coding methods, those frequently employed consist of plain nucleotide (DNA), 1st and 2nd codon positions only (DNA12), 3rd positions only (DNA3), puRine and pYrimidine coding (RY), degeneracy coding (DEG), codon (CDN) and amino acid (AA) [93]. According to Simmons [93] conclusions and for our methodology, the final selected CC methods are DNA, RY, DEG, AA and CDN, which we have implemented with *IQ-TREE* algorithms (Table 1). The specific CC methods DEG and RY are accomplished by combining *IQ-TREE* DNA data type with *Degen* [85, 86] and *RYcode* (Table 1 and Additional file 2: Figure S2). For these two cases, MSA coding is performed prior to *IQ-TREE* execution.

Secondly, we employ phylogeny comparison algorithms (Table 1). They are included to attempt to capture the phylogeny strategy that is consensually inferred to be the optimal among all selected CC cases (see following section). Henceforth, we refer to strategy, as the chain of algorithms applied to a specific gene, since AE Stage (see also section *LMAP_S Integrated software, stage organization and file identification*). This methodology was inspired and adapted to *IQ-TREE* [80] following the analyses performed by Simmons [93].

#### LMAP_S phylogeny comparison and consensus (PCC) method

Here we describe the implemented procedures devised to allow the comparison of several PTs (per gene) both topologically and statistically. Next, we describe how their combination is achieved to identify optimal consensus strategies (c.f. previous section; Additional file 4). This includes a total of six LMAP_S reports.

Phylogeny comparison is accomplished with two distinct approaches. One approach consists in statistical analyses, employing the site-wise log-likelihoods (SWLH) produced by several phylogeny estimators (e.g., *PAUP* [35], *PHYML* [37], *TREE-PUZZLE* [94]). Another approach, consists in the topological analyses, employing methods that target comparison of tree structure, nodes, branches and leafs (e.g., Robinson-Foulds (R-F) [95] and MatchingPair (MP) [88]). For the former case, we have integrated the *CONSEL* [87] package (which consists of three programs executed in the following order: *(1) makermt*, *(2) consel* and *(3) catpv*). For the latter we have integrated the *TreeCmp* [88] package. From both approaches three reports are generated (Additional file 5: Tables S3-S5), one from the statistical analyses and two from the topological analyses (including *TreeCmp* MP and R-F_C methods). To achieve these reports, LMAP_S processes data (SWLHs and PTs) generated by the different *IQ-TREE* algorithms (PE Stage; Table 1 and Additional file 4). The comparison of multiple SWLHs, requires their agglutination into a single file. Here, due to the discrepancies with resulting SWLHs, a heurist was implemented (Additional file 1: Section 2). This file is then served as input to *CONSEL* (i.e., to *makermt*). Likewise, *TreeCmp* input requires a single file containing all Newick formatted topologies. At this point, *TreeCmp* is ready for comparison. Hereafter, we describe how the results from both approaches (Additional file 1: Section 3) are combined to achieve the consensus.

An initial consensus report (Additional file 5: Table S6) is formed by using both *CONSEL* and *TreeCmp* MP results (Additional file 4). This is accomplished by locating in the MP report both *(i)* the top ranking statistical strategy and *(ii)* the corresponding optimal topological support. In detail, when such strategy match is found, the *TreeCmp* MP matrix row/column is searched for the best topological result (where the MP score is zero). When both criteria are met, the corresponding strategy is doubly marked, otherwise only once. LMAP_S further summarizes these results by successively deriving two additional reports. The fifth report is a condensed matrix discarding the unmarked strategies (Additional file 5: Table S7). Whereas, the last report, shows the total topological score (TTS) associated with each consensus strategy (Additional file 5: Table S8). To calculate the TTS of the consensus strategy, LMAP_S proceeds by counting all related zero values from the MP scores. Hence, the optimal consensus strategy (i.e., optimal underlying chain of algorithms), is one with the maximum TTS. For more details, please see the LMAP_S Manual.

## Results

**Fig. 1:**
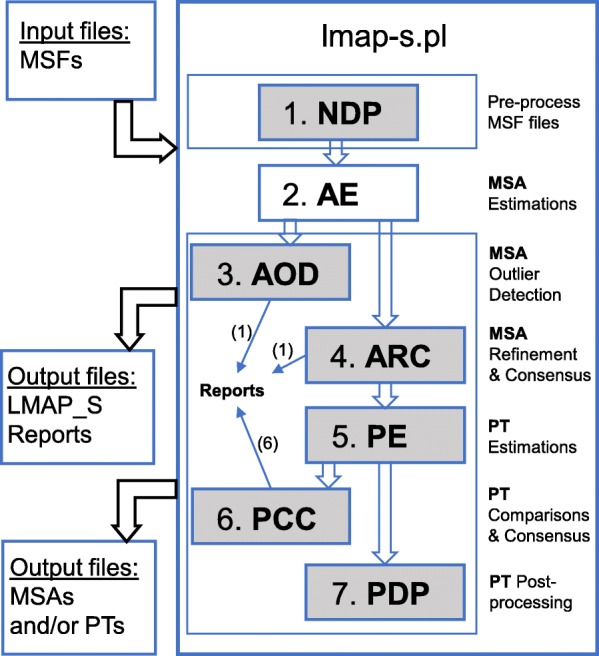
LMAP_S workflow. Flowchart exhibiting the *lmap-s.pl* workflow where stages are organized in a sequential fashion. The omission or inclusion of certain Stages helps devise specific workflows based on researcher requirements. Gray boxes reflect optional stages. Stages 3 (AOD) and 6 (PCC) produce reports only, seven in total. Stage 4 (ARC) additionally produces one report. NDP – Nucleotide Data Pre-processing, AE – MSA Estimation, AOD – MSA Outlier Detection, ARC – MSA Refinement and Consensus, PE – PT Estimation, PCC – PT Comparison and Consensus, PDP – PT Data Post-processing

The LMAP_S package consists of a single main application *lmap-s.pl* that executes the workflow comprising seven stages (Fig. 1). Only AE Stage is mandatory and thus LMAP_S gives the possibility to apply any of the remaining stages, provided the data dependencies are satisfied. Hereafter, LMAP_S functionalities are described in a stage-by-stage fashion.

### Stage 1 – NDP

This stage provides two modes of functioning, *(i)* a default mode and *(ii)* data treatment mode. In the default mode, it is responsible for the creation of the directory structure and placement of MSFs (expected in FASTA format). In the data treatment mode, beyond the default mode operations, the extra functionalities are available with additional arguments (Additional file 2: Figures S1 and S6). Possible data treatments are included for not-ready MSFs and ready MSFs. We consider the not-ready datasets, as the MSF(s) not expected to be grouped in files by gene homology.

### Stage 2 – AE

With all the MSFs ready and organized in the directory structure, this stage enables the alignment of every gene by all the algorithms here selected, thus estimating a different MSA version for each gene MSF. This requires the selection among 32 MSA algorithms (Table 1, Additional file 2: Figure S3 and Additional file 3). After completion, an additional procedure ensures that all MSAs have the same taxa order for the following stages.

### Stage 3 – AOD

Here, LMAP_S enables the identification and possible removal of divergent sequences in MSAs from the AE Stage (Table 1 and Additional file 3). It enables the generation of the outlier report (Additional file 5: Table S1), containing the results from two software, *OD-Seq* [73] and *EvalMSA* [74], gathered for further result complementarity [74]. This stage report or results will not interfere with further stages (Fig. 1).

### Stage 4 – ARC

This stage targets the employment of several algorithms for MSA refinement and consensus (Table 1, Additional file 2: Figure S4 and Additional file 3). The refinement algorithms (13 in total) have the purpose to improve each MSA phylogenetic signal by either removing, masking or duplicating MSA eventual ambiguous regions [32]. Whereas, the consensus algorithms (3 in total) enable the combination of several MSAs from different sources. In this case, it is possible to find the best result (*TrimAl* “compareset” [30]) or a result that gathers the best characteristics among all MSAs [20, 76]. To enable the quick identification of which original MSA was selected for the *TrimAl* “compareset” option, an additional CSV report is produced (Additional file 5: Table S2).

### Stage 5 – PE

Here, a variety of algorithms can be selected among 22 possibilities (Table 1, Additional file 2: Figure S5 and Additional file 3) for the estimation of phylogenies.

This stage currently provides two ML, one Maximum Parsimony (MP), and one Neighbor-Joining (NJ) approaches. The NJ is available with *Ninja* [83] software, the MP with *MPBoot* [82], and ML with *SMS* [84] and *IQ-TREE* [80]. Any of the phylogeny estimation algorithms can be used together without interfering with each other or with other stages.

### Stage 6 – PCC

This stage enables the comparison of phylogenies estimated by (two or more) *IQ-TREE* algorithms and of optimal consensus strategies (Additional file 4 and Additional file 5: Tables S3-S8). For more details, please see Implementation section *LMAP_S Phylogeny comparison and consensus (PCC) method*.

### Stage 7 – PDP

The last stage applies to the phylogenies resulting from the PE Stage. Similarly, to the NDP Stage data treatment mode, this allows the application of three phylogeny-editing options (Additional file 2: Figure S1). These enable preparations for further downstream analyses.

**Fig. 2:**
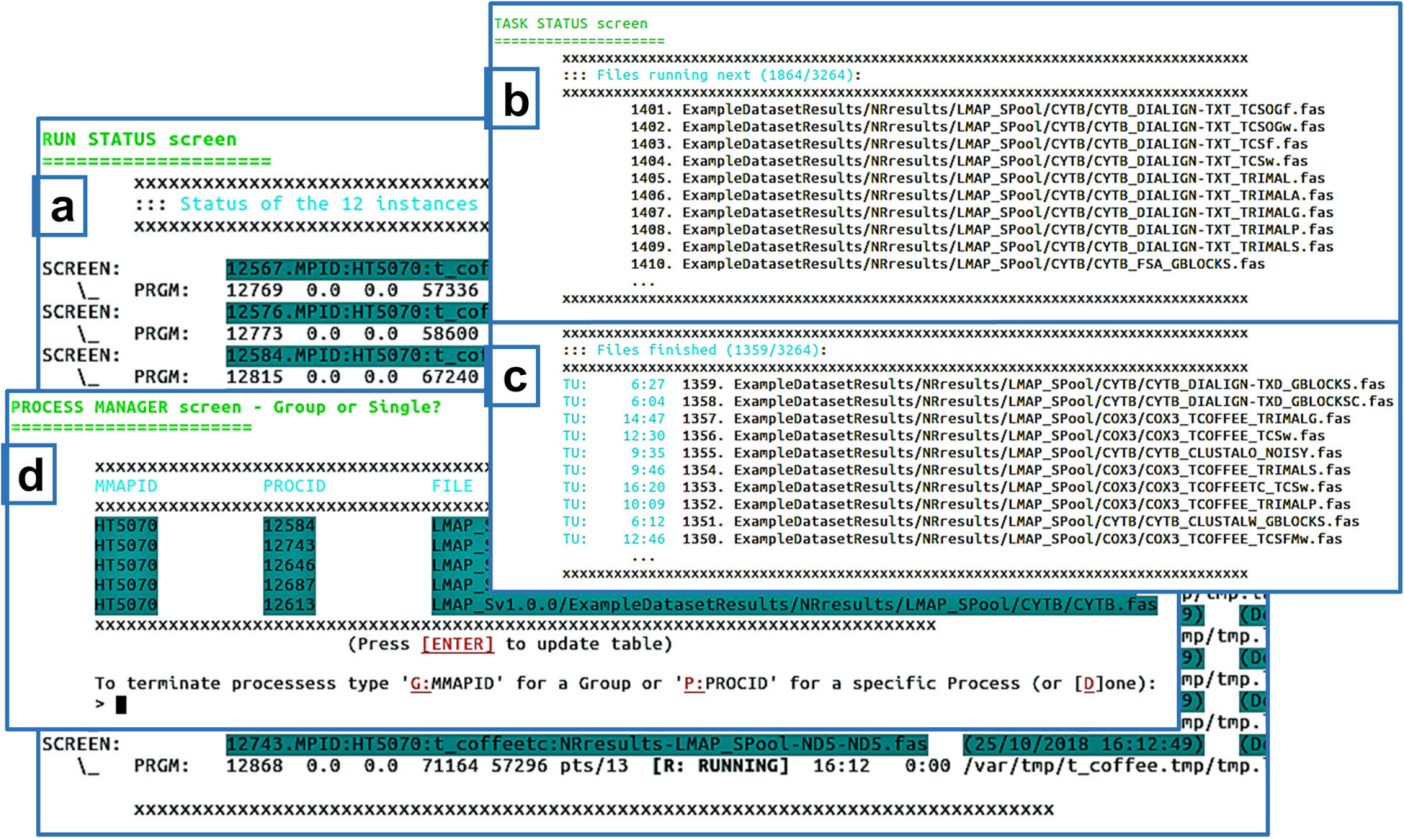
LMAP_S interactive functioning. **a** default or main “Run Status” screen presenting the currently running tasks; by pressing “2”, shows the “Task Status” screen, showing (**b**) the tasks that will be running next (first ten) and (**c**) the tasks currently finished (last ten) (press “1” to go back to (**a**)); (**d**) when interrupting the execution of *lmap-s.pl* (by typing “Ctrl-c” or “Ctrl-\”), beyond the choice of quitting, the user has also the choice to proceed to the built-in process manager here presented, allowing the termination of specific tasks. In this case, it is possible to terminate a group of tasks by typing “G:MMAPID” or a single task “P:PROCID”. The identifiers for MMAPID and PROCID are shown in the table, in the respective columns

LMAP_S described stages are subject to monitoring (Fig. 2) of integrated software executions until *lmap-s.pl* terminates. This is similar to *mmap.pl* application from *LMAP* package (see also Fig. 3 in [7]).

## Discussion

### LMAP_S implementation options and general remarks

The integrated software (Table 1) is the result of aforementioned criteria. Even though, we have not integrated a few relevant software in ARC and PE Stages. In the ARC Stage, the most striking is *GUIDANCE* [14] a widely-used software in which we have found a significant limitation. This software only works with MSAs from *PRANK* [27], *CLUSTALW* [62] and *MAFFT* [68]. Hence, its integration could limit LMAP_S functionality. In terms of phylogeny estimation, we have not integrated software such as *RAxML* [38], *FastTree* [39] or *MrBayes* [40]. We recognize their relevance and wide spread utilization, however, including them would go against the design established (see also section *LMAP_S Phylogeny estimation and evolutionary model detection*). Here, we require to minimize the number of stages in the workflow, hereby reducing workflow complexity. This can only be accomplished by *SMS* and *IQ-TREE* software, which incorporate the automated selection of the evolutionary best-fit models. Otherwise, if we were to integrate this software we would have to additionally integrate, for instance, *JModelTest* [44] and/or *MrAIC* [45]. Thus, another stage would be necessary previous to PE Stage. Additionally, both *RAxML* and *FastTree* provide limited selection of nucleotide models, which does not enable a well-supported justification for model selection [93]. Although *RAxML* supplies several alternatives, all are based on the GTR model. We understand that GTR is the most complex and successful model to date being selected for the most cases [96]. However, the analysis may become limited, if a different model is found as best fit. *FastTree*, additionally provides Jukes-Cantor nucleotide model [39]. Even though, both solutions are quite limited. On the other hand, *MrBayes* provides several models that can be employed. However, the complexity of automatically managing the commands that need to be specified/provided depends on each researcher and dataset, making it very hard to integrate.

Regarding the PCC Stage method, this software would have to provide SWLH data. *RAxML* is compatible (but does not support codon analysis [93]); the authors of *FastTree* provide an additional Perl script to convert into PAUP format (but still does not support DEG and RY-coding [39]), and *MrBayes* does not provide such information. However, as described by the authors of CONSEL, it only works with the matrices produced with ML methods [87]. Nevertheless, they could still be useful and attractive alternatives to complement the existing ones when only applied for the phylogeny estimations (PE Stage). Thus, considering the CC options and straightforward compatibility with *CONSEL* [87], *IQ-TREE* is the only ML integrated software that makes the PCC method possible (see also section *LMAP_S Phylogeny estimation and evolutionary model detection*).

**Fig. 3:**
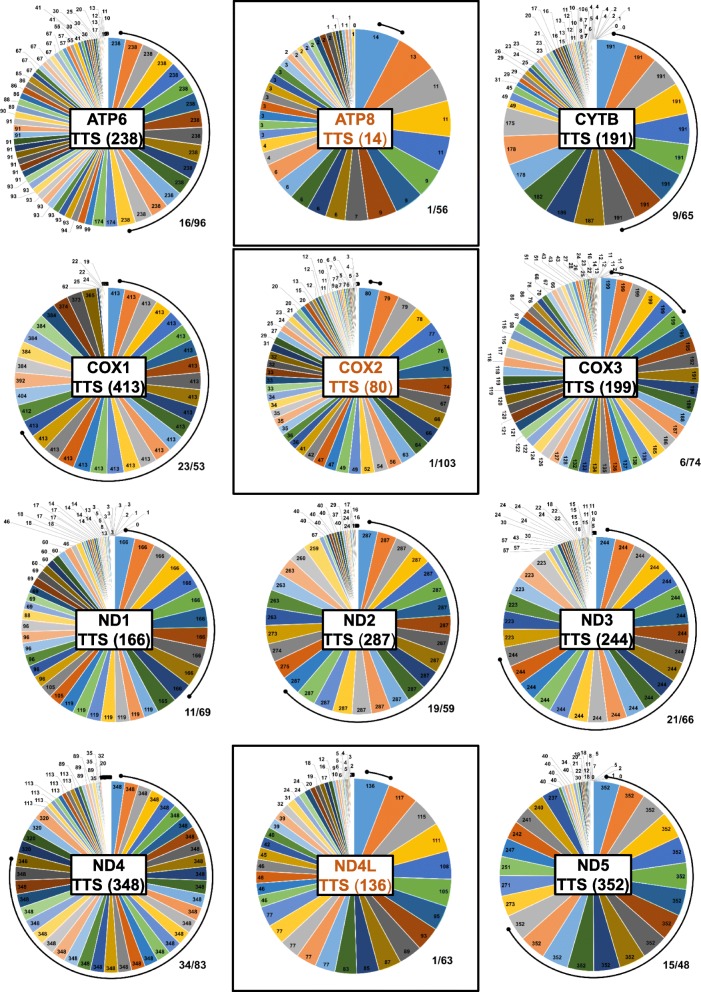
Pie charts exhibiting the optimal consensus strategies (with highest TTS). Illustration of the optimal results for the provided dataset derived from LMAP_S consensus histogram report (Additional file 5: Table S8). Each pie chart presents the results for each gene showing at the center the highest TTS value in parenthesis. The arc lines surrounding each pie chart highlights the amount of optimal consensus strategies. The fraction at the bottom right corner of each chart shows the number of consensus strategies with equal highest TTS over the total number of strategies. The three squared cases (genes ATP8, COX2 and ND4L) are the only ones showing a unique consensus strategy with highest TTS. These optimal strategies are “ATP8_MAFFTF2_TRIMALS_DNA_UB”, “COX2_PRANKCDF_MAXALIGN_DNA_UB” and “ND4L_MAFFTEI_MAXALIGN_DNA_UB”, from where it is clearly visible the different optimal algorithms (from AE and ARC Stages). Notably the optimal CC option (DNA) was the same in all three. For the remaining cases, it is possible to take any of the consensus strategies as optimal as long as they have the highest TTS

This method has been implemented to enable inference of reliable and well-defined phylogeny estimations. In the statistical approach, this is ensured by compiling SWLHs for the same *(i)* gene, *(ii)* CC method and *(iii)* possible refinement algorithms. Otherwise, collecting different CC methods or refinement algorithms SWLHs, would deteriorate the conditions for same site-wise lengths. In the topological approach, it is ensured by gathering the several topologies for the same gene. Finally, for each gene, the top statistical results are mapped into the wider range of topological results. Beyond being an alternative to substitution saturation detection (see section *LMAP_S Phylogeny estimation and alternative to substitution saturation detection*), this procedure additionally uncovers optimal consensus strategies supported by both the statistical and topological agreement (Additional file 5: Tables S6-S8). Specifically, a PT with highest TTS score has an optimal phylogenetic signal, resolution and underlying strategy. Thus, this method hereby addresses a very important topic, not always undertaken by researchers. Its importance stems from the fact that researchers frequently opt for a widely used MSA software or that is found as being better [18]. In fact, the same software is usually applied to all genes (or gene family), thus neglecting the possibility that other software might produce better results with specific genes [18, 24, 97]. Through the PCC method, example dataset and case study, LMAP_S shows that there may be different or better MSA choices for different or specific genes (Fig. 3, Additional file 5: Table S8 and Additional file 6). In fact, the works [18, 24, 97] support the notion that neither the dataset nor the algorithms define the optimal MSA strategy. Furthermore, some authors regard the impact of the phylogeny estimation as an alternative way to evaluating MSA methods [13, 98]. This perspective provided by our software is also partly supported by Beiko et al. [97] in the case of the “shotgun” MSA approach.

Our software, makes possible to execute many different analyses and with different extensions. For instance, at minimum it is possible to estimate MSAs (with possible NDP Stage data treatments), and to a maximum extent, it is possible to have phylogenies ready for any downstream analyses (e.g., adaptive evolution). With the several integrated software and options, we foresee LMAP_S can have potential application in several scenarios. Among which we mention, *(i)* preliminary data study, *(ii)* finalized data for downstream analyses, *(iii)* benchmarking purposes and algorithms comparison, *(iv)* study of optimal strategies for each gene (MSA and PT estimations), *(v)* large-scale gene and (phylo) genomic analyses, and also to *(vi)* serve the input for *LMAP* [7] and/or *IMPACT_S* [6] packages.

Comparatively, LMAP_S extends the mentioned software in CLI and GUI categories in several ways e.g., high-throughput, MSA refinement, phylogeny comparisons. We found that *Bosque* [49] and *PALM* [50], present workflows most similar to our program. Contrarily to LMAP_S, they include less algorithm choices, provide client-server functioning and GUI interfaces, which although being possibly more user-friendly, always depend on external resources availability and may disrupt pipeline integration. Compared to other methods, LMAP_S does not intend to provide consensus PTs or MSAs for each gene. Instead, it is intended to provide high-throughput estimations and by the PCC method infer optimal phylogeny estimation strategies (Fig. 3 and Additional file 6), whereby the underlying chain of algorithms and methods reflected in the phylogenies are also optimal.

Presently, LMAP_S has been developed to gather the most software alternatives around nucleotide data type necessary for evolutionary analyses [13]. LMAP_S does not provide options for the concatenation of genes enabling multi-gene inferences. Still, it can help to estimate the necessary MSAs. We understand the relevance of such process, which we plan to implement soon. Furthermore, we recognize that LMAP_S may lack a stage (close to ARC Stage) where software can be employed to determine highest scoring MSAs for the next stages [17, 99], but it is discussed that the highest scoring MSAs are not necessarily the “true” MSA [14, 16–18]. Anyhow, we found that the software available (e.g., *FASTSP* [33]) often depended on a reference MSA (exception made for *MUMSA* [17]). To our understanding, the reference MSA is considered as the “true” MSA [13, 99], which for reasons mentioned before, becomes a contradictory possibility. This required feature poses several problems, for instance, when the data at hand (e.g., from newly assembled genomes) does not “readily” enable a priori reference MSA (usually available from the benchmark databases [14, 99, 100]). Hence, how can one determine it (or from a set of alternate nucleotide alignments of the same sequences)?

Additionally, LMAP_S could benefit from the integration of additional algorithms, for instance, in MSA masking (e.g., *ZORRO* [21], *SR* [23]) and other phylogeny estimation tools (e.g., *MrBayes* [40]). LMAP_S is not applicable in Windows OS due to its main dependency on the *screen* [59] utility program.

### Example dataset and benchmarking

An example dataset is provided in LMAP_S archive to help users explore and experience the workflow of the package. Except for the TMConc2 concatenated MSA, here we reuse the dataset explored in *LMAP* [7]. The folder (“ExampleDataset”) contains two directories, one for ready MSFs and the other for not-ready MSFs. The “Ready” folder contains the sequences organized by gene and the “NotReady” folder contains the sequences organized by genomes as downloaded from NCBI. In both cases, the sequences contain stop codons. To demonstrate the performance of LMAP_S, full command-lines are provided in LMAP_S archive in the “lmap-s.command” file, from where we have executed the “NotReady” one. Its output originated 3264 MSA and 30,392 PT files that took 4 days, 2 h, 45 min and 38 s to complete (5925 min and 38 s). This was measured in the UNIX *time* [101] utility program, by using a single workstation configured with 64GB of RAM and two Intel Xeon E5-2683v4 processors, which together yield a total of 64 hyper-threading cores. In contrast, using a single core, the same instances would take 14,628,152 s (more than five months). To summarize, our package does not interfere in the execution time required by each software, but instead mitigates how much the researcher spends overseeing each step of the workflow, from the moment the input files are ready to be analyzed, which may be none or minimal.

### Case study with Cephalopoda mitochondrial genes

To provide further insight on the usefulness of our software, we have employed a previously published dataset of 13 Cephalopoda mitochondrial genes [56]. As described, all the alignments were performed with *MUSCLE* [26]. Improvement over phylogenetic signal and resolution, considered the concatenation and RY-coding (3rd codon position). By employing LMAP_S we test two possible outcomes: *(i)* if the same strategy (*MUSCLE* and RY-coding) is inferred for all genes and *(ii)* if the optimal consensus strategies convey topology improvements when compared to the concatenated ML topology from the study.

The applied methods, results and discussion are presented in Additional file 6.

In conclusion, the application of a software with similar characteristics as LMAP_S in this study, would have been highly beneficial. With the differences found among strategies, these results support the application of a more precise chain of algorithms for each gene. Additionally, the fact that the LMAP_S topologies show better average scores, confirms that employing our software can provide more reliable phylogeny estimations and avoid performing gene concatenation and related analyses.

With this and previous example dataset, we show compelling results demonstrating that different strategies should be applied for different genes and that multi-gene concatenation methods are not the most powerful solution [102].

## Conclusions

We have developed a simple, versatile and highly customizable package named, Lightweight Multigene/Multi-core Alignment and Phylogeny eStimation (LMAP_S), that readily enables the application of several MSA (33) and PT (22) estimation algorithms. Beyond the central stages, it also enables MSF editing (NDP), AOD (2), and ARC (16) algorithms. With two algorithms, it enables phylogeny statistical and topological comparison and the combination of both to reach consensus in optimal phylogeny and consequently in the underlying MSA algorithms applied from the beginning. Finally, resulting phylogenies can be automatically edited for further downstream analyses. To our knowledge, no other software combines MSA and phylogeny estimation with as many alternatives and provides means to find optimal MSAs and phylogenies. Additionally, we have supplied evidence that LMAP_S is well-supported and useful in methodologies of alignments and phylogenies estimations.

At minimum, one MSF is required with the gene sequences to be analyzed within a single input directory. From this moment, LMAP_S automatically creates, organizes, executes, manipulates and extracts the necessary information from the integrated algorithms results to provide additional information and high-throughput estimations. Furthermore, LMAP_S enables at all times, monitoring and control of software and tasks, and email notification when the job is done. LMAP_S has been developed as an *open-source* command-line and interactive package, allowing its integration into more complex *open-source* bioinformatics pipelines.

### Availability and requirements

Project Name: LMAP_S.

Project Home Page: https://lmap-s.sourceforge.io/

Operating System: Linux/UNIX.

Programming Language: Perl.

Other Requirements: integrated software from Table 1, CPAN modules (IO::All, Email::MIME, Email::Sender, Sys::Info, Term::Readkey, Thread::Semaphore, Bio::TreeIO, File::Copy, File::Copy::Recursive), *screen* and *sendmail* UNIX command-line utilities.

License: GNU General Public License, version 3.0 (GPLv3).

Any restrictions to use by non-academics: no restrictions except the ones stated in GPLv3.

### Installation

The LMAP_S package provides two additional applications to facilitate LMAP_S functionality and installation: *(i)* the *install.pl* (requires *sudo* command) to enable the installation of LMAP_S dependencies from Linux repositories, such as CPAN modules, integrated software (Table 1) and UNIX utilities and *(ii)* the *configure.pl* to enable the configuration of LMAP_S package (*lmap-s.pl* and LMAP_S library). A manual with detailed instructions is included in the archive to allow LMAP_S user-friendly installation and application.

## Supplementary information

**Additional file 1.** Additional implementation and algorithms. (Section 1): LMAP_S Scheduling of tasks executions. (Section 2): Description and reasoning of SWLHs heuristic involved in the PCC method. (Section 3): Description of LMAP_S statistical and topological reports involved in the PCC method.

**Additional file 2. **Figures exhibiting LMAP_S applications options and stage arguments. (Figure S1): command-line options for *lmap-s.pl* application. (Figure S2): command-line options for *RYcode.pl* application. (Figure S3): *lmap-s.pl* arguments for algorithm selection in Stage 2 (AE). (Figure S4): *lmap-s.pl* arguments for algorithm selection in Stage 4 (ARC). (Figure S5): *lmap-s.pl* arguments for algorithm selection in Stage 5 (PE). (Figure S6): *lmap-s.pl* arguments for translation table selection. (Figure S7): *lmap-s.pl* display of available integrated software.

**Additional file 3.** Table extending Table 1 information. Shows absolute identification assigned to algorithms and respective code abbreviation, which enables their selection into LMAP_S stages.

**Additional file 4.** Flowchart illustrating the PCC method. Shows the several steps of the method starting with the PE Stage data until final consensus reports.

**Additional file 5: **Resulting CSV reports compiled from LMAP_S execution of the included example dataset. (Table S1): Stage 3 (AOD) outlier detection report. (Table S2): Stage 4 (ARC) *TrimAl* “compareset” report. (Table S3): Stage 6 (PCC) *CONSEL* report. (Table S4): Stage 6 (PCC) *TreeCmp* MP reports. (Table S5): Stage 6 (PCC) *TreeCmp* R-F_C reports. (Table S6): Stage 6 (PCC) consensus reports. (Table S7): Stage 6 (PCC) consensus brief reports. (Table S8): Stage 6 (PCC) consensus histogram reports.

**Additional file 6. **LMAP_S case study analyses of the Cephalopoda mitochondrial genes. (File 1): Description of experiments, results and discussion. (File 2): LMAP_S and TreeCmp command-lines with additional benchmarking. (File 3): Tables with LMAP_S consensus histogram reports from *CephaResults*. (File 4): Tables with LMAP_S consensus histogram reports from *CephaResultsARC*. (File 5): Figures showing side-by-side consensus strategies charts comparisons. (File 6): Tables with results of the topological comparisons. (File 7): Final and original LMAP_S results (PTs and Reports). (File 8): Bash scripts used to generate the TreeCmp input files.

## Data Availability

All additional files that support the findings of the current study are available as a collection in the figshare repository, 10.6084/m9.figshare.c.4743515.v2 [103]. Others are included in the LMAP_S software archive, which is available to download from the project home page. The archive version here revised (LMAP_S version 1.0.0) is also available from the project home page or by request.

## Abbreviations

AE: MSA Estimation (stage 2)
AIC: Akaike Information Criterion
AOD: MSA Outlier Detection (stage 3)
ARC: MSA Refinement and Consensus (stage 4)
BIC: Bayesian Information Criterion
CC: Character Coding
CLI: Command-Line Interface
CPAN: Comprehensive Perl Archive Network
CSV: Comma-Separated Values
DAMBE: Data Analysis in Molecular Biology and Evolution
EASER: Ensembl Easy Sequence Retriever
ETE: Environment for Tree Exploration
FSA: Fast Statistical Alignment
GUI: Graphical User Interface
GUIDANCE: GUIDe tree–based AligNment ConfidencE
IMPACT_S: Integrated Multiprogram Platform to Analyze and Combine Tests of Selection
LMAP: Lightweight Multigene Analyses in PAML
LMAP_S: Lightweight Multigene Alignment and Phylogeny eStimation
MACSE: Multiple Alignment of Coding SEquences
MAFFT: Multiple Alignment using Fast Fourier Transform
ML: Maximum Likelihood
MO-SAStrE: Multiobjective Optimizer for Sequence Alignments based on Structural Evaluations
MP (*TreeCmp*): MatchingPair
MP: Maximum Parsimony
MSA: Multiple Sequence Alignment
MSF: Multiple Sequence File
MUSCLE: MUltiple Sequence Comparison by Log-Expectation
NCBI: National Center for Biotechnology Information
NDP: Nucleotide Data Pre-processing (stage 1)
NJ: Neighbor-Joining
NNI: Nearest-Neighbor Interchange
PALM: Phylogenetic reconstruction by Automatic Likelihood Model selector
PAML: Phylogenetic Analysis by Maximum Likelihood
PAUP: Phylogenetic Analysis Using Parsimony
PCC: PT Comparison and Consensus (stage 6)
PDP: PT Data Post-processing (stage 7)
PE: PT Estimation (stage 5)
PHYLIP: PHYLogenetic Inference Package
POTION: POsitive selecTION
PSAR: Probabilistic Sampling-based Alignment Reliability
PT: Phylogenetic Tree
R-F_C: Robinson-Foulds_Cluster
SATé: Simultaneous Alignment and Tree estimation
SMS: Smart Model Selection
SPR: Subtree Prunning and Regrafting
SR: Signal Refinement
SWLH: Site-Wise Log-Likelihood
T-COFFEE: Tree-based Consistency Objective Function For alignmEnt Evaluation
TCS: Transitive Consistency Score
TTS: Total Topological Score
