# Supplementary material for: LMAP_S: Lightweight Multigene Alignment and Phylogeny eStimation
Source: BMC Bioinformatics. 2019 Dec 30;20:739. doi: 10.1186/s12859-019-3292-5 (PMC6937843; doi:10.1186/s12859-019-3292-5)
Supplement: Supplementary file 1 — Additional file 1. Additional implementation and algorithms. (Section 1): LMAP_S Scheduling of tasks executions. (Section 2): Description and reasoning of SWLHs heuristic involved in the PCC method. (Section 3): Description of LMAP_S statistical and topological reports involved in the PCC method. [file 12859_2019_3292_MOESM1_ESM.docx]

**Section 1: LMAP_S Scheduling of tasks executions (per stage)**

In this section, we describe how the *lmap-s.pl* application (through *MyMMAP.pm*) was designed to cope with several executions and its relation with the workstation CPU capacity.

At the various stages, the input files (MSFs, MSAs, etc) are ready for execution in subfolders within a base folder, which we refer to henceforth as directory structure. *LMAP_S* proceeds by executing all tasks one stage at a time, hence for instance, it will execute all MSA selected software in AE Stage and after it is finished, it will proceed to the next stage, and so on. The following applies to every stage of LMAP_S with integrated software.

LMAP_S allows the user to run as many tasks as desired. Because the total number of tasks can be very large, most probably surpassing the total number of CPU cores, the application provides an option to define the maximum tasks to be run. This will define the maximum number of cores utilized (one task per core). When used, a value for this option must be defined, or otherwise it becomes automatically estimated. In this case, the application quantifies an approximate number of available CPU cores, which in consequence defines the maximum number of tasks to be run. This is achieved by calculating the difference of total number of cores to the overall CPU load. Under these circumstances, it is wise to quantify the available CPUs by the application, since it maximizes the performance of the whole scheduling.

It is noteworthy, that the greater the number of CPU cores available, the faster the execution of the *lmap-s.pl* application will be. Nevertheless, this is highly dependent on the user’s workstation configuration (CPU, memory, etc.). Please see the *Example Dataset and Benchmarking* section for more information.

**Section 2: Description and reasoning of SWLHs heuristic involved in the PCC method.**

LMAP_S ensures that *CONSEL* functions correctly (i.e., all gathered strategies have same length) by selecting the SWLHs according to *(i)* same gene *(ii)* the same CC method and if ARC Stage was executed, *(iii)* to the same refinement/consensus algorithm. Even though, there may be SWLHs that result with different site lengths due to e.g., different gap insertions. Hence, to maximize the usefulness of our procedure we have implemented an intermediate heuristic. It consists in *(i)* distributing the SWLHs by their site-wise lengths in bins created for every novel site-wise length; and by the end, *(ii)* select the bin with the maximum number of items, which respect a single specific length. For instance, while analyzing the set of SWLHs, one may have an item with length of 348 sites, hereby creating a new bin for this length with its identity here inserted. This is performed whenever a new SWLH appears with a different length, otherwise, it will be added to the existing bin. By the time all are processed, a bin with the maximum amount of SWLHs is selected. Finally, all are joined and posteriorly compared with *CONSEL* package.

**Section 3: Description of LMAP_S statistical and topological reports involved in the PCC method.**

With the results ready from both statistical and topological approaches, LMAP_S generates the corresponding reports. In either approach, the reports have their SWLHs and topologies identified, i.e. all items are prepared in advance by LMAP_S with corresponding strategy/file identification. This will be useful for the final consensus processes and devised reports.

According to *CONSEL’s* *catpv* program, the statistical report is formed by the selection of the top ranking strategies (Additional file 5: Table S3) and includes information for every gene. On the other hand, the *TreeCmp* MP and R-F_C reports are formed independently for every gene and after the matrix comparison of *TreeCmp*, which enables all-to-all comparison without repetitions (Additional file 5: Tables S4-S5). Here, due to the *TreeCmp* MP reported improvements, this method report has been preferred over R-F_C report in further processes.
