# Supplementary material for: LMAP_S: Lightweight Multigene Alignment and Phylogeny eStimation
Source: BMC Bioinformatics. 2019 Dec 30;20:739. doi: 10.1186/s12859-019-3292-5 (PMC6937843; doi:10.1186/s12859-019-3292-5)
Supplement: Supplementary file 2 — Additional file 2. Figures exhibiting LMAP_S applications options and stage arguments. (Figure S1): command-line options for lmap-s.pl application. (Figure S2): command-line options for RYcode.pl application. (Figure S3): lmap-s.pl arguments for algorithm selection in Stage 2 (AE). (Figure S4): lmap-s.pl arguments for algorithm selection in Stage 4 (ARC). (Figure S5): lmap-s.pl arguments for algorithm selection in Stage 5 (PE). (Figure S6): lmap-s.pl arguments for translation table selection. (Figure S7): lmap-s.pl display of available integrated software. [file 12859_2019_3292_MOESM2_ESM.pdf]

```
NAME:

  lmap-s.pl - Lightweight Multigene Alignment and Phylogeny eStimation (LMAP_S).

SYNOPSIS:

  lmap-s.pl -A [MSFdir] {-p [p1,...,px]} -a [a1,...,ax] {-b} {-c [c1,...,cx]} {-m [option]} {-t [t1,...,tx]} {-s}
          {-q [q1,...,qx]} {-g [g1,...,gx]} -i [ttcode] {-n [nCPUs]} -d [projdir] {-j [projname]} {-e {email}} {-l}

DESCRIPTION:
  |  ----------------------------------------------------------------------------
  | Software package to estimate nucleotide alignments and corresponding phylogenies at large-scale with support for optimal results.
  | It incorporates several algorithm alternatives, which not only provide a wider set of choices, but also enable various
  | comparisons. It enables alignment outlier detection, alignment refinement and consensus as well as phylogenetic tree
  | comparisons and editing, with a diversity of methods and algorithms systematically applied to the same gene(s).

OPTIONS:
  |  ----------------------------------------------------------------------------
  | -A [MSFdir]     Input directory containing all the nucleotide MSF files distinguished by their name:
  |                      (i) Files can be ready (all homologous gene sequences grouped per file) or
  |                      not (ii) (with gene sequences dispersed in mixed files, but with specific
  |                      format - see Manual). In i) the files must be named simply as gene abbreviation
  |                      e.g. COX1.fas and in ii) name can be any simple name without spaces.
  | -p [p1,...,px]  (S1) (Optional) MSF pre-processing options. E.g. '-p rd[file.csv],rc,rg,ri[file.csv],rn'.
  |                      RD[rename.csv] = "rename sequence descriptions given in CSV" ;
  |                      RI[remove.csv] = "remove sequences from gene files given in CSV" ;
  |                      RC = "remove stop codons" ; RG = "remove gaps" ; RN = "input files not ready".
  | -a [a1,...,ax]  (S2) Estimation of multiple sequence alignments. E.g. '-a mu,cw,co' or '-a all'.
  |                      <See complete list of (case-insensitive) options using: '--helpS2'>
  | -b              (S3) (Optional) Alignment outlier detection using software as OD-SEQ and EVALMSA.
  |                      Produces a report from both softwares showing possible corroborating results.
  | -c [c1,...,cx]  (S4) (Optional) Selection of alignment refinement/consensus algorithms.
  |                      E.g. '-c tl,ta,tp' or '-c all'. <See complete list of (case-insensitive) options using: '--helpS4'>
  | -m [option]     (Optional) Enables selection of groups of MSAs that will be passed on for phylogeny estimation (and S6, S7).
  |                      Possible values are: m = "(S2)" ; i = "(S4)" ; a = "(S2) + (S4)". E.g. '-m m' or '-m a'.
  |                      Default choice, depends on previous Stage selection (either S2 or S4).
  | -t [t1,...,tx]  (S5) (Optional) Estimation of phylogenetic trees for the resulting alignments (see option -m).
  |                      E.g. '-t it,sas' or '-t all'. Specify the bootstrap replicates in front of the software code.
  |                      E.g. '-t uit1000,san100'. <See complete list of (case-insensitive) options using: '--helpS5'>
  | -s              (S6) (Optional) Phylogenetic trees comparison method using CONSEL and TREECMP software packages.
  |                      Produces reports from both cases and a final report signaling were both are common.
  | -q [q1,...,qx]  (S7) (Optional) Phylogenetic trees post-processing options. E.g. '-q all' or '-q rbs,wur'.
  |                      RBL = "remove branch lenghts" ; RBS = "remove bootsraps" ;
  |                      WUR = "write unrooted tree file" ; ALL = "all of the above".
  | -g [g1,...,gx]  (Optional) Gene abbreviations list (enabling selection of genes/files to use).
  |                      E.g. '-g COX1,CYTB,ATP6'. To be employed with option -p rn (to describe the required genes) or
  |                      to limit the use of ready genes existing in the directory specified with option -A.
  | -i [ttcode]     Translation table code as per NCBI with suitable software compatibility indication:
  |                      <See complete list of translation table options using: '--helpTTL'>
  | -n [nCPUs]      (Optional) Indicate number of available CPUs/Cores to use for running all tasks. If not given, it will be maximized.
  | -d [projdir]    Project base location path.
  | -j [projname]   (Optional) Specify project name. If option not given, one will be created.
  | -e {email}      (Optional) Email address for notification upon LMAP_S termination.
  |                      If not given, defaults to the one provided during configuration.
  | -l              (Optional) Enable logging of all selected stages/algorithms Final Status.
  |                      These logs are sent as attachments in email notifications (with option -e).
  |                      The "Algorithm", "Rank", "File" and "Time Used" are saved to a CSV file.
  |                 ------------------------------------------------------------------------
  | -h              This help.
  | --help          Show information of the different sections: MSA 'S2', MSA 'S4', PT 'S5', 'TTL', 'ASW'. E.g. 'lmap-s.pl --helpS2'.
  |                 Use 'ASW' option for listing the available integrated software and corresponding versions.
  | -v              Application version.
```

Figure S1: command-line options for *lmap-s.pl* application ("lmap-s.pl -h").

```
NAME:

  RYcode.pl - RY coding of multiple sequence alignments.

SYNOPSIS:

  RYcode.pl -i [inputfile] {-p [rycodepos]} {-o [outfile]}

DESCRIPTION:
   |  ---------------------------------------------------------------------------
   |  RY-coding of DNA multiple sequence alignments (MSA).
   |  Where A or G is found, swaps for 'R'; where C or T is found, swaps for 'Y';
   |   other characters are maintained.
   |  Modifies 3rd nucleotide positions by default, but with option -p,
   |   it is possible to perform RY-coding to different or all codon positions.

OPTIONS:
   |  ---------------------------------------------------------------------------
   | -i [inputfile]  MSA input file. Accepted formats: NEXUS or FASTA.
   | -p [codepos]    (Optinal) Specify RY-coding of codon positions,
   |                 where 'codepos' is a letter as follows:
   |                    'f': first ; 's': second ; 't': third (default) ;
   |                    'w': both first and second ; 'a': all tree positions.
   | -o [outfile]    (Optional) MSA output file name. Output maintains given input format.
   |                  If not given, it defaults to: <inputfilename>_RY[codepos].[inputfileext]
   |                 -------------------------------------------------------------
   | -h              This help
   | -v              Application version
```

Figure S2: command-line options for *RYcode.pl* application (“RYcode.pl -h”).

```
HELP SECTION S2: Alignment methods (34)
    |  ------------------------------------
    |   all => All below cases are selected
    |    co => Clustalo <default>
    |    cw => Clustalw <default>
    |    fa => FSA <default>
    |   fat => FSA TRANSLATED
    |    ga => GramAlign <default>
    |    ka => Kalign <default>
    |    ma => Mafft <default>
    |   maa => Mafft AUTO
    |    mc => MACSE <default>
    |   mcp => MACSE PSEUDOGENES
    |   mei => Mafft E-INS-i
    |   mf1 => Mafft FFT-NS-1
    |   mf2 => Mafft FFT-NS-2
    |   mfi => Mafft FFT-NS-i
    |   mgi => Mafft G-INS-i
    |   mli => Mafft L-INS-i
    |    mu => Muscle <default>
    |    op => Opal <default>
    |    pa => ProbAlign <default>
    |    pc => ProbCons <default>
    |   pcd => Prank CODON
    |   pcf => Prank CODON +F option
    |   pco => Prank CODON ONCE
    |    pk => Prank <default>
    |   pkf => Prank +F option
    |   pko => Prank ONCE
    |    tc => T_coffee <default>
    |   tkt => T_coffee KTUP_MSA
    |   tpl => T_coffee PLIB_MSA
    |   ttc => T_coffee T_COFFEE_MSA
    |    tx => Dialign-tx <default>
    |   txd => Dialign-tx DNA
    |   txt => Dialign-tx TRANSLATED
```

Figure S3: *lmap-s.pl* arguments for algorithm selection in Stage 2 (“lmap-s.pl --helpS2”).

```
HELP SECTION S4: Alignment refinement / consensus(*) methods (17)
   |  ----------------------------------------------------------------------------------
   |   all => All below cases are selected
   |    ps => PSAR-Align <default>
   |    tg => Trimal GAPPYOUT
   |   tcs => T_coffeeTCS <default> (lib generation by probcons pair-HMM 'proba_pair')
   |   gbc => Gblocks Codon <default>
   |    tl => Trimal <default>
   |    mx => MaxAlign <default>
   |    ny => Noisy <default>
   |    tp => Trimal STRICTPLUS
   |    mg => MergeAlign <default>  (*)
   |    wa => WeaveAlign <default>  (*)
   |    ta => Trimal AUTOMATED1
   |    tt => Trimal COMPARESET     (*)
   |    gb => Gblocks DNA <default>
   |   tfm => T_coffeeTCSfm ENSEMBL COMPARA (lib generation by mafft+muscle+kalign)
   |    ts => Trimal STRICT
   |   tog => T_coffeeTCSog ORIGINAL T_COFFEE (lib generation by clustalw+lalign)
```

Figure S4: *lmap-s.pl* arguments for algorithm selection in Stage 4 (“lmap-s.pl --helpS4”).

```
HELP SECTION S5: Phylogenetic methods (23)
   |  -------------------------------------------------------------------------------
   |    all[nboots] => All below cases are selected [and optional bootstraps value applied]
   |            cit => IQtree CODON TEST
   |   csit[nboots] => IQtree CODON TEST STDBOOT
   |   cuit[nboots] => IQtree CODON TEST UFBOOT
   |            dit => IQtree DNA(DEG) TEST
   |   dsit[nboots] => IQtree DNA(DEG) TEST STDBOOT
   |   duit[nboots] => IQtree DNA(DEG) TEST UFBOOT
   |            nit => IQtree DNA TEST
   |             nj => Ninja DNA
   |            nmp => MPBoot DNA
   |   nsit[nboots] => IQtree DNA TEST STDBOOT
   |   nuit[nboots] => IQtree DNA TEST UFBOOT
   |   nump[nboots] => MPBoot DNA UFBOOT
   |            rit => IQtree DNA(RY) TEST
   |   rsit[nboots] => IQtree DNA(RY) TEST STDBOOT
   |   ruit[nboots] => IQtree DNA(RY) TEST UFBOOT
   |    san[nboots] => SMS AIC + NNI
   |    sas[nboots] => SMS AIC + SPR
   |    sbn[nboots] => SMS BIC + NNI
   |    sbs[nboots] => SMS BIC + SPR
   |            tit => IQtree NT2AA TEST
   |   tsit[nboots] => IQtree NT2AA TEST STDBOOT
   |   tuit[nboots] => IQtree NT2AA TEST UFBOOT
```

Figure S5: *lmap-s.pl* arguments for algorithm selection in Stage 5 (“lmap-s.pl --helpS5”).

```
HELP SECTION TTL: NCBI Translation Table Codes (25)
    |
    |  0  => (MACSE/MPBOOT/IQTREE[DEG]) Standard / universal (transl_table=1)
    |  1  => (MACSE/MPBOOT/IQTREE[DEG]) Vertebrate Mt (transl_table=2)
    |  2  => (MACSE/MPBOOT/IQTREE[DEG]) Yeast Mt (transl_table=3)
    |  3  => (MACSE/MPBOOT/IQTREE[DEG]) Mold, Protozoan, and Coelenterate Mt and the Mycoplasma/Spiroplasma (transl_table=4)
    |  4  => (MACSE/MPBOOT/IQTREE[DEG]) Invertebrate Mt (transl_table=5)
    |  5  => (MACSE/MPBOOT/IQTREE[DEG]) Ciliate, Dasycladacean and Hexamita Nu (transl_table=6)
    |  6  => (MACSE/MPBOOT/IQTREE[DEG]) Echinoderm and Flatworm Mt (transl_table=9)
    |  7  => (MACSE/MPBOOT/IQTREE[DEG]) Euplotid Nu (transl_table=10)
    |  8  => (MACSE/MPBOOT/IQTREE[DEG]) Bacterial, Archaeal and Plant Plastid (transl_table=11)
    |  9  => (MACSE/MPBOOT/IQTREE[DEG]) Alternative Yeast Nu (transl_table=12)
    |  10 => (MACSE/MPBOOT/IQTREE[DEG]) Ascidian Mt (transl_table=13)
    |  11 => (MACSE/MPBOOT/IQTREE[DEG]) Alternative Flatworm Mt (transl_table=14)
    |  12 => (MACSE/MPBOOT) Blepharisma Nu (transl_table=15)
    |  13 => (MACSE/MPBOOT/IQTREE) Chlorophycean Mt (transl_table=16)
    |  14 => (MACSE/MPBOOT/IQTREE) Trematode Mt (transl_table=21)
    |  15 => (MACSE/MPBOOT/IQTREE) Scenedesmus obliquus Mt (transl_table=22)
    |  16 => (MACSE/MPBOOT/IQTREE) Thraustochytrium Mt (transl_table=23)
    |  17 => (MPBOOT/IQTREE) Pterobranchia Mt (transl_table=24)
    |  18 => (MPBOOT/IQTREE) Candidate Division SR1 and Gracilibacteria (transl_table=25)
    |  19 => Pachysolen tannophilus Nu (transl_table=26)
    |  20 => Karyorelict Nu (transl_table=27)
    |  21 => Condylostoma Nu (transl_table=28)
    |  22 => Mesodinium Nu (transl_table=29)
    |  23 => Peritrich Nu (transl_table=30)
    |  24 => Blastocrithidia Nu (transl_table=31)
```

Figure S6: *lmap-s.pl* arguments for translation table selection ("lmap-s.pl --helpTTL").

```
HELP SECTION ASW: Available software versions (34)
    |
    |   /home/labpc10c/bin/catpv            =>       [N/A] (v.1.20: via consel)
    |   /usr/bin/clustalo                   =>                          v.1.2.4
    |   /usr/bin/clustalw                   =>                            v.2.1
    |   /home/labpc10c/bin/consel           =>                           v.1.20
    |   /home/labpc10c/bin/Degen.pl         =>                            v.1.4
    |   /usr/bin/dialign-tx                 =>                            [N/A]
    |   /home/labpc10c/bin/EvalMSA          =>                            [N/A]
    |   /usr/bin/fsa                        =>                         v.1.15.9
    |   /home/labpc10c/bin/Gblocks          =>                            [N/A]
    |   /home/labpc10c/bin/GramAlign        =>                            v.3.0
    |   /home/labpc10c/bin/iqtree           =>                          v.1.6.2
    |   /usr/bin/java                       =>                         v.10.0.2
    |   /usr/bin/kalign                     =>                           v.2.04
    |   /home/labpc10c/bin/macse.jar        =>                           v.1.02
    |   /usr/bin/mafft                      =>              v.7.310 (2017/Mar/17)
    |   /home/labpc10c/bin/makermt          =>                           v.1.16
    |   /home/labpc10c/bin/maxalign.pl      =>                            v.1.1
    |   /home/labpc10c/bin/MergeAlign.py    =>                            [N/A]
    |   /home/labpc10c/bin/mpboot           =>                          v.1.1.0
    |   /usr/bin/muscle                     =>                         v.3.8.31
    |   /home/labpc10c/bin/ninja            =>                          v.1.2.2
    |   /home/labpc10c/bin/noisy            =>                            [N/A]
    |   /home/labpc10c/bin/OD-seq           =>                            v.1.0
    |   /home/labpc10c/bin/opal             =>                          v.2.1.3
    |   /usr/bin/prank                      =>                         v.170427
    |   /usr/bin/probalign                  =>                            v.1.4
    |   /usr/bin/probcons                   =>                           v.1.12
    |   /home/labpc10c/bin/PSARAlign.pl     =>                            [N/A]
    |   /home/labpc10c/bin/RYcode.pl        =>           v.1.0.0 Mar 30th, 2018
    |   /home/labpc10c/bin/sms.sh           =>                            [N/A]
    |   /usr/bin/t_coffee                   =>                  v.11.00.8cbe486
    |   /home/labpc10c/bin/TreeCmp.jar      =>                      v.1.1-b308
    |   /home/labpc10c/bin/trimal           =>     v.1.4.re22 build[2015-05-21]
    |   /home/labpc10c/bin/WeaveAlign.jar   =>                          v.1.2.1
```

Figure S7: *lmap-s.pl* display of available integrated software ("lmap-s.pl --helpASW").
