# Supplementary material for: LMAP_S: Lightweight Multigene Alignment and Phylogeny eStimation
Source: BMC Bioinformatics. 2019 Dec 30;20:739. doi: 10.1186/s12859-019-3292-5 (PMC6937843; doi:10.1186/s12859-019-3292-5)
Supplement: Supplementary file 3 — Additional file 3. Table extending Table 1 information. Shows absolute identification assigned to algorithms and respective code abbreviation, which enables their selection into LMAP_S stages. [file 12859_2019_3292_MOESM3_ESM.pdf]

Table S1: Table 1 extended with algorithms assigned absolute identification and respective code abbreviation, which enables its selection in LMAP_S stages.

LMAP_S Stage	Integrated Software	Algorithms Implemented	Algorithms Identification (Code) (respectively)
**Stage 2 (AE)**	Clustal Omega (v.1.2.1)	*Default*	CLUSTALO (CO)
	ClustalW (v.2.1)	*Default*	CLUSTALW (CW)
	Dialign-tx (v.1.0.2)	(3) Dialign-tx *Default* ; Dialign-tx -D option ; Dialign-tx -T option	DIALIGN-TX (TX) ; DIALIGN-TXD (TXD) ; DIALIGN-TXT (TXT)
	FSA (v.1.15.9)	(2) FSA *Default* ; FSA with 'nucprot' option	FSA (FA) ; FSANP (FAT)
	GramAlign (v.3.0)	*Default*	GRAMALIGN (GA)
	Kalign (v.2.0.4)	*Default*	KALIGN (KA)
	MACSE (v.1.0.2)	(2) MACSE *Default*, MACSE with pseudogene alignment.	MACSE (MC) ; MACSEP (MCP)
	MAFFT (v.7.271)	(8) MAFFT *Default*, MAFFT with 'auto' option, MAFFT E-INS-I, MAFFT FFT-NS-1, MAFFT FFT-NS-2, MAFFT FFT-NS-I, MAFFT G-INS-I, MAFFT L-INS-I	MAFFT (MA) ; MAFFTA (MAA); MAFFTEI (MEI) ; MAFFTF1 (MF1); MAFFTF2 (MF2) ; MAFFTFI (MFI) ; MAFFTGI (MGI) ; MAFFTLI (MLI)
	MUSCLE (v.3.8.31)	*Default*	MUSCLE (MU)
	Opal (v.2.1.3)	*Default*	OPAL (OP)
	Prank (v.150803)	(6) Prank *Default*, Prank +F option, Prank 'once' option, Prank Codon, Prank Codon +F option, Prank Codon 'once' option.	PRANK (PK) ; PRANKF (PKF); PRANKO (PKO); PRANKCD (PCD); PRANKCDF (PCF); PRANKCDO (PCO)
	ProbAlign (v.1.4)	*Default*	PROBALIGN (PA)
	ProbCons (v.1.12)	*Default*	PROBCONS (PC)
	T-COFFEE (v.11.00.8cbe486)	(4) *Default* 'PROBA_PAIR' ; 'T_COFFEE_MSA', 'KTUP_MSA', 'PLIB_MSA'	TCOFFEE (TC); TCOFFEEKT (TTC) ; TCOFFEEKT (TKT) ; TCOFFEEPL (TPL)
**Stage 3 (AOD)**	OD-Seq (v.1.0)	*Default*	N.A. (N.A.)
	EvalMSA (v.1.0)	*Default*	N.A. (N.A.)
**Stage 4**	Gblocks (v.0.91b)	(2) *Default DNA ; Default CODON*	GBLOCKS (GB) ; GBLOKCS (GBC)

**(ARC)**	MaxAlign (v.1.1)	*Default*	MAXALIGN (MX)
	MergeAlign (n.f.)	*Default* (#)	MERGEALIGN (MG)
	Noisy (v.1.5.12)	*Default*	NOISY (NY)
	PSAR-Align (v.1.0)	Default	PSARALIGN (PS)
	TCS (T-Coffee)	(3) TCS, TCS_original, TCS_FM	TCS (TCS); TCSFM (TFM); TCSOG (TOG)
	TrimAl (v.1.4)	(6) TrimAl *Default*, TrimAl 'automated1', TrimAl 'gappyout', TrimAl 'strictplus', TrimAl 'strict', TrimAl 'compareset' (#)	TRIMAL (TL) ; TRIMALA (TA); TRIMALG (TG) ; TRIMALP (TP); TRIMALS (TS) ; TRIMALC (TT)
	WeaveAlign (v.1.2.1)	*Default* (#)	WEAVEALIGN (WA)
**Stage 5 (PE)**	IQ-TREE (v.1.6.2)	(15) IQ-TREE D**N**A, IQ-TREE DNA (**D**EG), IQ-TREE DNA (**R**Y), IQ-TREE **C**ODON, IQ-TREE N**T**2AA. Each case is available for *Default* and *Standard* / *UFBoot* Bootstraps	**N**IQTREE (NIT) ; **N**SBIQTREE (NSIT) ; **N**UBIQTREE (NUIT); **D**IQTREE (DIT); **D**SBIQTREE (DSIT); **D**UBIQTREE (DUIT); **R**IQTREE (RIT); **R**SBIQTREE (RSIT); **R**UBIQTREE (RUIT); **C**IQTREE (CIT); **C**SBIQTREE (CSIT); **C**UBIQTREE (CUIT); **T**IQTREE (TIT); **T**SBIQTREE (TSIT); **T**UBIQTREE (TUIT)
	MPBoot (v.1.1.0)	(2) MPBoot D**N**A. Each case is available for *Default* and "*UFBoot*" Bootstraps	**N**MPBOOT (NMP) ; **N**UBMPBOOT (NUMP)
	Ninja (v.1.2.2)	*Default*	NINJA (NJ)
	SMS (v.1.8.1)	(4) AIC + NNI, AIC + SPR, BIC + NNI, BIC + SPR	SMSAN (SAN) ; SMSAS (SAS) ; SMSBN (SBN) ; SMSBS (SBS)
	Degen (v.1.4)	*Default*	DEG (N.A.)
	RYcode (v.1.0.0)	*Default*	RYT (DEFAULT (*)) (N.A.)
**Stage 6 (PCC)**	CONSEL (v.1.2.0)	*Default (includes makermt, consel and catpv)*	ICONSEL (N.A.)
	TreeCmp (v.1.1)	*Default*	TREECMP MP (N.A.) ; TREECMP RF (N.A.)

Legend: N.A. – Not applicable. (*) *RYcode.pl* performs coding of the third-position by default, hence the 'T' in 'RYt'.
For more information, see also LMAP_S Manual.
