# Supplementary material for: LMAP_S: Lightweight Multigene Alignment and Phylogeny eStimation
Source: BMC Bioinformatics. 2019 Dec 30;20:739. doi: 10.1186/s12859-019-3292-5 (PMC6937843; doi:10.1186/s12859-019-3292-5)
Supplement: Supplementary file 4 — Additional file 4. Flowchart illustrating the PCC method. Shows the several steps of the method starting with the PE Stage data until final consensus reports. [file 12859_2019_3292_MOESM4_ESM.pdf]

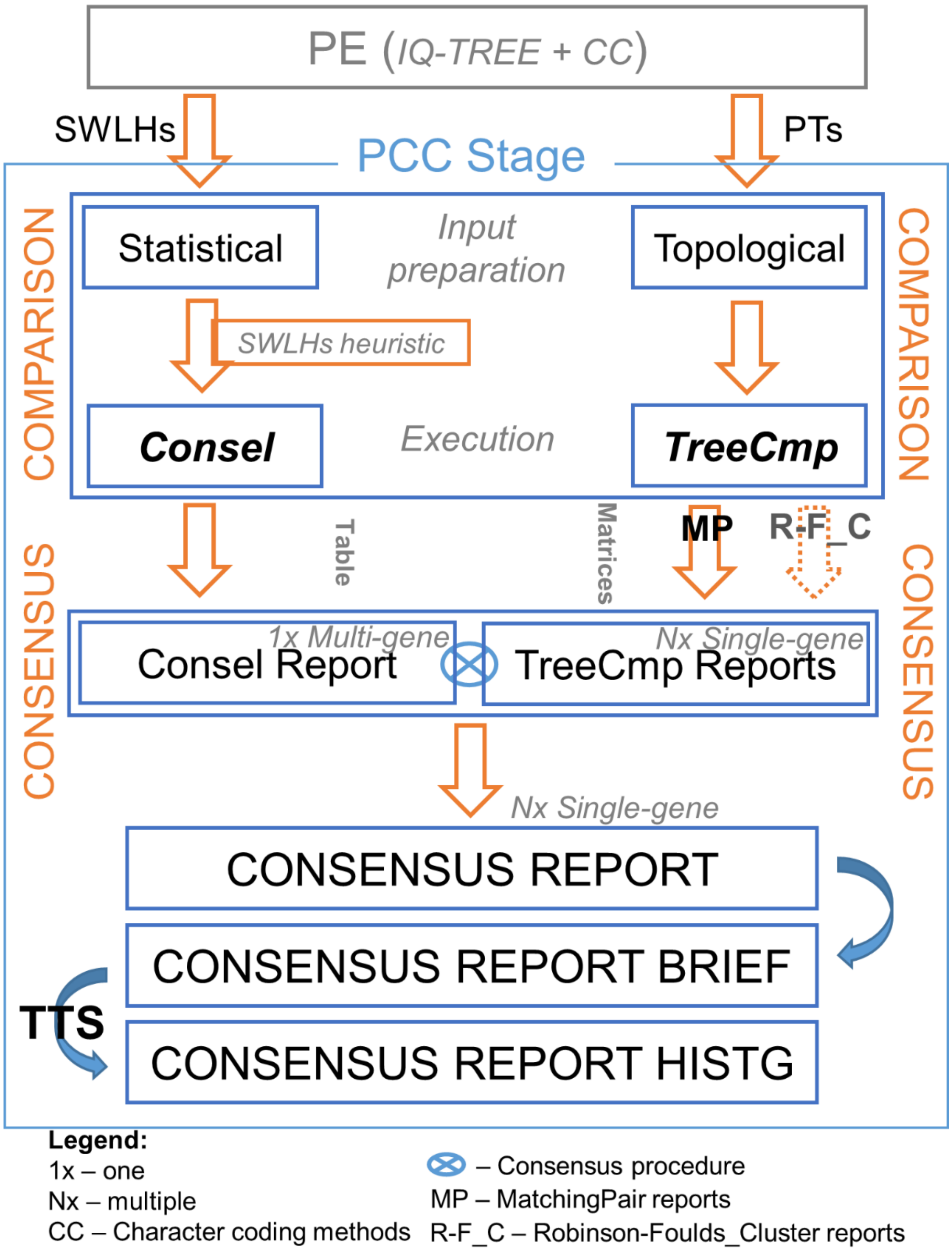

Figure S1: Flowchart illustrating the PCC method, where six reports are produced: one Consel, two TreeCmp (MP and R-F_C) and three consensuses (where, BRIEF is a condensed report and HISTG is the final histogram report).
