# Supplementary material for: LMAP_S: Lightweight Multigene Alignment and Phylogeny eStimation
Source: BMC Bioinformatics. 2019 Dec 30;20:739. doi: 10.1186/s12859-019-3292-5 (PMC6937843; doi:10.1186/s12859-019-3292-5)
Supplement: Supplementary file 6 — Additional file 6. LMAP_S case study analyses of the Cephalopoda mitochondrial genes. (File 1): Description of experiments, results and discussion. (File 2): LMAP_S and TreeCmp command-lines with additional benchmarking. (File 3): Tables with LMAP_S consensus histogram reports from CephaResults. (File 4): Tables with LMAP_S consensus histogram reports from CephaResultsARC. (File 5): Figures showing side-by-side consensus strategies charts comparisons. (File 6): Tables with results of the topological comparisons. (File 7): Final and original LMAP_S results (PTs and Reports). (File 8): Bash scripts used to generate the TreeCmp input files. [file 12859_2019_3292_MOESM6_ESM.zip › Additional file 6/File 1.docx]

**Extended Case Study Legend**

In this document we performed a case study comparing the methodology employed in the study of Cephalopoda mitochondrial dataset with that of our LMAP_S software. This is performed to attempt to find improvements in the reported study methodology and results (i.e., alignment and phylogeny estimations), demonstrating the usefulness of LMAP_S.

According to the study, the datasets and their transformations result in the same topology, hence it becomes negligible which dataset (“left” or “right” copies) is being used. Moreover, since the Bayesian and ML (RY-coded) concatenated topologies are described to be the same, we have opted to use the ML topology (MLTS) to ensure comparability with LMAP_S workflow and results.

In the following sections, we briefly describe the experiments, results and discussion for this case study. Additional brief introduction and conclusions are found in the corresponding the LMAP_S article section.

In this document we refer to several accompanying files that are part of this case study, please see below:

**File 1.docx**: This document. Description of the experiments, results and discussion.

**File 2.txt**: LMAP_S and TreeCmp command-lines employed and additional benchmarking results for the two LMAP_S executions.

**File 3.xlsx**: LMAP_S consensus histogram reports from *CephaResults*.

**File 4.xlsx**: LMAP_S consensus histogram reports from *CephaResultsARC*.

**File 5.ppt**: Side-by-side consensus strategies charts comparison between *CephaResults* and *CephaResultsARC.*

**File 6.xlsx**: Topological comparison results of optimal consensus strategies (OCS; both executions) with ML topology from the study.

**File 7.zip**: Final LMAP_S results (PTs and Reports) from both *CephaResults* and *CephaResultsARC*.

**File 8.zip**: The bash scripts (*All_cat16.sh - CephaResults* and *All_cat134.sh - CephaResultsARC*) used to generate TreeCmp input files.

**Materials and Methods**

We have employed LMAP_S to run the Cephalopoda dataset in the simplest manner attempting to closely resemble the methodology of the original study and thus acquire comparable results. Proceeding in this manner, we attempt to assert if this simplest and similar execution (File 2 – I.1) could provide identical or different results in terms of the MSA and CC algorithms selection and final topologies. Additionally, to enable further comparison of results we have employed a second LMAP_S execution (File 2 – II.1). This was performed to understand how these second results would be comparable with the first. Specifically, if by additionally performing refinement steps (ARC stage), further improvements could be found.

Finally, we have performed independent topological comparisons with both executions results hereby including the published ML topology in each case (Files 3, 4 and 6). Before proceeding to these experiments the MLTS was modified to incorporate full taxa names (recall that PhyML traditionally limits taxa descriptions to a maximum of 10 characters).

These experiments were accomplished with the same workstation as described in the article “Example Dataset and Benchmarking” section. Following we provide detailed steps.

1. The study dataset here used (available under request) consisted of 13 mitochondrial genes encompassing 17 Cephalopoda species. The gene MSAs provided in Nexus format are: ATP6, ATP8, CYTB, COX1, COX2, COX3, ND1, ND2, ND3, ND4, ND4L, ND5 and ND6. They were de-aligned and converted to FASTA format with Seaview v.4.6.4 software. The MLTS Newick file was manually edited; we replaced the existing incomplete identification with complete taxa identification.
2. Executed two LMAP_S command-lines (see File 2) with the same dataset: (I.1) without ARC Stage (*CephaResults*) and (II.1) with ARC Stage (*CephaResultsARC*). To ensure justified results, we have employed all available algorithms in the AE and ARC Stages.
3. Compiled all the OCS for each LMAP_S execution results (File 3 - *CephaResults* and File 4 - *CephaResultsARC*).
4. Prepared topological comparison (using TreeCmp software) by including the MLTS in the first position of each input file (File 2 – (I.2) and (II.2)). This was accomplished by collecting all Newick files in two separate input files (with the bash scripts): TREECMP_16PT_INPUT.nwk and TREECMP_134PT_INPUT.nwk. All the topologies have been placed in a specific order, also shown in File 6.
5. These were independently provided and executed with TreeCmp using the same command-line settings as by default available in LMAP_S integrated software configuration file (please see LMAP_S Manual).

**Results and Discussion**

The first premise was to test if the (MUSCLE + RY-coding) methodology from the study could be proven by LMAP_S results. As expected we have acquired different results from both LMAP_S executions. In *CephaResults* (File 3) it is possible to verify that only in two genes (ATP6 and ND5) two OCS were found with the same maximum TTS (Table C1). By looking at Table C1, MUSCLE algorithm is not found in any OCS, contrarily to RY-coding, which is found twice in 15 OCS (13%). Clearly, we have not detected any MUSCLE + RY-coding strategy.

**Table C1. Resulting OCS from *CephaResults*.**

| **Strategy** | **TTS** |
| --- | --- |
| *** # ATP6_FSANP_2AA_UB # *** | 10 |
| *** # ATP6_MAFFTGI_CDN_UB # *** | 10 |
| *** # ATP8_MAFFTGI_DNA_UB # *** | 18 |
| *** # COX1_PRANK_DNA_UB # *** | 7 |
| *** # COX2_FSANP_DNA_UB # *** | 37 |
| *** # COX3_MAFFTF1_DNA_UB # *** | 34 |
| *** # CYTB_KALIGN_DNA_UB # *** | 37 |
| *** # ND1_MAFFTEI_DEG_UB # *** | 42 |
| *** # ND2_MAFFTA_DNA_UB # *** | 21 |
| *** # ND3_OPAL_DNA_UB # *** | 6 |
| *** # ND4_MAFFTLI_DEG_UB # *** | 16 |
| *** # ND4L_CLUSTALW_DNA_UB # *** | 31 |
| *** # ND5_GRAMALIGN_RYt_UB # *** | 30 |
| *** # ND5_MACSE_DNA_UB # *** | 30 |
| *** # ND6_GRAMALIGN_RYt_UB # *** | 23 |

In *CephaResultsARC* (File 4), the number of detected consensus strategies has increased, mainly those with the equal maximum TTS (see side-by-side comparison in File 5). We suggest that this increase could be explained by the improvement in MSA phylogenetic signal enforced by the ARC Stage. Anyhow, we cannot neglect that the analyzed data size increased from one execution to another. But as seen with the Example dataset, this increase in size does not necessarily result in more than one OCS per gene. By looking at the detected OCS (File 4), the same conclusion is reached. MUSCLE is only found once (in CYTB) and combined with DEG-coding. The RY-coding is found 33 times in a total of 133 OCS (24,8%). Despite this and the ARC Stage inclusion, strategies combining MUSCLE and RY-coding were not found.

The second premise was to provide evidence that LMAP_S OCS conveys improvements over the topological results from the study. As the ML topology has been provided to the input file in the first position/entry, TreeCmp registers the comparisons of the ML tree with LMAP_S phylogenies in the first place (File 6). By looking at the number of OCS from *CephaResults*, it is not entirely possible to compare the topologies in each gene, where most of them are unique OCS. Here, we have two cases, ATP6 and ND5 genes. Contrarily to ND5, the ATP6 OCS topologies are the only case where they are found to be different (File 6). However, gene-by-gene OCS topologies comparison is possible and demonstrated with *CephaResultsARC*. In fact, despite the differences in the chains of algorithms, each gene has the same topology (File 6). Under these circumstances, this additionally supports the optimality of the detected OCS.

Additionally, it is noticeable that regardless of the different LMAP_S executions, the average of the MP scores in the group of comparisons including the MLTS, was significantly greater compared to that of comparisons among LMAP_S OCS topologies alone. Here, the first group registered an average MP score of **94,67** (with MLTS) and **48,54** (without MLTS) for the *CephaResults*; and of **94.07** (with MLTS) and **45,77** (without MLTS) for the *CephaResultsARC*. Additionally, it is visible that both values are close to each other in the two groups, (i) with and (ii) without MLTS, showing reciprocal support. Moreover, they show that the concatenated MLTS has worse MP scores than that of LMAP_S (closer to ideal zero MP score). From this we can infer that applying the *CephaResults* simpler workflow could be sufficient. However, despite the slight average score improvement, the *CephaResultsARC* still provides better results. Here it could be argued that the several zero MP scores repeated for same gene topology (several OCS; File 6) could be misleading the MP average score, but removing them, would not enable a fair comparison.

In either LMAP_S execution, contrarily to the study methodology, we have detected different strategies for each gene. Whereas, the study methodology has not been inferred as an OCS. These results (additionally supported by the Example Dataset) have proven wrong our first premise. Additionally, the different strategies found for each gene, clearly contradict the usual application of the same methodology to all genes.

These conclusions are additionally supported by the second premise, hereby confirming the OCS relevance. Here, the overall topology comparison experiments and the MP average scores, provide evidence that (i) the application of the same algorithms to all genes is not valid, and that (ii) the multi-gene concatenation strategy is not the most powerful approach. Hence, in topological terms, LMAP_S provides improvements over the MLTS, thus demonstrating the relevance of the detected OCS. These results have proven correct in our second premise, despite the difficult dataset here employed. Overall, they provide strong evidence that LMAP_S is a well-supported and useful software.
