# Supplementary material for: LMAP_S: Lightweight Multigene Alignment and Phylogeny eStimation
Source: BMC Bioinformatics. 2019 Dec 30;20:739. doi: 10.1186/s12859-019-3292-5 (PMC6937843; doi:10.1186/s12859-019-3292-5)
Supplement: Supplementary file 6 — Additional file 6. LMAP_S case study analyses of the Cephalopoda mitochondrial genes. (File 1): Description of experiments, results and discussion. (File 2): LMAP_S and TreeCmp command-lines with additional benchmarking. (File 3): Tables with LMAP_S consensus histogram reports from CephaResults. (File 4): Tables with LMAP_S consensus histogram reports from CephaResultsARC. (File 5): Figures showing side-by-side consensus strategies charts comparisons. (File 6): Tables with results of the topological comparisons. (File 7): Final and original LMAP_S results (PTs and Reports). (File 8): Bash scripts used to generate the TreeCmp input files. [file 12859_2019_3292_MOESM6_ESM.zip › Additional file 6/File 5.pptx]

## Slide 1
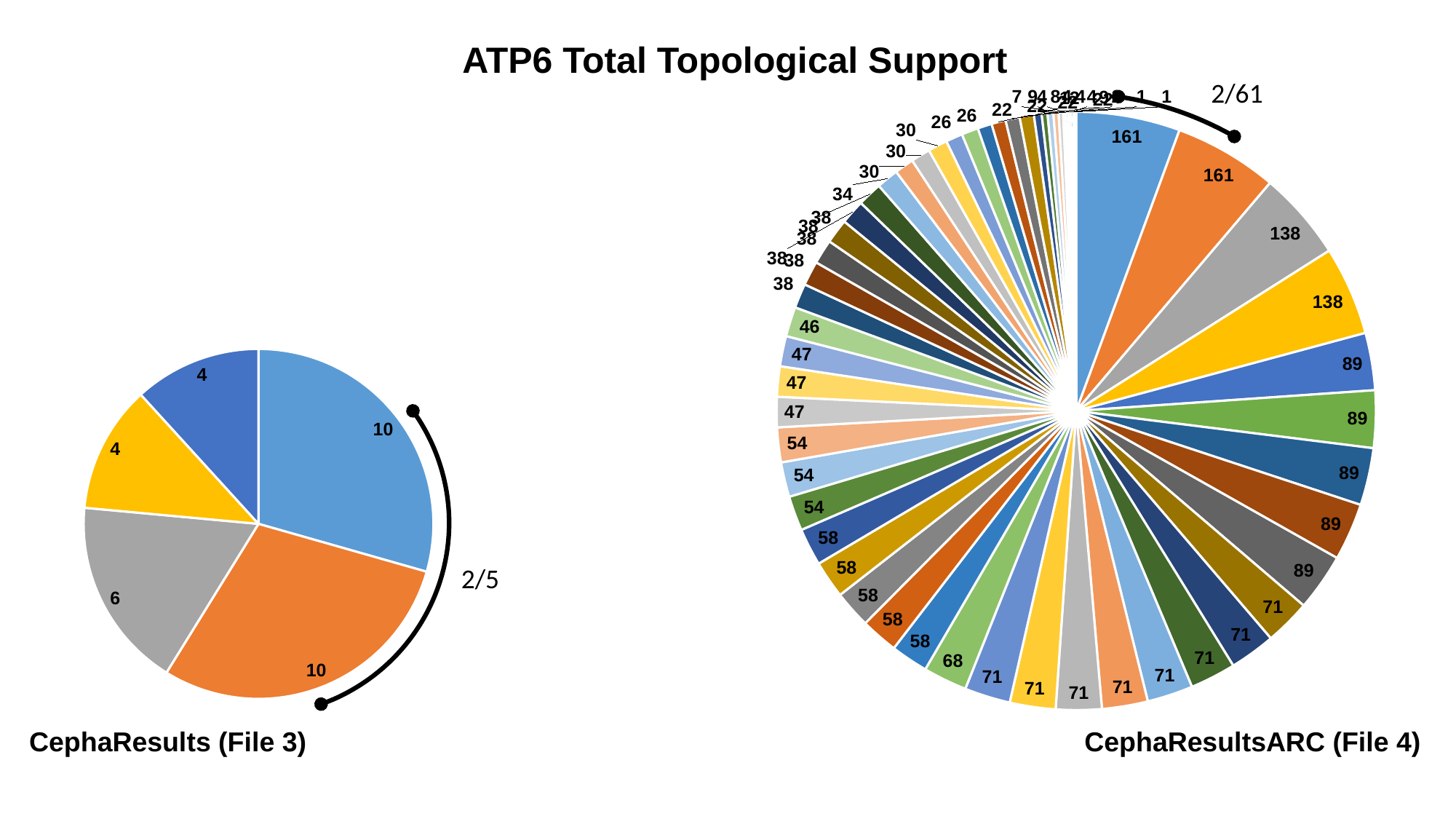

ATP6 Total Topological Support
2/61
### Chart
| Category | Total Topological Support |
|---|---|
| *** # ATP6_FSA_PSARALIGN_DNA_UB # *** | 161.0 |
| *** # ATP6_MAFFTA_TCSFMw_DNA_UB # *** | 161.0 |
| *** ATP6_ALL_TRIMALC_DNA_UB *** | 138.0 |
| *** ATP6_ALL_WEAVEALIGN_DNA_UB *** | 138.0 |
| *** # ATP6_FSA_TRIMALG_CDN_UB # *** | 89.0 |
| *** # ATP6_MAFFTA_MAXALIGN_CDN_UB # *** | 89.0 |
| *** # ATP6_MAFFTA_TCSFMf_CDN_UB # *** | 89.0 |
| *** # ATP6_MAFFTEI_TCSf_CDN_UB # *** | 89.0 |
| *** # ATP6_MAFFTEI_TRIMAL_CDN_UB # *** | 89.0 |
| *** # ATP6_CLUSTALO_PSARALIGN_DEG_UB # *** | 71.0 |
| *** # ATP6_FSANP_TCSOGw_RYt_UB # *** | 71.0 |
| *** # ATP6_FSANP_TRIMAL_DEG_UB # *** | 71.0 |
| *** # ATP6_FSA_TCSw_RYt_UB # *** | 71.0 |
| *** # ATP6_MAFFTGI_TRIMALA_DEG_UB # *** | 71.0 |
| *** # ATP6_MAFFTLI_TCSw_DEG_UB # *** | 71.0 |
| *** # ATP6_MAFFTLI_TCSw_DNA_UB # *** | 71.0 |
| *** # ATP6_MAFFTLI_TRIMALG_DEG_UB # *** | 71.0 |
| *** ATP6_ALL_MERGEALIGN_DEG_UB *** | 68.0 |
| *** # ATP6_FSANP_TCSFMf_2AA_UB # *** | 58.0 |
| *** # ATP6_GRAMALIGN_PSARALIGN_2AA_UB # *** | 58.0 |
| *** # ATP6_GRAMALIGN_TRIMAL_2AA_UB # *** | 58.0 |
| *** # ATP6_MAFFTEI_MAXALIGN_2AA_UB # *** | 58.0 |
| *** # ATP6_MAFFTGI_TCSf_2AA_UB # *** | 58.0 |
| *** # ATP6_MACSE_TRIMALA_DNA_UB # *** | 54.0 |
| *** # ATP6_MAFFTLI_GBLOCKS_DNA_UB # *** | 54.0 |
| *** # ATP6_PRANKCDO_GBLOCKSC_DNA_UB # *** | 54.0 |
| *** # ATP6_MACSE_GBLOCKS_2AA_UB # *** | 47.0 |
| *** # ATP6_MAFFTA_GBLOCKSC_2AA_UB # *** | 47.0 |
| *** # ATP6_MAFFTA_TRIMALA_2AA_UB # *** | 47.0 |
| *** ATP6_ALL_TRIMALC_CDN_UB *** | 46.0 |
| *** # ATP6_GRAMALIGN_TCSOGw_DNA_UB # *** | 38.0 |
| *** # ATP6_MAFFTEI_TCSFMf_DNA_UB # *** | 38.0 |
| *** # ATP6_MAFFTGI_MAXALIGN_DNA_UB # *** | 38.0 |
| *** # ATP6_MAFFTGI_TRIMAL_DNA_UB # *** | 38.0 |
| *** # ATP6_MAFFTLI_TCSf_DNA_UB # *** | 38.0 |
| *** ATP6_ALL_TRIMALC_2AA_UB *** | 38.0 |
| *** # ATP6_MAFFTEI_GBLOCKSC_CDN_UB # *** | 34.0 |
| *** # ATP6_FSANP_TRIMALG_RYt_UB # *** | 30.0 |
| *** # ATP6_FSA_TRIMALA_RYt_UB # *** | 30.0 |
| *** # ATP6_PRANKCDO_GBLOCKSC_RYt_UB # *** | 30.0 |
| *** # ATP6_FSA_TCSFMf_DEG_UB # *** | 26.0 |
| *** # ATP6_FSA_TCSf_DEG_UB # *** | 26.0 |
| *** # ATP6_FSA_PSARALIGN_RYt_UB # *** | 22.0 |
| *** # ATP6_FSA_TCSFMf_RYt_UB # *** | 22.0 |
| *** # ATP6_FSA_TCSf_RYt_UB # *** | 22.0 |
| *** # ATP6_FSA_TRIMAL_RYt_UB # *** | 22.0 |
| *** # ATP6_MACSE_NOISY_RYt_UB # *** | 12.0 |
| *** # ATP6_FSANP_TRIMALS_RYt_UB # *** | 9.0 |
| *** # ATP6_MACSE_TRIMALP_RYt_UB # *** | 9.0 |
| *** # ATP6_FSA_MAXALIGN_RYt_UB # *** | 8.0 |
| *** # ATP6_MACSE_TCSFMw_RYt_UB # *** | 7.0 |
| *** # ATP6_MAFFTA_GBLOCKS_DEG_UB # *** | 4.0 |
| *** # ATP6_MAFFTLI_NOISY_DNA_UB # *** | 4.0 |
| *** # ATP6_OPAL_TRIMALP_DNA_UB # *** | 4.0 |
| *** ATP6_ALL_TRIMALC_RYt_UB *** | 4.0 |
| *** # ATP6_MACSE_TRIMALP_DEG_UB # *** | 2.0 |
| *** # ATP6_MAFFTLI_NOISY_DEG_UB # *** | 1.0 |
| *** # ATP6_PRANKCDO_GBLOCKSC_DEG_UB # *** | 1.0 |
| # ATP6_GRAMALIGN_GBLOCKS_RYt_UB # | 0.0 |
| # ATP6_MAFFTEI_TCSFMw_DEG_UB # | 0.0 |
| # ATP6_PRANKO_TRIMALS_DNA_UB # | 0.0 |
### Chart
| Category | Total Topological Support |
|---|---|
| *** # ATP6_FSANP_2AA_UB # *** | 10.0 |
| *** # ATP6_MAFFTGI_CDN_UB # *** | 10.0 |
| *** # ATP6_FSANP_DEG_UB # *** | 6.0 |
| *** # ATP6_FSANP_RYt_UB # *** | 4.0 |
| *** # ATP6_GRAMALIGN_DNA_UB # *** | 4.0 |
2/5
CephaResults (File 3)
CephaResultsARC (File 4)

## Slide 2
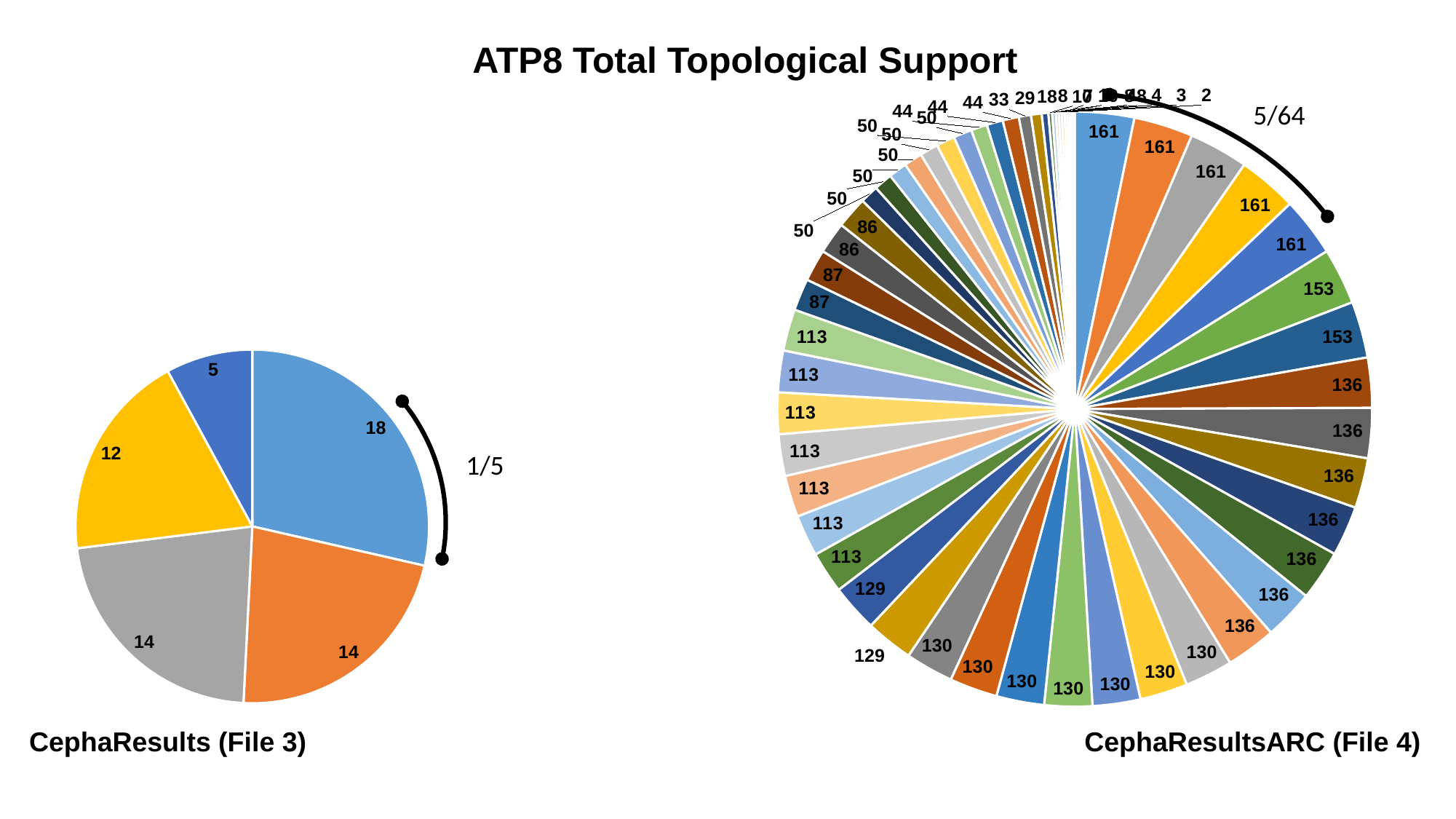

ATP8 Total Topological Support
### Chart
| Category | Total Topological Support |
|---|---|
| *** # ATP8_FSANP_GBLOCKSC_DNA_UB # *** | 161.0 |
| *** # ATP8_MAFFTGI_MAXALIGN_DNA_UB # *** | 161.0 |
| *** # ATP8_MAFFTGI_TCSf_DNA_UB # *** | 161.0 |
| *** # ATP8_MAFFTGI_TRIMAL_DNA_UB # *** | 161.0 |
| *** # ATP8_MAFFT_GBLOCKS_DNA_UB # *** | 161.0 |
| *** ATP8_ALL_TRIMALC_DNA_UB *** | 153.0 |
| *** ATP8_ALL_TRIMALC_RYt_UB *** | 153.0 |
| *** # ATP8_MACSE_GBLOCKS_DEG_UB # *** | 136.0 |
| *** # ATP8_MAFFTF1_TRIMAL_DEG_UB # *** | 136.0 |
| *** # ATP8_MAFFTF2_TCSFMf_DEG_UB # *** | 136.0 |
| *** # ATP8_MAFFTFI_PSARALIGN_DEG_UB # *** | 136.0 |
| *** # ATP8_MAFFTGI_MAXALIGN_DEG_UB # *** | 136.0 |
| *** # ATP8_MAFFTLI_TCSf_DEG_UB # *** | 136.0 |
| *** # ATP8_OPAL_GBLOCKSC_DEG_UB # *** | 136.0 |
| *** # ATP8_CLUSTALO_GBLOCKSC_2AA_UB # *** | 130.0 |
| *** # ATP8_FSANP_TCSFMf_2AA_UB # *** | 130.0 |
| *** # ATP8_MACSE_GBLOCKS_2AA_UB # *** | 130.0 |
| *** # ATP8_MACSE_PSARALIGN_2AA_UB # *** | 130.0 |
| *** # ATP8_MACSE_TCSf_2AA_UB # *** | 130.0 |
| *** # ATP8_MAFFTA_MAXALIGN_2AA_UB # *** | 130.0 |
| *** # ATP8_MAFFTA_TRIMAL_2AA_UB # *** | 130.0 |
| *** ATP8_ALL_TRIMALC_2AA_UB *** | 129.0 |
| *** ATP8_ALL_WEAVEALIGN_2AA_UB *** | 129.0 |
| *** # ATP8_KALIGN_GBLOCKS_CDN_UB # *** | 113.0 |
| *** # ATP8_MAFFTEI_PSARALIGN_CDN_UB # *** | 113.0 |
| *** # ATP8_MAFFTFI_GBLOCKSC_CDN_UB # *** | 113.0 |
| *** # ATP8_MAFFTFI_TRIMAL_CDN_UB # *** | 113.0 |
| *** # ATP8_MAFFTGI_TCSf_CDN_UB # *** | 113.0 |
| *** # ATP8_OPAL_MAXALIGN_CDN_UB # *** | 113.0 |
| *** # ATP8_OPAL_TCSFMf_CDN_UB # *** | 113.0 |
| *** ATP8_ALL_MERGEALIGN_DEG_UB *** | 87.0 |
| *** ATP8_ALL_TRIMALC_DEG_UB *** | 87.0 |
| *** ATP8_ALL_TRIMALC_CDN_UB *** | 86.0 |
| *** ATP8_ALL_WEAVEALIGN_CDN_UB *** | 86.0 |
| *** # ATP8_GRAMALIGN_GBLOCKSC_RYt_UB # *** | 50.0 |
| *** # ATP8_GRAMALIGN_MAXALIGN_RYt_UB # *** | 50.0 |
| *** # ATP8_GRAMALIGN_PSARALIGN_RYt_UB # *** | 50.0 |
| *** # ATP8_KALIGN_TRIMAL_RYt_UB # *** | 50.0 |
| *** # ATP8_MACSE_GBLOCKS_RYt_UB # *** | 50.0 |
| *** # ATP8_MACSE_TCSFMf_RYt_UB # *** | 50.0 |
| *** # ATP8_OPAL_TCSf_RYt_UB # *** | 50.0 |
| *** # ATP8_CLUSTALO_TCSFMw_RYt_UB # *** | 44.0 |
| *** # ATP8_KALIGN_TCSFMw_DEG_UB # *** | 44.0 |
| *** # ATP8_MAFFTF1_TCSFMw_DNA_UB # *** | 44.0 |
| *** ATP8_ALL_WEAVEALIGN_RYt_UB *** | 33.0 |
| *** # ATP8_MAFFTFI_TCSFMf_DNA_UB # *** | 29.0 |
| *** # ATP8_MAFFTLI_PSARALIGN_DNA_UB # *** | 18.0 |
| *** # ATP8_MAFFTF1_TCSw_DNA_UB # *** | 10.0 |
| *** # ATP8_OPAL_TCSw_DNA_UB # *** | 10.0 |
| *** # ATP8_CLUSTALO_TCSw_RYt_UB # *** | 8.0 |
| *** # ATP8_KALIGN_TCSw_DEG_UB # *** | 8.0 |
| *** ATP8_ALL_WEAVEALIGN_DNA_UB *** | 8.0 |
| *** # ATP8_CLUSTALO_TRIMALS_DEG_UB # *** | 7.0 |
| *** # ATP8_MAFFTA_TCSOGw_DNA_UB # *** | 7.0 |
| *** # ATP8_TCOFFEETC_NOISY_DEG_UB # *** | 4.0 |
| *** # ATP8_TCOFFEETC_NOISY_DNA_UB # *** | 4.0 |
| *** # ATP8_KALIGN_TCSOGw_RYt_UB # *** | 3.0 |
| *** # ATP8_TCOFFEE_NOISY_RYt_UB # *** | 2.0 |
| # ATP8_GRAMALIGN_TRIMALS_RYt_UB # | 0.0 |
| # ATP8_OPAL_TCSOGw_DEG_UB # | 0.0 |
| # ATP8_OPAL_TRIMALS_DNA_UB # | 0.0 |
| # ATP8_PRANKCDO_TRIMALP_DEG_UB # | 0.0 |
| # ATP8_PRANKCDO_TRIMALP_RYt_UB # | 0.0 |
| # ATP8_PRANK_TRIMALP_DNA_UB # | 0.0 |
5/64
### Chart
| Category | Total Topological Support |
|---|---|
| *** # ATP8_MAFFTGI_DNA_UB # *** | 18.0 |
| *** # ATP8_CLUSTALO_2AA_UB # *** | 14.0 |
| *** # ATP8_GRAMALIGN_DEG_UB # *** | 14.0 |
| *** # ATP8_CLUSTALO_CDN_UB # *** | 12.0 |
| *** # ATP8_CLUSTALO_RYt_UB # *** | 5.0 |
1/5
CephaResults (File 3)
CephaResultsARC (File 4)

## Slide 3
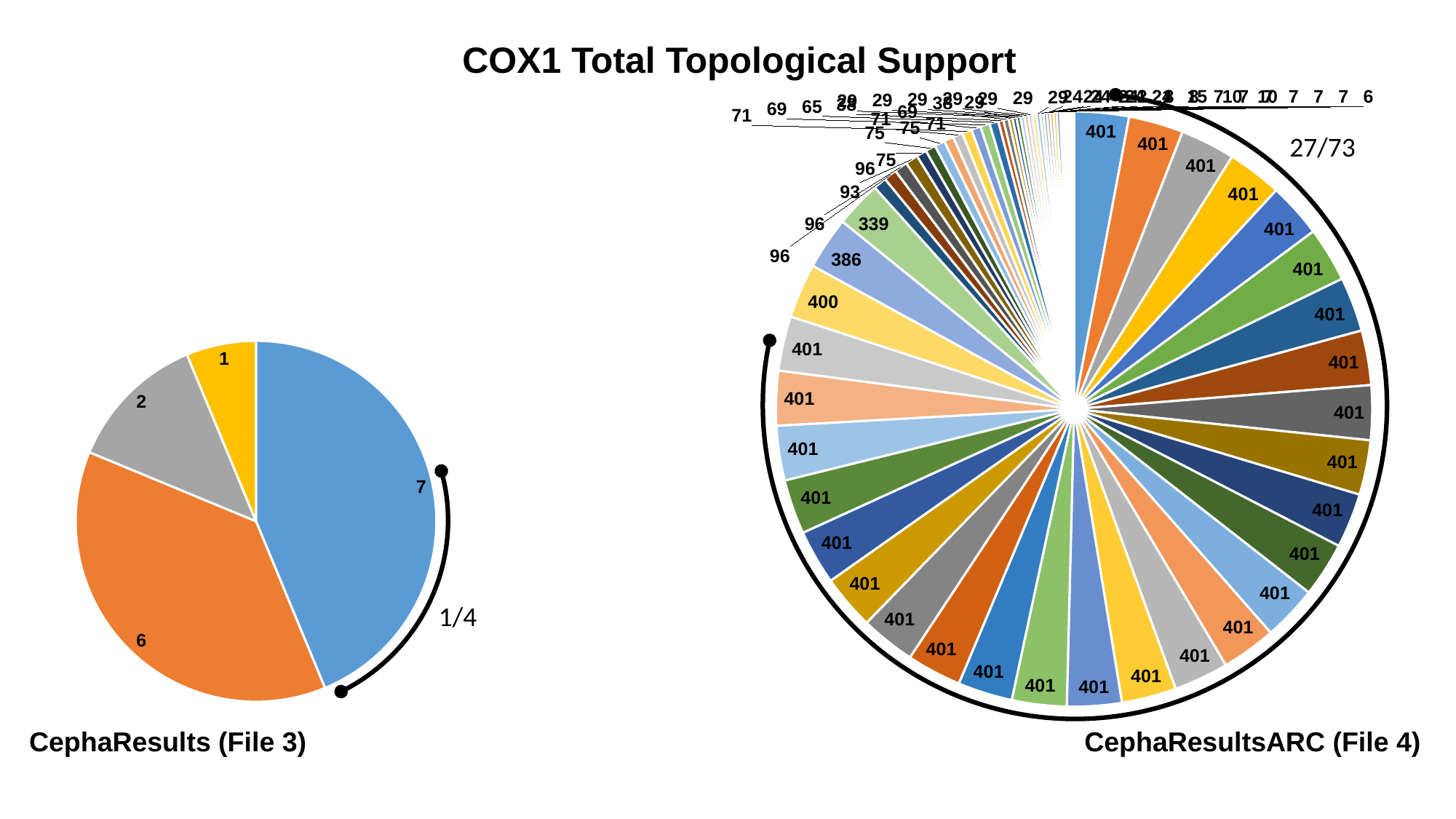

COX1 Total Topological Support
### Chart
| Category | Total Topological Support |
|---|---|
| *** # COX1_KALIGN_GBLOCKSC_DNA_UB # *** | 401.0 |
| *** # COX1_KALIGN_GBLOCKS_DNA_UB # *** | 401.0 |
| *** # COX1_KALIGN_TCSFMw_DNA_UB # *** | 401.0 |
| *** # COX1_KALIGN_TCSFMw_RYt_UB # *** | 401.0 |
| *** # COX1_KALIGN_TCSw_DNA_UB # *** | 401.0 |
| *** # COX1_KALIGN_TCSw_RYt_UB # *** | 401.0 |
| *** # COX1_MAFFTA_PSARALIGN_DNA_UB # *** | 401.0 |
| *** # COX1_MAFFTA_TRIMAL_DNA_UB # *** | 401.0 |
| *** # COX1_MAFFTEI_MAXALIGN_DNA_UB # *** | 401.0 |
| *** # COX1_MAFFTEI_PSARALIGN_DNA_UB # *** | 401.0 |
| *** # COX1_MAFFTEI_TCSf_DNA_UB # *** | 401.0 |
| *** # COX1_MAFFTFI_MAXALIGN_DNA_UB # *** | 401.0 |
| *** # COX1_MAFFTFI_TCSFMf_DNA_UB # *** | 401.0 |
| *** # COX1_MAFFTFI_TRIMALA_DNA_UB # *** | 401.0 |
| *** # COX1_MAFFTGI_MAXALIGN_DNA_UB # *** | 401.0 |
| *** # COX1_MAFFTGI_TCSFMf_DNA_UB # *** | 401.0 |
| *** # COX1_MAFFTGI_TRIMALA_DNA_UB # *** | 401.0 |
| *** # COX1_MAFFTLI_MAXALIGN_DNA_UB # *** | 401.0 |
| *** # COX1_MAFFTLI_TCSFMf_DNA_UB # *** | 401.0 |
| *** # COX1_OPAL_TCSFMw_DEG_UB # *** | 401.0 |
| *** # COX1_PRANKF_TCSFMf_DNA_UB # *** | 401.0 |
| *** # COX1_PRANKF_TRIMALG_DNA_UB # *** | 401.0 |
| *** # COX1_PRANKO_GBLOCKSC_DNA_UB # *** | 401.0 |
| *** # COX1_PRANK_GBLOCKSC_DNA_UB # *** | 401.0 |
| *** # COX1_PRANK_GBLOCKS_DNA_UB # *** | 401.0 |
| *** # COX1_PROBCONS_GBLOCKSC_DNA_UB # *** | 401.0 |
| *** # COX1_TCOFFEEPL_GBLOCKSC_DNA_UB # *** | 401.0 |
| *** # COX1_TCOFFEETC_GBLOCKSC_DNA_UB # *** | 400.0 |
| *** # COX1_TCOFFEE_GBLOCKSC_DNA_UB # *** | 386.0 |
| *** COX1_ALL_TRIMALC_DNA_UB *** | 339.0 |
| *** # COX1_GRAMALIGN_MAXALIGN_RYt_UB # *** | 96.0 |
| *** # COX1_PROBCONS_MAXALIGN_RYt_UB # *** | 96.0 |
| *** # COX1_PROBCONS_TRIMAL_RYt_UB # *** | 96.0 |
| *** # COX1_TCOFFEETC_PSARALIGN_RYt_UB # *** | 93.0 |
| *** # COX1_FSA_GBLOCKS_DEG_UB # *** | 75.0 |
| *** # COX1_GRAMALIGN_TRIMALA_DEG_UB # *** | 75.0 |
| *** # COX1_GRAMALIGN_TRIMALG_DEG_UB # *** | 75.0 |
| *** # COX1_KALIGN_TRIMALS_DNA_UB # *** | 71.0 |
| *** # COX1_MAFFTF2_TRIMALP_DEG_UB # *** | 71.0 |
| *** # COX1_MAFFTGI_TRIMALP_DNA_UB # *** | 71.0 |
| *** # COX1_MAFFT_TCSOGw_DEG_UB # *** | 69.0 |
| *** # COX1_MUSCLE_TCSOGw_RYt_UB # *** | 69.0 |
| *** COX1_ALL_TRIMALC_RYt_UB *** | 65.0 |
| *** # COX1_GRAMALIGN_GBLOCKS_RYt_UB # *** | 38.0 |
| *** # COX1_GRAMALIGN_TRIMALA_RYt_UB # *** | 38.0 |
| *** # COX1_PRANKCDF_TCSFMf_2AA_UB # *** | 29.0 |
| *** # COX1_PRANKCDF_TRIMALA_2AA_UB # *** | 29.0 |
| *** # COX1_PRANKCDO_TRIMALG_2AA_UB # *** | 29.0 |
| *** # COX1_PRANKCDO_TRIMAL_2AA_UB # *** | 29.0 |
| *** # COX1_PRANKCD_GBLOCKSC_2AA_UB # *** | 29.0 |
| *** # COX1_PRANKCD_MAXALIGN_2AA_UB # *** | 29.0 |
| *** # COX1_PRANKCD_PSARALIGN_2AA_UB # *** | 29.0 |
| *** # COX1_PRANKCD_TCSf_2AA_UB # *** | 29.0 |
| *** # COX1_FSA_NOISY_DNA_UB # *** | 24.0 |
| *** # COX1_KALIGN_NOISY_DNA_UB # *** | 24.0 |
| *** # COX1_MAFFTA_NOISY_DNA_UB # *** | 24.0 |
| *** # COX1_MAFFTEI_NOISY_DNA_UB # *** | 24.0 |
| *** # COX1_OPAL_NOISY_DNA_UB # *** | 24.0 |
| *** # COX1_PRANKF_NOISY_DNA_UB # *** | 24.0 |
| *** # COX1_KALIGN_TCSw_DEG_UB # *** | 15.0 |
| *** # COX1_MAFFT_TCSOGw_DNA_UB # *** | 10.0 |
| *** # COX1_TCOFFEE_TRIMALS_RYt_UB # *** | 10.0 |
| *** # COX1_PRANKCDO_MAXALIGN_CDN_UB # *** | 8.0 |
| *** # COX1_PRANKCDO_TCSFMf_CDN_UB # *** | 8.0 |
| *** # COX1_FSA_TRIMALG_RYt_UB # *** | 7.0 |
| *** # COX1_PRANKCDF_TCSf_CDN_UB # *** | 7.0 |
| *** # COX1_PRANKCDF_TRIMALG_CDN_UB # *** | 7.0 |
| *** # COX1_PRANKCDO_GBLOCKSC_CDN_UB # *** | 7.0 |
| *** # COX1_PRANKCDO_TRIMAL_CDN_UB # *** | 7.0 |
| *** # COX1_PRANKCD_TRIMALA_CDN_UB # *** | 7.0 |
| *** # COX1_TCOFFEEPL_TRIMALS_DEG_UB # *** | 6.0 |
| *** # COX1_PRANKCDF_PSARALIGN_CDN_UB # *** | 2.0 |
| *** # COX1_PRANKF_TRIMALP_RYt_UB # *** | 1.0 |
27/73
### Chart
| Category | Total Topological Support |
|---|---|
| *** # COX1_PRANK_DNA_UB # *** | 7.0 |
| *** # COX1_TCOFFEETC_RYt_UB # *** | 6.0 |
| *** # COX1_PRANKCDO_2AA_UB # *** | 2.0 |
| *** # COX1_PRANKCD_CDN_UB # *** | 1.0 |
1/4
CephaResults (File 3)
CephaResultsARC (File 4)

## Slide 4
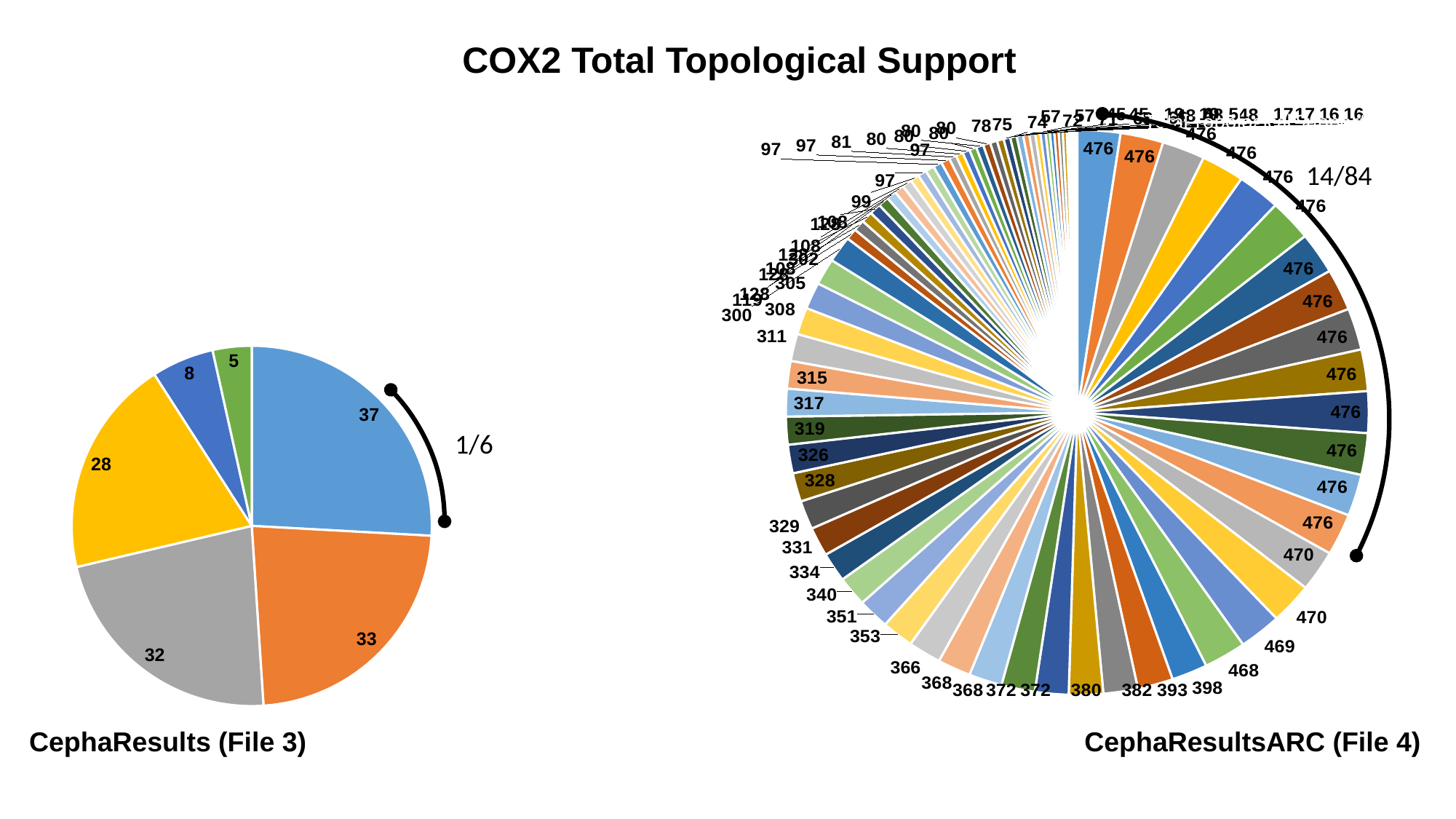

COX2 Total Topological Support
### Chart:
| Category | Total Topological Support |
|---|---|
| *** # COX2_CLUSTALO_TCSFMw_RYt_UB # *** | 476.0 |
| *** # COX2_CLUSTALO_TCSw_DNA_UB # *** | 476.0 |
| *** # COX2_CLUSTALO_TCSw_RYt_UB # *** | 476.0 |
| *** # COX2_CLUSTALW_TCSFMw_RYt_UB # *** | 476.0 |
| *** # COX2_CLUSTALW_TCSw_RYt_UB # *** | 476.0 |
| *** # COX2_MAFFTA_TCSFMw_DNA_UB # *** | 476.0 |
| *** # COX2_MAFFTEI_TCSFMw_DEG_UB # *** | 476.0 |
| *** # COX2_MAFFTF1_TCSFMw_DEG_UB # *** | 476.0 |
| *** # COX2_MAFFTF1_TCSw_DEG_UB # *** | 476.0 |
| *** # COX2_MAFFTF2_TCSFMw_DNA_UB # *** | 476.0 |
| *** # COX2_MAFFTFI_TRIMALP_DEG_UB # *** | 476.0 |
| *** # COX2_MAFFT_TCSw_DNA_UB # *** | 476.0 |
| *** # COX2_PRANKCDF_GBLOCKSC_DNA_UB # *** | 476.0 |
| *** # COX2_PRANKCDO_GBLOCKSC_DNA_UB # *** | 476.0 |
| *** COX2_ALL_TRIMALC_DNA_UB *** | 470.0 |
| *** COX2_ALL_WEAVEALIGN_DNA_UB *** | 470.0 |
| *** # COX2_PRANKCDO_TRIMALA_DNA_UB # *** | 469.0 |
| *** # COX2_PRANKCDO_TRIMALG_DNA_UB # *** | 468.0 |
| *** # COX2_PROBCONS_PSARALIGN_DNA_UB # *** | 398.0 |
| *** # COX2_PROBCONS_TCSf_DNA_UB # *** | 393.0 |
| *** # COX2_TCOFFEEPL_MAXALIGN_DNA_UB # *** | 382.0 |
| *** # COX2_TCOFFEEPL_TCSFMf_DNA_UB # *** | 380.0 |
| *** # COX2_PRANKCDO_GBLOCKSC_2AA_UB # *** | 372.0 |
| *** # COX2_PRANKCDO_GBLOCKS_2AA_UB # *** | 372.0 |
| *** # COX2_PRANKCDO_TRIMALA_2AA_UB # *** | 368.0 |
| *** # COX2_TCOFFEEPL_TRIMAL_DNA_UB # *** | 368.0 |
| *** # COX2_PRANKCDO_TRIMALG_2AA_UB # *** | 366.0 |
| *** # COX2_PRANKCD_TRIMALA_CDN_UB # *** | 353.0 |
| *** # COX2_PRANKCD_TRIMALG_CDN_UB # *** | 351.0 |
| *** # COX2_PROBCONS_TCSf_CDN_UB # *** | 340.0 |
| *** # COX2_PROBCONS_TRIMAL_CDN_UB # *** | 334.0 |
| *** # COX2_TCOFFEEPL_MAXALIGN_CDN_UB # *** | 331.0 |
| *** # COX2_TCOFFEEPL_PSARALIGN_CDN_UB # *** | 329.0 |
| *** # COX2_TCOFFEEPL_PSARALIGN_2AA_UB # *** | 328.0 |
| *** # COX2_TCOFFEEPL_TCSFMf_2AA_UB # *** | 326.0 |
| *** # COX2_TCOFFEETC_MAXALIGN_2AA_UB # *** | 319.0 |
| *** # COX2_TCOFFEETC_PSARALIGN_2AA_UB # *** | 317.0 |
| *** # COX2_TCOFFEETC_TCSFMf_2AA_UB # *** | 315.0 |
| *** # COX2_TCOFFEETC_TRIMAL_2AA_UB # *** | 311.0 |
| *** # COX2_TCOFFEE_MAXALIGN_2AA_UB # *** | 308.0 |
| *** # COX2_TCOFFEE_TCSFMf_CDN_UB # *** | 305.0 |
| *** # COX2_TCOFFEE_TCSf_2AA_UB # *** | 302.0 |
| *** # COX2_TCOFFEE_TRIMAL_2AA_UB # *** | 300.0 |
| *** # COX2_CLUSTALW_TRIMALS_RYt_UB # *** | 128.0 |
| *** # COX2_GRAMALIGN_TRIMALP_DNA_UB # *** | 128.0 |
| *** # COX2_MAFFTGI_TRIMALS_DEG_UB # *** | 128.0 |
| *** # COX2_MAFFTGI_TRIMALS_DNA_UB # *** | 128.0 |
| *** # COX2_CLUSTALW_GBLOCKS_DEG_UB # *** | 119.0 |
| *** COX2_ALL_MERGEALIGN_DEG_UB *** | 108.0 |
| *** COX2_ALL_TRIMALC_DEG_UB *** | 108.0 |
| *** COX2_ALL_WEAVEALIGN_DEG_UB *** | 108.0 |
| *** # COX2_MAFFTEI_GBLOCKS_DNA_UB # *** | 99.0 |
| *** # COX2_FSA_GBLOCKSC_DEG_UB # *** | 97.0 |
| *** # COX2_KALIGN_TCSFMf_DEG_UB # *** | 97.0 |
| *** # COX2_KALIGN_TRIMAL_DEG_UB # *** | 97.0 |
| *** # COX2_MUSCLE_TRIMALA_DEG_UB # *** | 97.0 |
| *** # COX2_PROBCONS_MAXALIGN_DEG_UB # *** | 81.0 |
| *** # COX2_PROBCONS_PSARALIGN_DEG_UB # *** | 80.0 |
| *** COX2_ALL_TRIMALC_2AA_UB *** | 80.0 |
| *** COX2_ALL_TRIMALC_CDN_UB *** | 80.0 |
| *** COX2_ALL_WEAVEALIGN_2AA_UB *** | 80.0 |
| *** COX2_ALL_WEAVEALIGN_CDN_UB *** | 80.0 |
| *** # COX2_PROBCONS_TCSf_DEG_UB # *** | 78.0 |
| *** # COX2_PROBCONS_TRIMAL_DEG_UB # *** | 75.0 |
| *** # COX2_TCOFFEEPL_MAXALIGN_RYt_UB # *** | 74.0 |
| *** # COX2_PRANKCD_GBLOCKSC_RYt_UB # *** | 72.0 |
| *** # COX2_TCOFFEEPL_TCSf_RYt_UB # *** | 71.0 |
| *** # COX2_TCOFFEEPL_TRIMAL_RYt_UB # *** | 68.0 |
| *** # COX2_TCOFFEETC_TCSFMf_RYt_UB # *** | 66.0 |
| *** # COX2_KALIGN_TCSOGw_DEG_UB # *** | 57.0 |
| *** # COX2_TCOFFEE_PSARALIGN_RYt_UB # *** | 57.0 |
| *** # COX2_PRANKCDF_GBLOCKSC_CDN_UB # *** | 48.0 |
| *** # COX2_PRANKCDF_GBLOCKS_CDN_UB # *** | 48.0 |
| *** # COX2_TCOFFEEPL_TCSOGw_RYt_UB # *** | 48.0 |
| *** # COX2_MAFFTA_NOISY_DEG_UB # *** | 45.0 |
| *** # COX2_MAFFTF1_NOISY_DNA_UB # *** | 45.0 |
| *** COX2_ALL_TRIMALC_RYt_UB *** | 19.0 |
| *** COX2_ALL_WEAVEALIGN_RYt_UB *** | 19.0 |
| *** # COX2_CLUSTALO_NOISY_RYt_UB # *** | 17.0 |
| *** # COX2_CLUSTALW_NOISY_RYt_UB # *** | 17.0 |
| *** # COX2_PRANKCD_TRIMALG_DEG_UB # *** | 16.0 |
| *** # COX2_TCOFFEE_TCSOGw_DNA_UB # *** | 16.0 |
| *** # COX2_PRANKCD_TRIMALA_RYt_UB # *** | 6.0 |
| *** # COX2_PRANKCDO_TRIMALG_RYt_UB # *** | 5.0 |
14/84
### Chart
| Category | Total Topological Support |
|---|---|
| *** # COX2_FSANP_DNA_UB # *** | 37.0 |
| *** # COX2_TCOFFEEPL_CDN_UB # *** | 33.0 |
| *** # COX2_TCOFFEEPL_2AA_UB # *** | 32.0 |
| *** # COX2_TCOFFEE_2AA_UB # *** | 28.0 |
| *** # COX2_PROBCONS_DEG_UB # *** | 8.0 |
| *** # COX2_TCOFFEE_RYt_UB # *** | 5.0 |
1/6
CephaResults (File 3)
CephaResultsARC (File 4)

## Slide 5
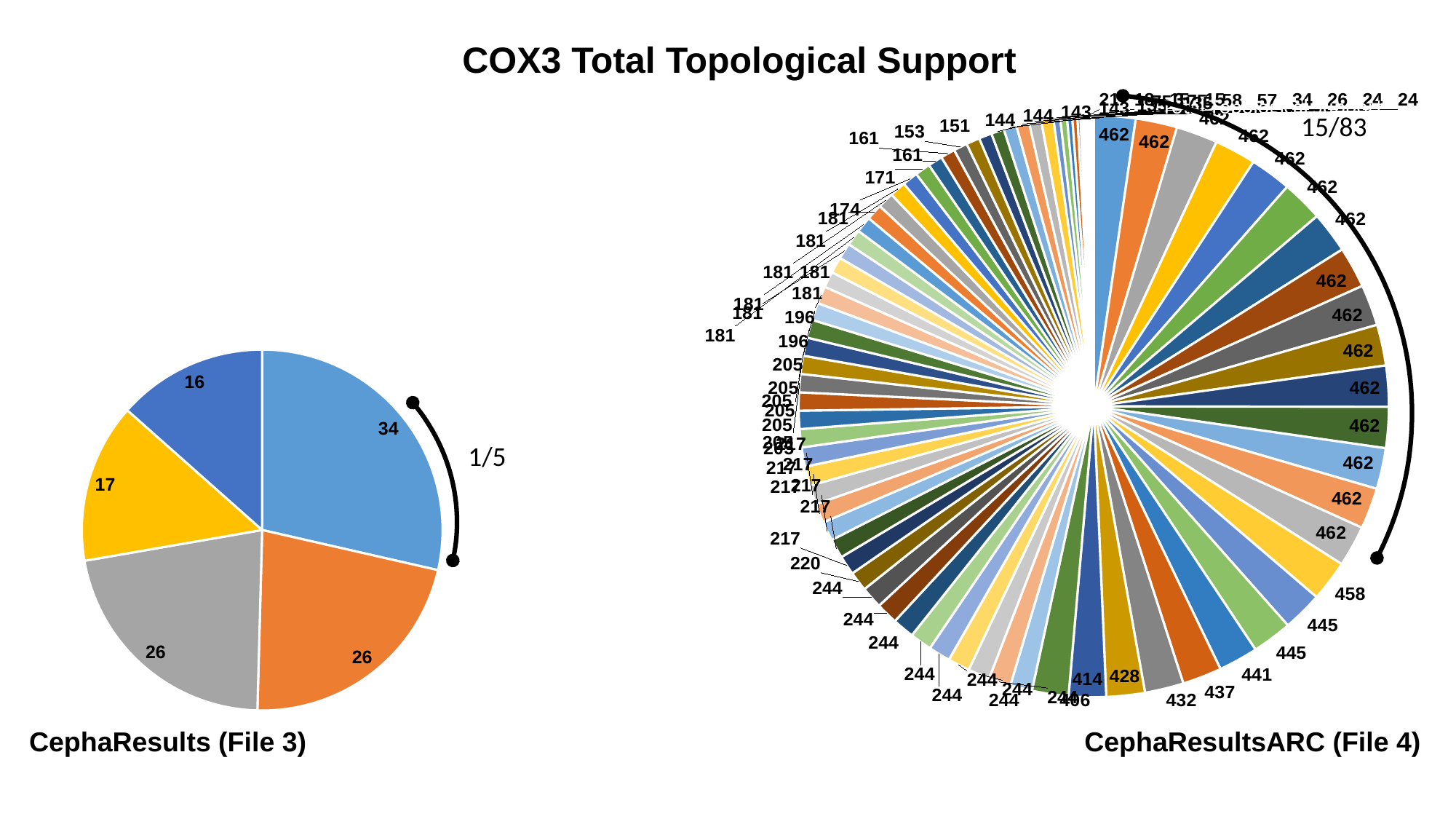

COX3 Total Topological Support
### Chart:
| Category | Total Topological Support |
|---|---|
| *** # COX3_CLUSTALW_MAXALIGN_DNA_UB # *** | 462.0 |
| *** # COX3_CLUSTALW_TCSFMf_DNA_UB # *** | 462.0 |
| *** # COX3_CLUSTALW_TCSf_DNA_UB # *** | 462.0 |
| *** # COX3_CLUSTALW_TRIMAL_DNA_UB # *** | 462.0 |
| *** # COX3_FSANP_TCSFMw_RYt_UB # *** | 462.0 |
| *** # COX3_FSA_TCSw_RYt_UB # *** | 462.0 |
| *** # COX3_GRAMALIGN_GBLOCKSC_DNA_UB # *** | 462.0 |
| *** # COX3_MACSE_GBLOCKS_DNA_UB # *** | 462.0 |
| *** # COX3_MAFFTA_MAXALIGN_DNA_UB # *** | 462.0 |
| *** # COX3_MAFFTF1_TCSw_DNA_UB # *** | 462.0 |
| *** # COX3_MAFFTFI_GBLOCKS_DNA_UB # *** | 462.0 |
| *** # COX3_MAFFTFI_TCSFMf_DNA_UB # *** | 462.0 |
| *** # COX3_MAFFTLI_TCSFMf_DNA_UB # *** | 462.0 |
| *** # COX3_OPAL_TCSw_RYt_UB # *** | 462.0 |
| *** # COX3_PRANKO_TRIMALA_DNA_UB # *** | 462.0 |
| *** # COX3_PRANK_MAXALIGN_DNA_UB # *** | 458.0 |
| *** COX3_ALL_TRIMALC_DNA_UB *** | 445.0 |
| *** COX3_ALL_WEAVEALIGN_DNA_UB *** | 445.0 |
| *** # COX3_PROBCONS_PSARALIGN_DNA_UB # *** | 441.0 |
| *** # COX3_PROBCONS_TCSf_DNA_UB # *** | 437.0 |
| *** # COX3_PROBCONS_TRIMALG_DNA_UB # *** | 432.0 |
| *** # COX3_TCOFFEEPL_MAXALIGN_DNA_UB # *** | 428.0 |
| *** # COX3_TCOFFEETC_GBLOCKS_DNA_UB # *** | 414.0 |
| *** # COX3_TCOFFEETC_TCSw_DEG_UB # *** | 406.0 |
| *** # COX3_FSANP_MAXALIGN_DEG_UB # *** | 244.0 |
| *** # COX3_FSANP_TRIMALG_DEG_UB # *** | 244.0 |
| *** # COX3_FSA_TCSf_DEG_UB # *** | 244.0 |
| *** # COX3_KALIGN_TRIMAL_DEG_UB # *** | 244.0 |
| *** # COX3_MAFFTA_GBLOCKS_DEG_UB # *** | 244.0 |
| *** # COX3_MAFFTA_TRIMALA_DEG_UB # *** | 244.0 |
| *** # COX3_MAFFTF1_TCSf_DEG_UB # *** | 244.0 |
| *** # COX3_MAFFTLI_PSARALIGN_DEG_UB # *** | 244.0 |
| *** # COX3_PRANKO_GBLOCKSC_DEG_UB # *** | 244.0 |
| *** # COX3_TCOFFEEPL_TCSFMf_DEG_UB # *** | 220.0 |
| *** # COX3_MACSE_GBLOCKSC_CDN_UB # *** | 217.0 |
| *** # COX3_MACSE_TRIMALG_CDN_UB # *** | 217.0 |
| *** # COX3_MAFFTA_TCSFMf_CDN_UB # *** | 217.0 |
| *** # COX3_MAFFTA_TRIMALA_CDN_UB # *** | 217.0 |
| *** # COX3_MAFFTGI_MAXALIGN_CDN_UB # *** | 217.0 |
| *** # COX3_MUSCLE_PSARALIGN_CDN_UB # *** | 217.0 |
| *** # COX3_OPAL_TRIMAL_CDN_UB # *** | 217.0 |
| *** # COX3_FSA_TRIMALG_RYt_UB # *** | 205.0 |
| *** # COX3_MACSE_MAXALIGN_RYt_UB # *** | 205.0 |
| *** # COX3_MACSE_TCSFMf_RYt_UB # *** | 205.0 |
| *** # COX3_MUSCLE_GBLOCKSC_RYt_UB # *** | 205.0 |
| *** # COX3_MUSCLE_GBLOCKS_RYt_UB # *** | 205.0 |
| *** # COX3_MUSCLE_TRIMAL_RYt_UB # *** | 205.0 |
| *** # COX3_PRANKF_PSARALIGN_RYt_UB # *** | 205.0 |
| *** COX3_ALL_TRIMALC_DEG_UB *** | 196.0 |
| *** COX3_ALL_WEAVEALIGN_DEG_UB *** | 196.0 |
| *** # COX3_GRAMALIGN_GBLOCKSC_2AA_UB # *** | 181.0 |
| *** # COX3_MACSE_TRIMALA_2AA_UB # *** | 181.0 |
| *** # COX3_MAFFTF1_MAXALIGN_2AA_UB # *** | 181.0 |
| *** # COX3_MAFFTF1_TCSFMf_2AA_UB # *** | 181.0 |
| *** # COX3_MAFFTFI_MAXALIGN_2AA_UB # *** | 181.0 |
| *** # COX3_MAFFTFI_TCSf_2AA_UB # *** | 181.0 |
| *** # COX3_PRANKF_TRIMALG_2AA_UB # *** | 181.0 |
| *** # COX3_PRANKO_GBLOCKSC_2AA_UB # *** | 181.0 |
| *** # COX3_PRANK_TRIMALG_2AA_UB # *** | 174.0 |
| *** # COX3_PROBCONS_PSARALIGN_2AA_UB # *** | 171.0 |
| *** # COX3_TCOFFEEPL_TRIMALA_2AA_UB # *** | 161.0 |
| *** # COX3_TCOFFEE_TCSf_RYt_UB # *** | 161.0 |
| *** # COX3_TCOFFEETC_TRIMAL_2AA_UB # *** | 153.0 |
| *** # COX3_TCOFFEE_MAXALIGN_2AA_UB # *** | 151.0 |
| *** COX3_ALL_TRIMALC_CDN_UB *** | 144.0 |
| *** COX3_ALL_WEAVEALIGN_CDN_UB *** | 144.0 |
| *** COX3_ALL_TRIMALC_RYt_UB *** | 143.0 |
| *** COX3_ALL_WEAVEALIGN_RYt_UB *** | 143.0 |
| *** COX3_ALL_TRIMALC_2AA_UB *** | 135.0 |
| *** COX3_ALL_WEAVEALIGN_2AA_UB *** | 135.0 |
| *** # COX3_CLUSTALO_TCSOGw_RYt_UB # *** | 75.0 |
| *** # COX3_MAFFTGI_TCSFMw_DNA_UB # *** | 75.0 |
| *** # COX3_MAFFTGI_TRIMALS_DNA_UB # *** | 58.0 |
| *** # COX3_PRANK_TRIMALP_DNA_UB # *** | 57.0 |
| *** # COX3_MAFFTF1_TCSOGw_DNA_UB # *** | 34.0 |
| *** # COX3_CLUSTALO_NOISY_DNA_UB # *** | 26.0 |
| *** # COX3_PROBCONS_TRIMALS_DEG_UB # *** | 24.0 |
| *** # COX3_TCOFFEEPL_TCSOGw_DEG_UB # *** | 24.0 |
| *** # COX3_TCOFFEE_TRIMALP_DEG_UB # *** | 21.0 |
| *** # COX3_PRANKO_TRIMALP_RYt_UB # *** | 18.0 |
| *** # COX3_MAFFTF1_NOISY_DEG_UB # *** | 15.0 |
| *** # COX3_TCOFFEEPL_TRIMALS_RYt_UB # *** | 15.0 |
| *** # COX3_MAFFTEI_NOISY_RYt_UB # *** | 3.0 |
15/83
### Chart
| Category | Total Topological Support |
|---|---|
| *** # COX3_MAFFTF1_DNA_UB # *** | 34.0 |
| *** # COX3_GRAMALIGN_DEG_UB # *** | 26.0 |
| *** # COX3_MAFFTLI_CDN_UB # *** | 26.0 |
| *** # COX3_PROBCONS_2AA_UB # *** | 17.0 |
| *** # COX3_PRANKF_RYt_UB # *** | 16.0 |
1/5
CephaResults (File 3)
CephaResultsARC (File 4)

## Slide 6
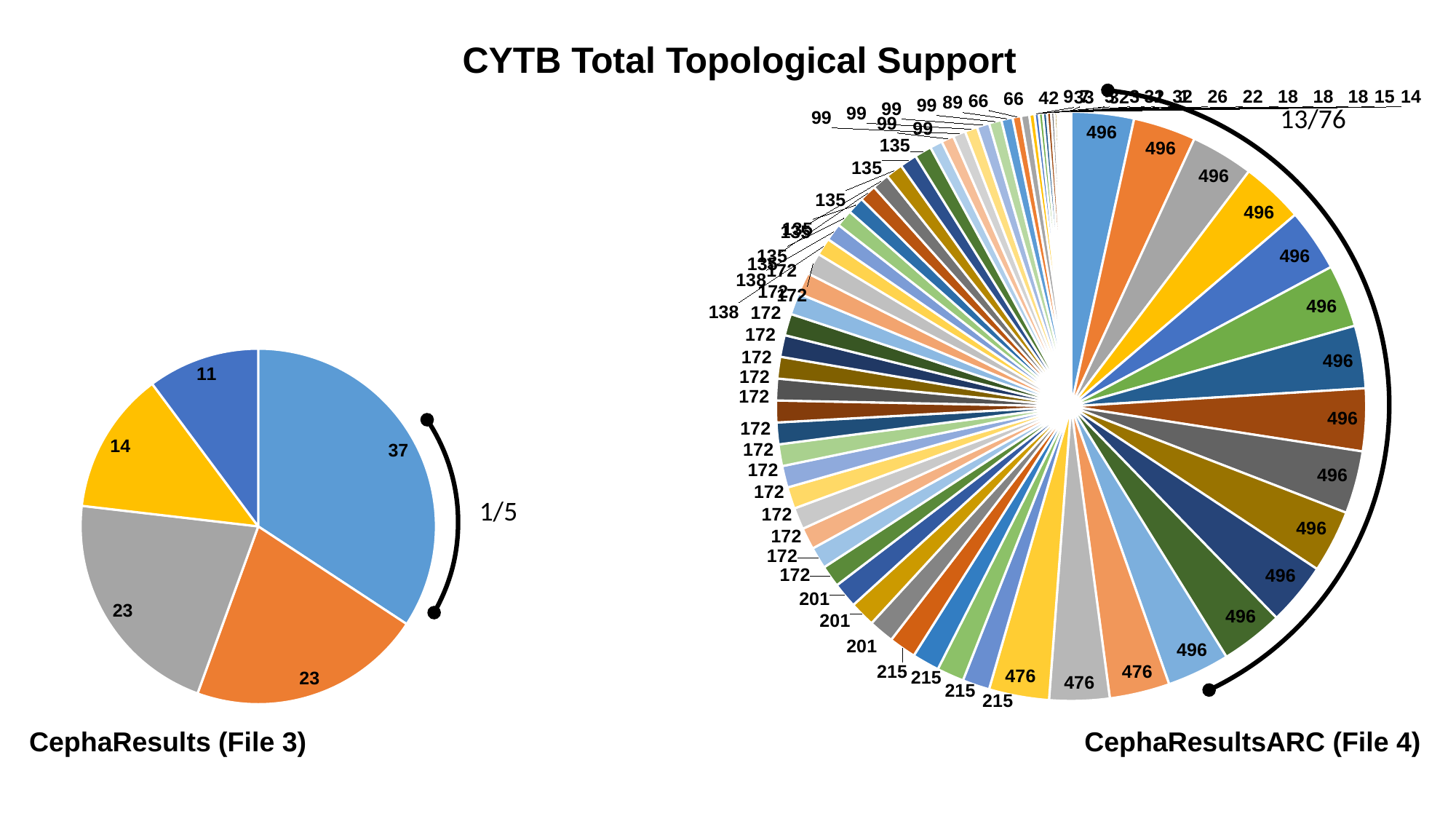

CYTB Total Topological Support
### Chart
| Category | Total Topological Support |
|---|---|
| *** # CYTB_CLUSTALW_PSARALIGN_DNA_UB # *** | 496.0 |
| *** # CYTB_KALIGN_MAXALIGN_DNA_UB # *** | 496.0 |
| *** # CYTB_KALIGN_TCSFMf_DNA_UB # *** | 496.0 |
| *** # CYTB_KALIGN_TCSf_DNA_UB # *** | 496.0 |
| *** # CYTB_KALIGN_TRIMAL_DNA_UB # *** | 496.0 |
| *** # CYTB_MAFFTGI_GBLOCKS_DNA_UB # *** | 496.0 |
| *** # CYTB_MAFFTGI_TRIMALA_DNA_UB # *** | 496.0 |
| *** # CYTB_MAFFTGI_TRIMALG_DNA_UB # *** | 496.0 |
| *** # CYTB_MAFFT_GBLOCKSC_DNA_UB # *** | 496.0 |
| *** # CYTB_MAFFT_TCSFMw_DNA_UB # *** | 496.0 |
| *** # CYTB_MUSCLE_TCSOGw_DEG_UB # *** | 496.0 |
| *** # CYTB_OPAL_TCSFMw_RYt_UB # *** | 496.0 |
| *** # CYTB_OPAL_TCSw_RYt_UB # *** | 496.0 |
| *** CYTB_ALL_TRIMALC_DNA_UB *** | 476.0 |
| *** CYTB_ALL_TRIMALC_RYt_UB *** | 476.0 |
| *** CYTB_ALL_WEAVEALIGN_DNA_UB *** | 476.0 |
| *** # CYTB_CLUSTALW_PSARALIGN_DEG_UB # *** | 215.0 |
| *** # CYTB_KALIGN_MAXALIGN_DEG_UB # *** | 215.0 |
| *** # CYTB_KALIGN_TCSf_DEG_UB # *** | 215.0 |
| *** # CYTB_OPAL_GBLOCKSC_DEG_UB # *** | 215.0 |
| *** CYTB_ALL_MERGEALIGN_DEG_UB *** | 201.0 |
| *** CYTB_ALL_TRIMALC_DEG_UB *** | 201.0 |
| *** CYTB_ALL_WEAVEALIGN_DEG_UB *** | 201.0 |
| *** # CYTB_FSANP_GBLOCKS_2AA_UB # *** | 172.0 |
| *** # CYTB_FSANP_MAXALIGN_2AA_UB # *** | 172.0 |
| *** # CYTB_FSANP_TCSf_2AA_UB # *** | 172.0 |
| *** # CYTB_FSANP_TRIMAL_2AA_UB # *** | 172.0 |
| *** # CYTB_MACSE_TCSFMf_2AA_UB # *** | 172.0 |
| *** # CYTB_MACSE_TCSf_2AA_UB # *** | 172.0 |
| *** # CYTB_MACSE_TRIMAL_2AA_UB # *** | 172.0 |
| *** # CYTB_MAFFTF1_TRIMALA_2AA_UB # *** | 172.0 |
| *** # CYTB_MAFFTF2_GBLOCKSC_2AA_UB # *** | 172.0 |
| *** # CYTB_MAFFTF2_TRIMALA_2AA_UB # *** | 172.0 |
| *** # CYTB_MAFFTF2_TRIMALG_2AA_UB # *** | 172.0 |
| *** # CYTB_MAFFTGI_GBLOCKS_DEG_UB # *** | 172.0 |
| *** # CYTB_MAFFT_TRIMALA_2AA_UB # *** | 172.0 |
| *** # CYTB_MAFFT_TRIMALG_2AA_UB # *** | 172.0 |
| *** # CYTB_OPAL_TRIMALA_2AA_UB # *** | 172.0 |
| *** # CYTB_PRANKCD_PSARALIGN_2AA_UB # *** | 172.0 |
| *** CYTB_ALL_TRIMALC_2AA_UB *** | 138.0 |
| *** CYTB_ALL_WEAVEALIGN_2AA_UB *** | 138.0 |
| *** # CYTB_GRAMALIGN_GBLOCKSC_RYt_UB # *** | 135.0 |
| *** # CYTB_KALIGN_MAXALIGN_RYt_UB # *** | 135.0 |
| *** # CYTB_KALIGN_TCSFMf_RYt_UB # *** | 135.0 |
| *** # CYTB_KALIGN_TCSf_RYt_UB # *** | 135.0 |
| *** # CYTB_KALIGN_TRIMAL_RYt_UB # *** | 135.0 |
| *** # CYTB_MAFFTF1_TCSw_DEG_UB # *** | 135.0 |
| *** # CYTB_MAFFT_TCSFMw_DEG_UB # *** | 135.0 |
| *** # CYTB_KALIGN_MAXALIGN_CDN_UB # *** | 99.0 |
| *** # CYTB_KALIGN_TRIMAL_CDN_UB # *** | 99.0 |
| *** # CYTB_MAFFTF1_TCSf_CDN_UB # *** | 99.0 |
| *** # CYTB_MAFFTGI_TRIMALA_CDN_UB # *** | 99.0 |
| *** # CYTB_MAFFTGI_TRIMALG_CDN_UB # *** | 99.0 |
| *** # CYTB_MAFFTLI_TCSFMf_CDN_UB # *** | 99.0 |
| *** CYTB_ALL_WEAVEALIGN_RYt_UB *** | 89.0 |
| *** CYTB_ALL_TRIMALC_CDN_UB *** | 66.0 |
| *** CYTB_ALL_WEAVEALIGN_CDN_UB *** | 66.0 |
| *** # CYTB_OPAL_TRIMALS_DNA_UB # *** | 42.0 |
| *** # CYTB_CLUSTALW_PSARALIGN_RYt_UB # *** | 33.0 |
| *** # CYTB_CLUSTALO_GBLOCKS_CDN_UB # *** | 32.0 |
| *** # CYTB_MAFFTFI_GBLOCKSC_CDN_UB # *** | 32.0 |
| *** # CYTB_PRANKCD_PSARALIGN_CDN_UB # *** | 32.0 |
| *** # CYTB_MAFFTF2_TRIMALS_DEG_UB # *** | 26.0 |
| *** # CYTB_MAFFTF1_TRIMALP_DNA_UB # *** | 22.0 |
| *** # CYTB_MAFFTF2_TRIMALP_DEG_UB # *** | 18.0 |
| *** # CYTB_OPAL_TRIMALA_RYt_UB # *** | 18.0 |
| *** # CYTB_OPAL_TRIMALG_RYt_UB # *** | 18.0 |
| *** # CYTB_OPAL_GBLOCKS_RYt_UB # *** | 15.0 |
| *** # CYTB_GRAMALIGN_TRIMALP_RYt_UB # *** | 14.0 |
| *** # CYTB_PROBCONS_NOISY_DNA_UB # *** | 9.0 |
| *** # CYTB_GRAMALIGN_TRIMALS_RYt_UB # *** | 7.0 |
| *** # CYTB_MUSCLE_TCSOGw_RYt_UB # *** | 5.0 |
| *** # CYTB_CLUSTALO_NOISY_DEG_UB # *** | 3.0 |
| *** # CYTB_MAFFTGI_TRIMALA_DEG_UB # *** | 1.0 |
| *** # CYTB_MAFFTGI_TRIMALG_DEG_UB # *** | 1.0 |
| # CYTB_CLUSTALO_NOISY_RYt_UB # | 0.0 |
13/76
### Chart
| Category | Total Topological Support |
|---|---|
| *** # CYTB_KALIGN_DNA_UB # *** | 37.0 |
| *** # CYTB_FSANP_2AA_UB # *** | 23.0 |
| *** # CYTB_MACSE_2AA_UB # *** | 23.0 |
| *** # CYTB_CLUSTALO_CDN_UB # *** | 14.0 |
| *** # CYTB_KALIGN_RYt_UB # *** | 11.0 |
1/5
CephaResults (File 3)
CephaResultsARC (File 4)

## Slide 7
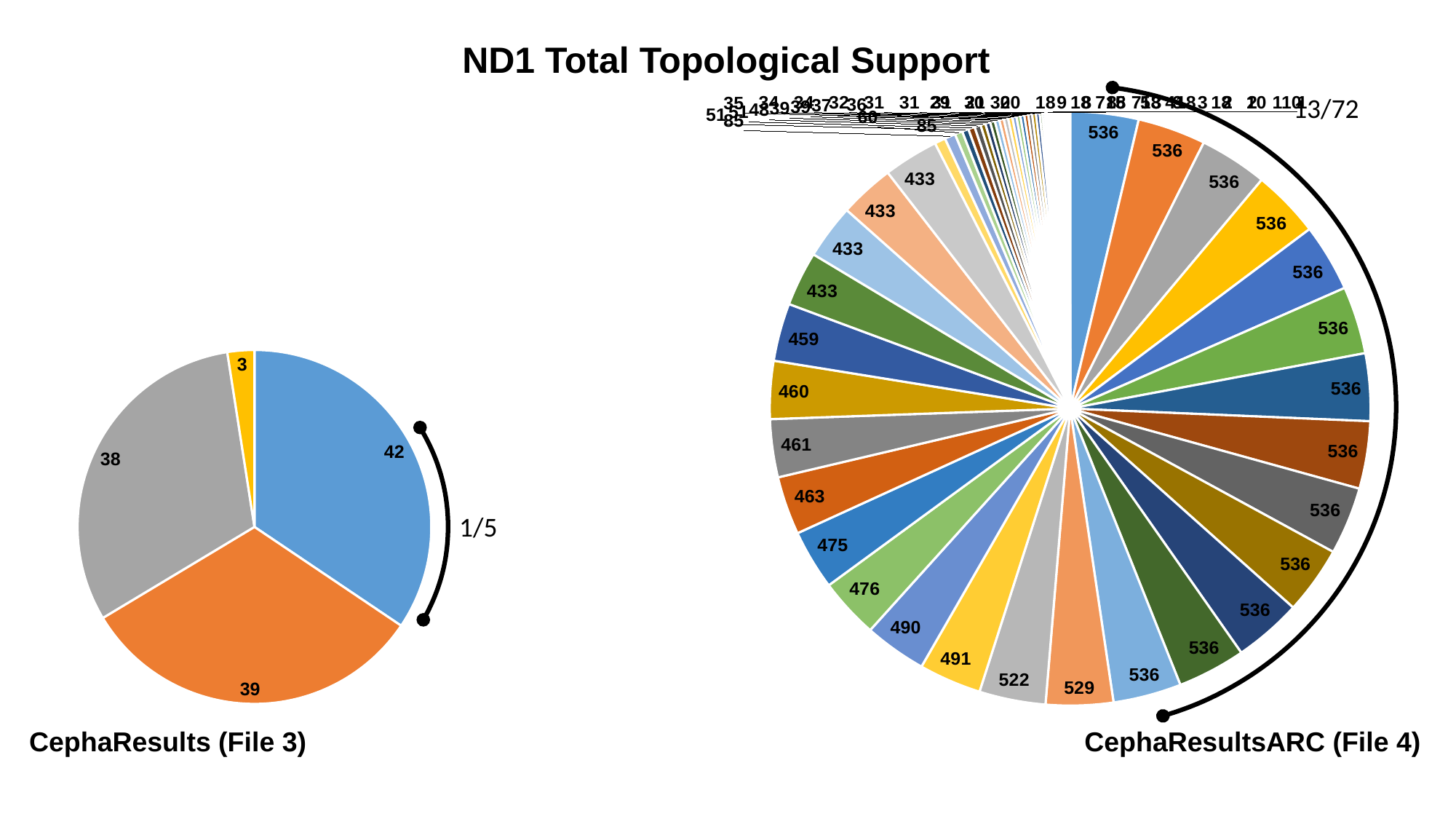

ND1 Total Topological Support
### Chart
| Category | Total Topological Support |
|---|---|
| *** # ND1_FSANP_TRIMALA_DNA_UB # *** | 536.0 |
| *** # ND1_FSANP_TRIMALG_DNA_UB # *** | 536.0 |
| *** # ND1_MAFFTEI_TCSFMf_DEG_UB # *** | 536.0 |
| *** # ND1_MAFFTF2_GBLOCKSC_DNA_UB # *** | 536.0 |
| *** # ND1_OPAL_MAXALIGN_DNA_UB # *** | 536.0 |
| *** # ND1_OPAL_MAXALIGN_RYt_UB # *** | 536.0 |
| *** # ND1_OPAL_TCSFMf_DNA_UB # *** | 536.0 |
| *** # ND1_OPAL_TCSFMf_RYt_UB # *** | 536.0 |
| *** # ND1_OPAL_TCSOGw_DNA_UB # *** | 536.0 |
| *** # ND1_OPAL_TCSf_DNA_UB # *** | 536.0 |
| *** # ND1_OPAL_TCSf_RYt_UB # *** | 536.0 |
| *** # ND1_OPAL_TRIMAL_DNA_UB # *** | 536.0 |
| *** # ND1_OPAL_TRIMAL_RYt_UB # *** | 536.0 |
| *** # ND1_PRANKCD_TCSw_RYt_UB # *** | 529.0 |
| *** # ND1_PROBCONS_GBLOCKSC_RYt_UB # *** | 522.0 |
| *** # ND1_TCOFFEEPL_TCSFMw_DNA_UB # *** | 491.0 |
| *** # ND1_TCOFFEEPL_TCSFMw_RYt_UB # *** | 490.0 |
| *** # ND1_TCOFFEETC_TCSFMw_DNA_UB # *** | 476.0 |
| *** # ND1_TCOFFEETC_TCSFMw_RYt_UB # *** | 475.0 |
| *** # ND1_TCOFFEE_PSARALIGN_DNA_UB # *** | 463.0 |
| *** # ND1_TCOFFEE_TCSFMw_DEG_UB # *** | 461.0 |
| *** # ND1_TCOFFEE_TCSFMw_DNA_UB # *** | 460.0 |
| *** # ND1_TCOFFEE_TCSFMw_RYt_UB # *** | 459.0 |
| *** ND1_ALL_TRIMALC_DNA_UB *** | 433.0 |
| *** ND1_ALL_TRIMALC_RYt_UB *** | 433.0 |
| *** ND1_ALL_WEAVEALIGN_DNA_UB *** | 433.0 |
| *** ND1_ALL_WEAVEALIGN_RYt_UB *** | 433.0 |
| *** # ND1_FSANP_TRIMALA_RYt_UB # *** | 85.0 |
| *** # ND1_FSANP_TRIMALG_RYt_UB # *** | 85.0 |
| *** # ND1_PRANKCDO_TCSw_DEG_UB # *** | 60.0 |
| *** # ND1_FSANP_TRIMALA_DEG_UB # *** | 51.0 |
| *** # ND1_FSANP_TRIMALG_DEG_UB # *** | 51.0 |
| *** ND1_ALL_MERGEALIGN_DEG_UB *** | 48.0 |
| *** # ND1_MAFFT_GBLOCKSC_DEG_UB # *** | 39.0 |
| *** # ND1_PRANKO_TCSw_DNA_UB # *** | 39.0 |
| *** # ND1_OPAL_PSARALIGN_CDN_UB # *** | 37.0 |
| *** # ND1_TCOFFEEPL_GBLOCKS_DEG_UB # *** | 36.0 |
| *** # ND1_TCOFFEETC_GBLOCKS_DEG_UB # *** | 35.0 |
| *** # ND1_OPAL_PSARALIGN_2AA_UB # *** | 34.0 |
| *** # ND1_TCOFFEE_GBLOCKS_DEG_UB # *** | 34.0 |
| *** # ND1_TCOFFEEPL_PSARALIGN_RYt_UB # *** | 32.0 |
| *** # ND1_MAFFTEI_MAXALIGN_DEG_UB # *** | 31.0 |
| *** # ND1_MAFFTEI_TRIMAL_DEG_UB # *** | 31.0 |
| *** # ND1_OPAL_TCSf_DEG_UB # *** | 31.0 |
| *** # ND1_FSANP_TRIMALA_CDN_UB # *** | 30.0 |
| *** # ND1_PRANKCDO_TCSFMf_CDN_UB # *** | 30.0 |
| *** # ND1_PRANKCDO_TCSf_CDN_UB # *** | 29.0 |
| *** # ND1_MAFFTF1_TRIMALP_DNA_UB # *** | 21.0 |
| *** # ND1_PRANKCD_TRIMAL_CDN_UB # *** | 20.0 |
| *** # ND1_CLUSTALO_TRIMALS_DEG_UB # *** | 18.0 |
| *** # ND1_CLUSTALO_TRIMALS_RYt_UB # *** | 18.0 |
| *** # ND1_KALIGN_TRIMALP_RYt_UB # *** | 18.0 |
| *** # ND1_MAFFTA_TRIMALP_DEG_UB # *** | 18.0 |
| *** ND1_ALL_TRIMALC_DEG_UB *** | 18.0 |
| *** ND1_ALL_WEAVEALIGN_DEG_UB *** | 18.0 |
| *** # ND1_PRANKCDO_TCSf_2AA_UB # *** | 10.0 |
| *** # ND1_PRANKF_TRIMALS_DNA_UB # *** | 10.0 |
| *** # ND1_MAFFTGI_TCSOGw_DEG_UB # *** | 9.0 |
| *** # ND1_FSANP_TRIMALA_2AA_UB # *** | 8.0 |
| *** # ND1_FSANP_TRIMALG_2AA_UB # *** | 8.0 |
| *** # ND1_OPAL_TCSOGw_RYt_UB # *** | 7.0 |
| *** # ND1_PRANKCDO_TRIMAL_2AA_UB # *** | 7.0 |
| *** # ND1_PRANKCDO_GBLOCKSC_CDN_UB # *** | 5.0 |
| *** # ND1_PRANKCD_TCSFMf_2AA_UB # *** | 5.0 |
| *** # ND1_MAFFTGI_NOISY_DNA_UB # *** | 4.0 |
| *** # ND1_TCOFFEETC_GBLOCKS_RYt_UB # *** | 3.0 |
| *** # ND1_TCOFFEE_GBLOCKS_DNA_UB # *** | 3.0 |
| *** # ND1_TCOFFEE_PSARALIGN_DEG_UB # *** | 3.0 |
| *** # ND1_FSANP_TRIMALG_CDN_UB # *** | 2.0 |
| *** # ND1_PRANKCDO_GBLOCKSC_2AA_UB # *** | 2.0 |
| *** # ND1_MAFFTLI_NOISY_DEG_UB # *** | 1.0 |
| *** # ND1_MAFFTLI_NOISY_RYt_UB # *** | 1.0 |13/72
### Chart
| Category | Total Topological Support |
|---|---|
| *** # ND1_MAFFTEI_DEG_UB # *** | 42.0 |
| *** # ND1_OPAL_DNA_UB # *** | 39.0 |
| *** # ND1_OPAL_RYt_UB # *** | 38.0 |
| *** # ND1_PRANKCDO_CDN_UB # *** | 3.0 |
| *** # ND1_PRANKCD_2AA_UB # *** | 0.0 |
1/5
CephaResults (File 3)
CephaResultsARC (File 4)

## Slide 8
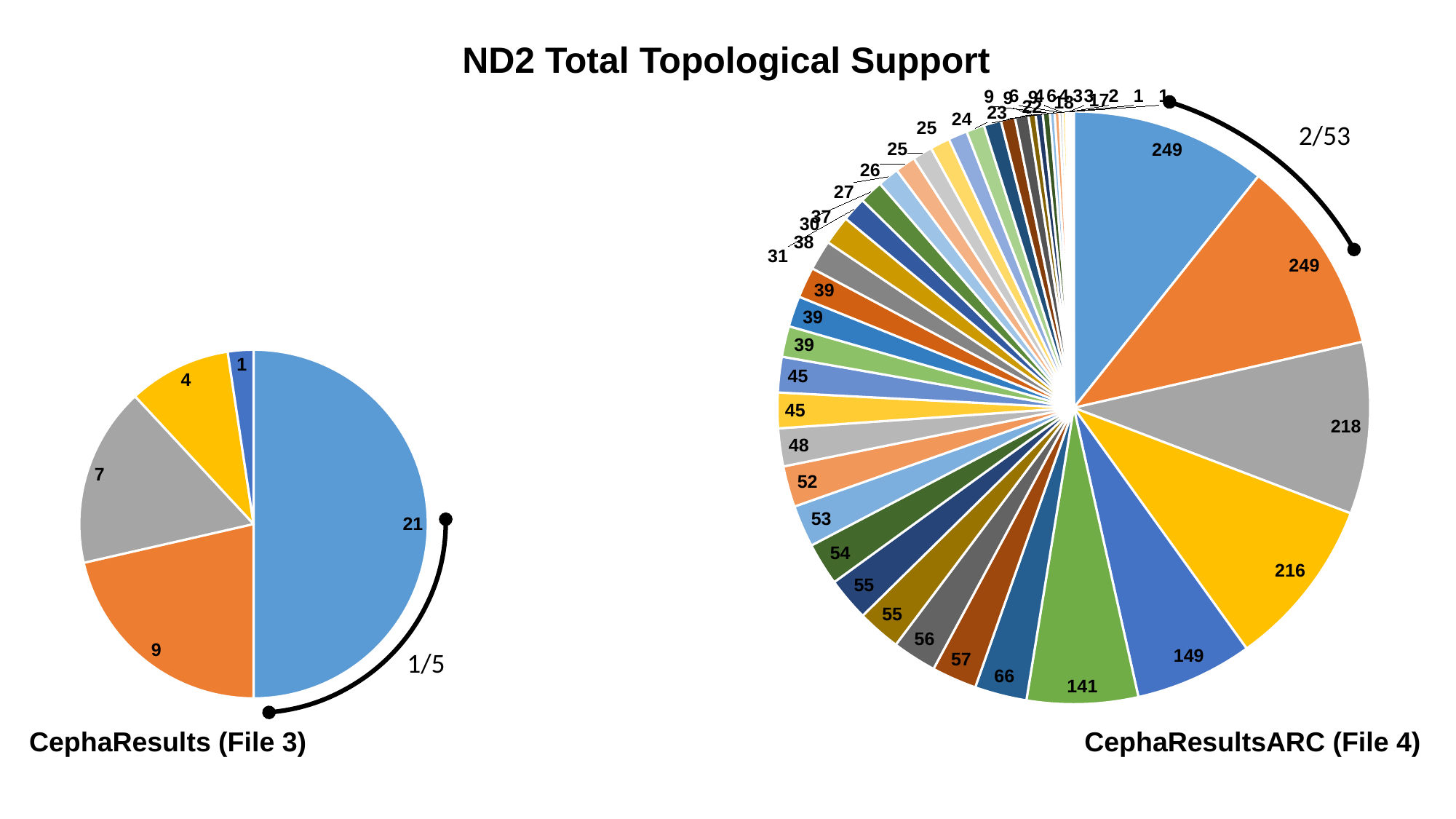

ND2 Total Topological Support
### Chart
| Category | Total Topological Support |
|---|---|
| *** # ND2_MAFFTA_TCSOGw_DNA_UB # *** | 249.0 |
| *** # ND2_MAFFTA_TCSf_DNA_UB # *** | 249.0 |
| *** # ND2_MAFFTLI_PSARALIGN_DNA_UB # *** | 218.0 |
| *** # ND2_MAFFTLI_TCSFMf_DNA_UB # *** | 216.0 |
| *** ND2_ALL_TRIMALC_DNA_UB *** | 149.0 |
| *** # ND2_TCOFFEETC_TRIMALP_DNA_UB # *** | 141.0 |
| *** # ND2_TCOFFEEPL_TCSFMw_DEG_UB # *** | 66.0 |
| *** # ND2_MAFFTGI_TRIMALA_DNA_UB # *** | 57.0 |
| *** # ND2_MAFFTA_TCSf_CDN_UB # *** | 56.0 |
| *** # ND2_MAFFTGI_TCSFMf_CDN_UB # *** | 55.0 |
| *** # ND2_MAFFTGI_TRIMALG_DNA_UB # *** | 55.0 |
| *** # ND2_MAFFTGI_TRIMALA_CDN_UB # *** | 54.0 |
| *** # ND2_MAFFTGI_TRIMAL_CDN_UB # *** | 53.0 |
| *** # ND2_MAFFTLI_PSARALIGN_CDN_UB # *** | 52.0 |
| *** # ND2_MAFFTLI_TRIMAL_DNA_UB # *** | 48.0 |
| *** ND2_ALL_WEAVEALIGN_DNA_UB *** | 45.0 |
| *** ND2_ALL_WEAVEALIGN_RYt_UB *** | 45.0 |
| *** # ND2_MACSE_TCSFMf_RYt_UB # *** | 39.0 |
| *** # ND2_MACSE_TRIMAL_RYt_UB # *** | 39.0 |
| *** # ND2_TCOFFEEPL_TCSw_DEG_UB # *** | 39.0 |
| *** # ND2_TCOFFEEPL_TCSw_DNA_UB # *** | 38.0 |
| *** # ND2_TCOFFEEPL_TCSw_RYt_UB # *** | 37.0 |
| *** # ND2_TCOFFEETC_TCSFMw_DNA_UB # *** | 31.0 |
| *** # ND2_TCOFFEE_TRIMALP_RYt_UB # *** | 30.0 |
| *** # ND2_MAFFTEI_PSARALIGN_2AA_UB # *** | 27.0 |
| *** # ND2_MAFFTGI_TCSFMf_2AA_UB # *** | 26.0 |
| *** # ND2_MACSE_TCSFMf_DEG_UB # *** | 25.0 |
| *** # ND2_MAFFTGI_TCSf_2AA_UB # *** | 25.0 |
| *** # ND2_MAFFTGI_TRIMALA_2AA_UB # *** | 24.0 |
| *** # ND2_MAFFTGI_TRIMALG_2AA_UB # *** | 23.0 |
| *** # ND2_MAFFTGI_TRIMAL_2AA_UB # *** | 22.0 |
| *** # ND2_TCOFFEEPL_TCSFMw_RYt_UB # *** | 18.0 |
| *** # ND2_TCOFFEE_GBLOCKS_DNA_UB # *** | 17.0 |
| *** # ND2_MAFFTF1_NOISY_DEG_UB # *** | 9.0 |
| *** # ND2_MAFFTF1_NOISY_DNA_UB # *** | 9.0 |
| *** # ND2_MAFFTF1_NOISY_RYt_UB # *** | 9.0 |
| *** # ND2_MAFFTA_TRIMALG_DEG_UB # *** | 6.0 |
| *** # ND2_MAFFTLI_TRIMALA_DEG_UB # *** | 6.0 |
| *** # ND2_FSA_TRIMALS_RYt_UB # *** | 4.0 |
| *** # ND2_MAFFTLI_GBLOCKSC_CDN_UB # *** | 4.0 |
| *** # ND2_CLUSTALW_GBLOCKSC_DNA_UB # *** | 3.0 |
| *** # ND2_MAFFTF2_GBLOCKS_DEG_UB # *** | 3.0 |
| *** # ND2_TCOFFEEPL_TRIMALP_DEG_UB # *** | 2.0 |
| *** # ND2_FSANP_GBLOCKS_RYt_UB # *** | 1.0 |
| *** # ND2_MAFFTGI_TRIMALG_CDN_UB # *** | 1.0 |
| # ND2_CLUSTALO_GBLOCKSC_DEG_UB # | 0.0 |
| # ND2_CLUSTALO_GBLOCKSC_RYt_UB # | 0.0 |
| # ND2_FSA_TRIMALS_DEG_UB # | 0.0 |
| # ND2_FSA_TRIMALS_DNA_UB # | 0.0 |
| # ND2_MACSE_TCSOGw_RYt_UB # | 0.0 |
| # ND2_MAFFTGI_GBLOCKSC_2AA_UB # | 0.0 |
| # ND2_MAFFTGI_TCSOGw_DEG_UB # | 0.0 |
| *** # ND2_TCOFFEE_TRIMALP_DEG_UB # *** | 0.0 |
2/53
### Chart
| Category | Total Topological Support |
|---|---|
| *** # ND2_MAFFTA_DNA_UB # *** | 21.0 |
| *** # ND2_MAFFTGI_CDN_UB # *** | 9.0 |
| *** # ND2_MACSE_RYt_UB # *** | 7.0 |
| *** # ND2_MAFFTGI_2AA_UB # *** | 4.0 |
| *** # ND2_MACSE_DEG_UB # *** | 1.0 |
1/5
CephaResults (File 3)
CephaResultsARC (File 4)

## Slide 9
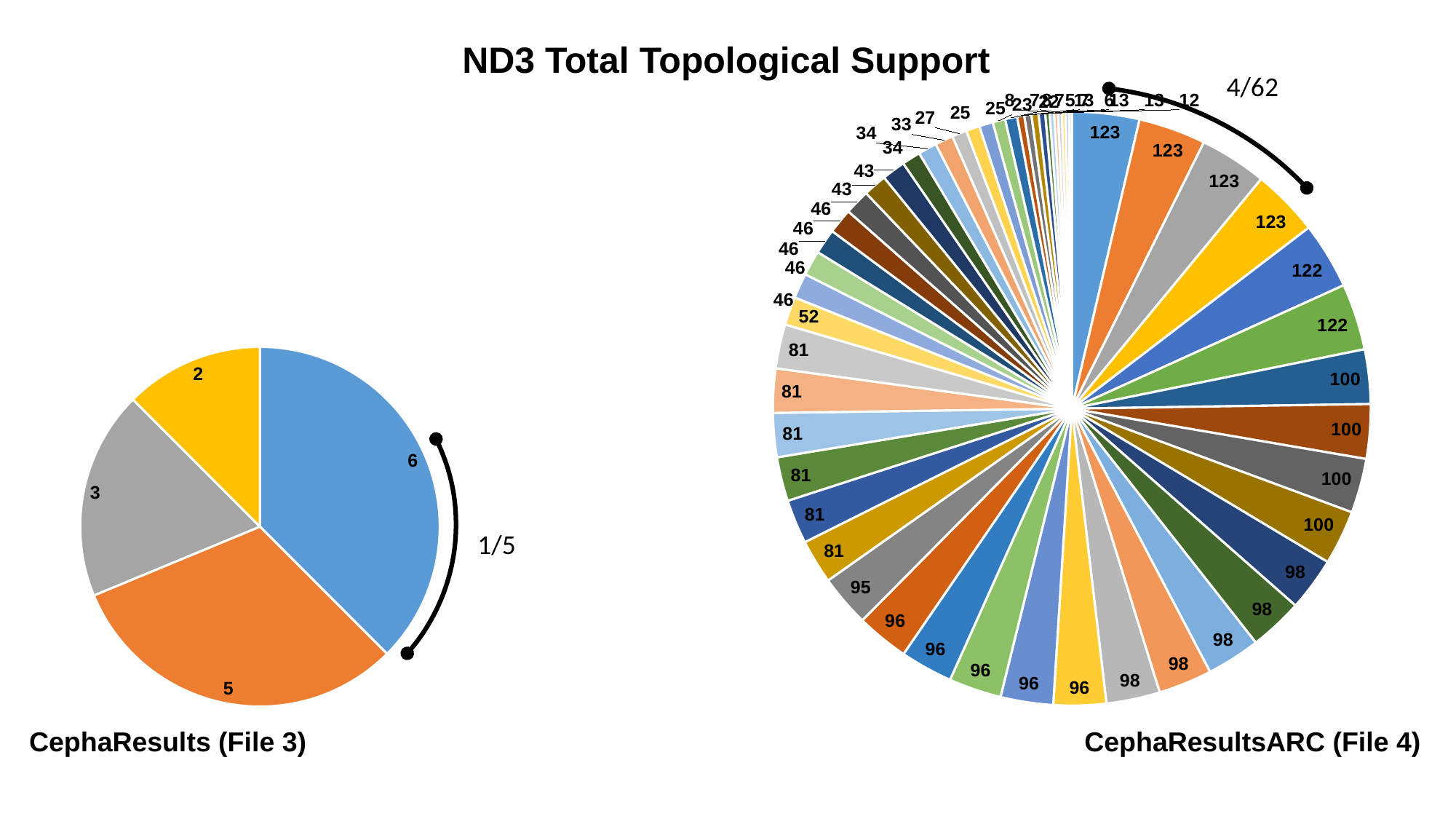

ND3 Total Topological Support
4/62
### Chart
| Category | Total Topological Support |
|---|---|
| *** # ND3_CLUSTALO_GBLOCKSC_DEG_UB # *** | 123.0 |
| *** # ND3_FSANP_MAXALIGN_DEG_UB # *** | 123.0 |
| *** # ND3_FSANP_TCSFMf_DEG_UB # *** | 123.0 |
| *** # ND3_FSANP_TRIMAL_DEG_UB # *** | 123.0 |
| *** ND3_ALL_MERGEALIGN_DEG_UB *** | 122.0 |
| *** ND3_ALL_WEAVEALIGN_DEG_UB *** | 122.0 |
| *** # ND3_PROBCONS_MAXALIGN_CDN_UB # *** | 100.0 |
| *** # ND3_PROBCONS_PSARALIGN_CDN_UB # *** | 100.0 |
| *** # ND3_PROBCONS_TCSFMf_CDN_UB # *** | 100.0 |
| *** # ND3_PROBCONS_TRIMAL_CDN_UB # *** | 100.0 |
| *** # ND3_CLUSTALW_GBLOCKS_DNA_UB # *** | 98.0 |
| *** # ND3_MAFFTGI_TCSf_DNA_UB # *** | 98.0 |
| *** # ND3_OPAL_TCSFMf_DNA_UB # *** | 98.0 |
| *** # ND3_PROBCONS_PSARALIGN_DNA_UB # *** | 98.0 |
| *** # ND3_PROBCONS_TRIMAL_DNA_UB # *** | 98.0 |
| *** # ND3_CLUSTALO_TCSFMw_DEG_UB # *** | 96.0 |
| *** # ND3_GRAMALIGN_TCSFMw_RYt_UB # *** | 96.0 |
| *** # ND3_GRAMALIGN_TCSOGw_DNA_UB # *** | 96.0 |
| *** # ND3_MAFFTFI_TCSFMw_DNA_UB # *** | 96.0 |
| *** # ND3_OPAL_MAXALIGN_DNA_UB # *** | 96.0 |
| *** ND3_ALL_WEAVEALIGN_DNA_UB *** | 95.0 |
| *** # ND3_FSA_TRIMALA_2AA_UB # *** | 81.0 |
| *** # ND3_MAFFTGI_MAXALIGN_2AA_UB # *** | 81.0 |
| *** # ND3_OPAL_PSARALIGN_2AA_UB # *** | 81.0 |
| *** # ND3_OPAL_TCSFMf_2AA_UB # *** | 81.0 |
| *** # ND3_OPAL_TRIMAL_2AA_UB # *** | 81.0 |
| *** # ND3_PROBCONS_TCSf_2AA_UB # *** | 81.0 |
| *** ND3_ALL_WEAVEALIGN_2AA_UB *** | 52.0 |
| *** # ND3_CLUSTALW_TRIMAL_RYt_UB # *** | 46.0 |
| *** # ND3_FSANP_TCSFMf_RYt_UB # *** | 46.0 |
| *** # ND3_FSANP_TCSf_RYt_UB # *** | 46.0 |
| *** # ND3_MACSE_GBLOCKSC_RYt_UB # *** | 46.0 |
| *** # ND3_MACSE_GBLOCKS_RYt_UB # *** | 46.0 |
| *** # ND3_PRANKCD_TRIMALA_CDN_UB # *** | 43.0 |
| *** # ND3_PRANKCD_TRIMALG_CDN_UB # *** | 43.0 |
| *** # ND3_CLUSTALO_GBLOCKSC_2AA_UB # *** | 34.0 |
| *** # ND3_PRANKCD_TRIMALG_2AA_UB # *** | 34.0 |
| *** # ND3_FSANP_MAXALIGN_RYt_UB # *** | 33.0 |
| *** # ND3_CLUSTALO_TCSw_DEG_UB # *** | 27.0 |
| *** # ND3_MAFFTFI_TRIMALS_DNA_UB # *** | 25.0 |
| *** # ND3_MAFFTLI_TRIMALP_DNA_UB # *** | 25.0 |
| *** # ND3_MACSE_PSARALIGN_RYt_UB # *** | 23.0 |
| *** # ND3_CLUSTALW_TCSw_DNA_UB # *** | 22.0 |
| *** # ND3_CLUSTALW_TCSw_RYt_UB # *** | 13.0 |
| *** # ND3_MAFFTEI_TRIMALS_DEG_UB # *** | 13.0 |
| *** ND3_ALL_WEAVEALIGN_CDN_UB *** | 13.0 |
| *** # ND3_PROBCONS_TCSOGw_DEG_UB # *** | 12.0 |
| *** # ND3_MAFFTEI_GBLOCKSC_CDN_UB # *** | 8.0 |
| *** # ND3_MAFFTGI_TCSf_CDN_UB # *** | 8.0 |
| *** # ND3_CLUSTALW_TRIMALS_RYt_UB # *** | 7.0 |
| *** # ND3_PRANKCD_TRIMALA_DEG_UB # *** | 7.0 |
| *** # ND3_PRANKCD_TRIMALG_DEG_UB # *** | 7.0 |
| *** # ND3_CLUSTALW_TRIMALP_DEG_UB # *** | 6.0 |
| *** # ND3_CLUSTALW_PSARALIGN_DEG_UB # *** | 5.0 |
| # ND3_GRAMALIGN_TCSOGw_RYt_UB # | 0.0 |
| # ND3_PRANKCD_TRIMALA_DNA_UB # | 0.0 |
| # ND3_PRANKCD_TRIMALG_DNA_UB # | 0.0 |
| # ND3_PRANKF_NOISY_DEG_UB # | 0.0 |
| # ND3_PRANKF_NOISY_DNA_UB # | 0.0 |
| # ND3_PRANKF_NOISY_RYt_UB # | 0.0 |
| # ND3_PRANKF_TRIMALA_RYt_UB # | 0.0 |
| # ND3_PRANKF_TRIMALG_RYt_UB # | 0.0 |
### Chart
| Category | Total Topological Support |
|---|---|
| *** # ND3_OPAL_DNA_UB # *** | 6.0 |
| *** # ND3_PROBCONS_2AA_UB # *** | 5.0 |
| *** # ND3_CLUSTALW_RYt_UB # *** | 3.0 |
| *** # ND3_MACSE_DEG_UB # *** | 2.0 |
| # ND3_MAFFTGI_CDN_UB # | 0.0 |
1/5
CephaResults (File 3)
CephaResultsARC (File 4)

## Slide 10
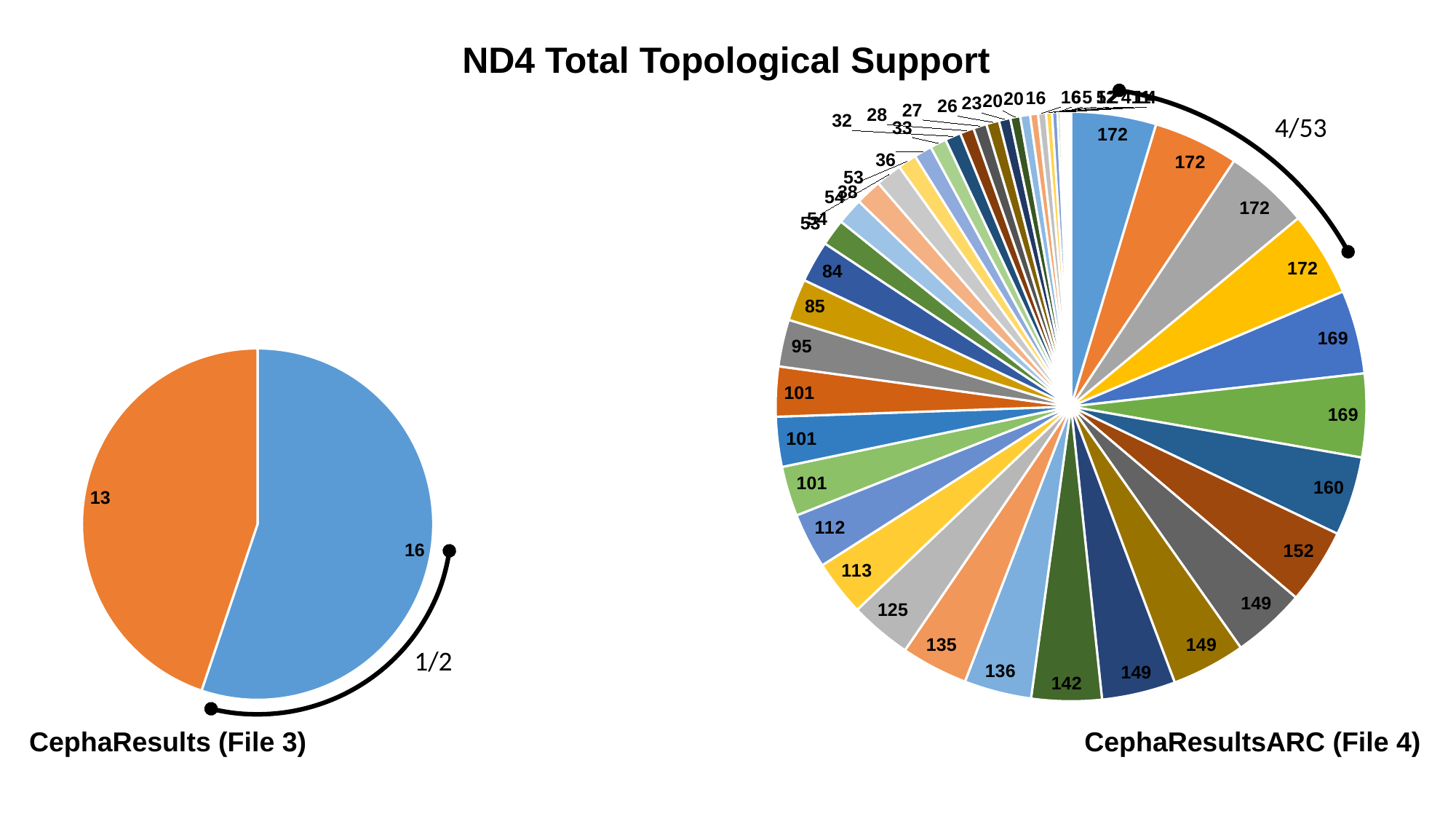

ND4 Total Topological Support
### Chart
| Category | Total Topological Support |
|---|---|
| *** # ND4_CLUSTALO_MAXALIGN_RYt_UB # *** | 172.0 |
| *** # ND4_CLUSTALO_TCSFMf_RYt_UB # *** | 172.0 |
| *** # ND4_CLUSTALO_TCSOGw_RYt_UB # *** | 172.0 |
| *** # ND4_MAFFTA_MAXALIGN_DNA_UB # *** | 172.0 |
| *** # ND4_MAFFTGI_TCSFMf_DNA_UB # *** | 169.0 |
| *** ND4_ALL_TRIMALC_RYt_UB *** | 169.0 |
| *** # ND4_MAFFTGI_TRIMAL_DNA_UB # *** | 160.0 |
| *** # ND4_MAFFTLI_TCSOGw_DNA_UB # *** | 152.0 |
| *** # ND4_MAFFTA_MAXALIGN_DEG_UB # *** | 149.0 |
| *** # ND4_MAFFTA_TCSFMf_DEG_UB # *** | 149.0 |
| *** # ND4_MAFFTA_TRIMAL_DEG_UB # *** | 149.0 |
| *** # ND4_MAFFTLI_MAXALIGN_DEG_UB # *** | 142.0 |
| *** # ND4_MAFFTLI_TRIMAL_DEG_UB # *** | 136.0 |
| *** # ND4_PROBCONS_TCSFMw_RYt_UB # *** | 135.0 |
| *** # ND4_TCOFFEE_PSARALIGN_DNA_UB # *** | 125.0 |
| *** # ND4_MAFFT_GBLOCKS_DNA_UB # *** | 113.0 |
| *** # ND4_MAFFT_GBLOCKS_RYt_UB # *** | 112.0 |
| *** ND4_ALL_MERGEALIGN_DEG_UB *** | 101.0 |
| *** ND4_ALL_TRIMALC_DEG_UB *** | 101.0 |
| *** ND4_ALL_WEAVEALIGN_DEG_UB *** | 101.0 |
| *** # ND4_OPAL_TCSFMw_DNA_UB # *** | 95.0 |
| *** # ND4_PRANK_TRIMALA_RYt_UB # *** | 85.0 |
| *** # ND4_PRANK_TRIMALG_RYt_UB # *** | 84.0 |
| *** # ND4_MACSE_TRIMALA_CDN_UB # *** | 54.0 |
| *** # ND4_MACSE_TRIMALG_CDN_UB # *** | 54.0 |
| *** # ND4_MACSE_TRIMALA_2AA_UB # *** | 53.0 |
| *** # ND4_MACSE_TRIMALG_2AA_UB # *** | 53.0 |
| *** ND4_ALL_TRIMALC_CDN_UB *** | 38.0 |
| *** # ND4_PRANKCD_GBLOCKSC_CDN_UB # *** | 36.0 |
| *** # ND4_TCOFFEETC_TCSw_DEG_UB # *** | 33.0 |
| *** # ND4_TCOFFEETC_TCSw_RYt_UB # *** | 32.0 |
| *** # ND4_TCOFFEE_PSARALIGN_RYt_UB # *** | 28.0 |
| *** # ND4_PRANK_TRIMALA_DNA_UB # *** | 27.0 |
| *** # ND4_PRANK_TRIMALG_DNA_UB # *** | 26.0 |
| *** # ND4_MAFFTLI_TCSFMf_2AA_UB # *** | 23.0 |
| *** # ND4_PRANKCDF_TCSFMw_DEG_UB # *** | 20.0 |
| *** # ND4_TCOFFEE_TCSw_DNA_UB # *** | 20.0 |
| *** # ND4_PRANKCDF_GBLOCKSC_2AA_UB # *** | 16.0 |
| *** # ND4_TCOFFEEPL_GBLOCKS_DEG_UB # *** | 16.0 |
| *** # ND4_PRANK_TRIMALA_DEG_UB # *** | 12.0 |
| *** # ND4_PRANK_TRIMALG_DEG_UB # *** | 11.0 |
| *** ND4_ALL_TRIMALC_2AA_UB *** | 6.0 |
| *** # ND4_CLUSTALO_TCSOGw_DEG_UB # *** | 5.0 |
| *** # ND4_TCOFFEE_TRIMALS_DNA_UB # *** | 5.0 |
| *** # ND4_MAFFTGI_NOISY_RYt_UB # *** | 4.0 |
| *** # ND4_TCOFFEEPL_PSARALIGN_DEG_UB # *** | 4.0 |
| *** # ND4_PRANKCDF_TRIMALP_DNA_UB # *** | 2.0 |
| *** # ND4_PRANKCDO_TRIMALP_DEG_UB # *** | 1.0 |
| # ND4_GRAMALIGN_NOISY_DEG_UB # | 0.0 |
| # ND4_MUSCLE_NOISY_DNA_UB # | 0.0 |
| # ND4_MUSCLE_TRIMALS_DEG_UB # | 0.0 |
| # ND4_MUSCLE_TRIMALS_RYt_UB # | 0.0 |
| # ND4_PRANKCD_TRIMALP_RYt_UB # | 0.0 |
4/53
### Chart
| Category | Total Topological Support |
|---|---|
| *** # ND4_MAFFTLI_DEG_UB # *** | 16.0 |
| *** # ND4_CLUSTALO_RYt_UB # *** | 13.0 |
1/2
CephaResults (File 3)
CephaResultsARC (File 4)

## Slide 11
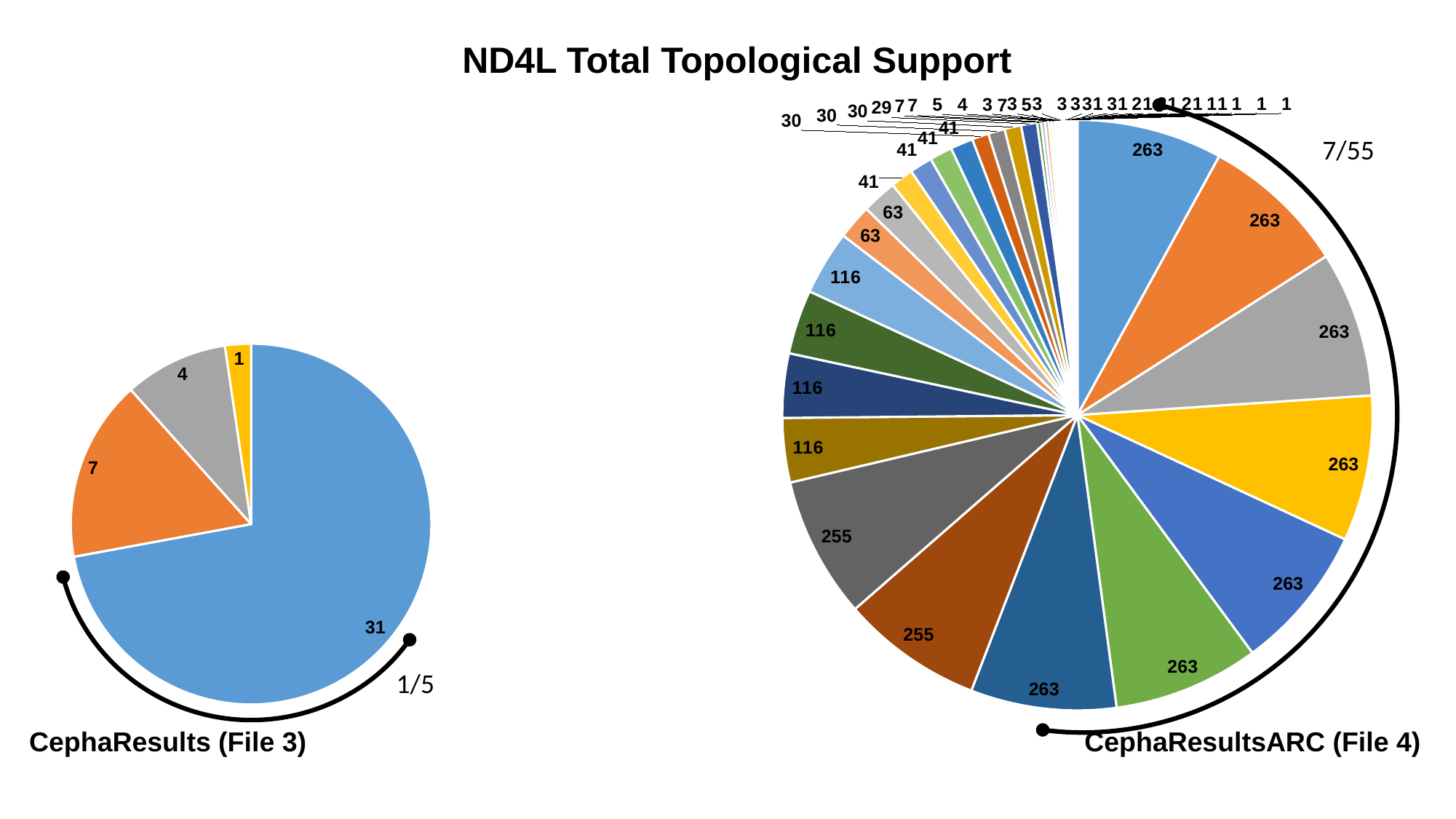

ND4L Total Topological Support
### Chart
| Category | Total Topological Support |
|---|---|
| *** # ND4L_CLUSTALW_PSARALIGN_DNA_UB # *** | 263.0 |
| *** # ND4L_CLUSTALW_TCSFMf_DNA_UB # *** | 263.0 |
| *** # ND4L_CLUSTALW_TCSf_DNA_UB # *** | 263.0 |
| *** # ND4L_CLUSTALW_TRIMAL_DNA_UB # *** | 263.0 |
| *** # ND4L_TCOFFEEPL_TCSFMw_DNA_UB # *** | 263.0 |
| *** # ND4L_TCOFFEEPL_TCSFMw_RYt_UB # *** | 263.0 |
| *** # ND4L_TCOFFEE_TCSFMw_DEG_UB # *** | 263.0 |
| *** ND4L_ALL_TRIMALC_CDN_UB *** | 255.0 |
| *** ND4L_ALL_TRIMALC_DNA_UB *** | 255.0 |
| *** # ND4L_MAFFTA_TCSw_DNA_UB # *** | 116.0 |
| *** # ND4L_MAFFTEI_TCSw_DEG_UB # *** | 116.0 |
| *** # ND4L_MAFFTLI_GBLOCKSC_DNA_UB # *** | 116.0 |
| *** # ND4L_MAFFTLI_TCSw_RYt_UB # *** | 116.0 |
| *** # ND4L_CLUSTALW_TCSOGw_DNA_UB # *** | 63.0 |
| *** # ND4L_MAFFT_TRIMALG_DNA_UB # *** | 63.0 |
| *** # ND4L_GRAMALIGN_PSARALIGN_CDN_UB # *** | 41.0 |
| *** # ND4L_GRAMALIGN_TCSFMf_CDN_UB # *** | 41.0 |
| *** # ND4L_GRAMALIGN_TCSf_CDN_UB # *** | 41.0 |
| *** # ND4L_GRAMALIGN_TRIMAL_CDN_UB # *** | 41.0 |
| *** # ND4L_CLUSTALW_TCSOGw_DEG_UB # *** | 30.0 |
| *** # ND4L_KALIGN_TCSFMf_DEG_UB # *** | 30.0 |
| *** # ND4L_KALIGN_TRIMAL_DEG_UB # *** | 30.0 |
| *** ND4L_ALL_TRIMALC_DEG_UB *** | 29.0 |
| *** # ND4L_OPAL_PSARALIGN_RYt_UB # *** | 7.0 |
| *** # ND4L_OPAL_TCSFMf_RYt_UB # *** | 7.0 |
| *** # ND4L_OPAL_TCSf_RYt_UB # *** | 7.0 |
| *** # ND4L_CLUSTALW_PSARALIGN_DEG_UB # *** | 5.0 |
| *** # ND4L_MUSCLE_TRIMALA_DNA_UB # *** | 5.0 |
| *** # ND4L_PRANKCD_TRIMALP_DEG_UB # *** | 4.0 |
| *** # ND4L_GRAMALIGN_PSARALIGN_2AA_UB # *** | 3.0 |
| *** # ND4L_GRAMALIGN_TCSFMf_2AA_UB # *** | 3.0 |
| *** # ND4L_GRAMALIGN_TCSf_2AA_UB # *** | 3.0 |
| *** # ND4L_KALIGN_TRIMAL_RYt_UB # *** | 3.0 |
| *** # ND4L_PRANKCDF_TRIMALA_2AA_UB # *** | 3.0 |
| *** # ND4L_PRANKCD_TRIMALG_2AA_UB # *** | 3.0 |
| *** ND4L_ALL_TRIMALC_2AA_UB *** | 3.0 |
| *** # ND4L_PRANKCD_TRIMALG_CDN_UB # *** | 2.0 |
| *** # ND4L_PRANKCD_TRIMALP_DNA_UB # *** | 2.0 |
| *** # ND4L_PRANKCD_TRIMALS_DNA_UB # *** | 2.0 |
| *** # ND4L_FSA_NOISY_RYt_UB # *** | 1.0 |
| *** # ND4L_GRAMALIGN_TRIMAL_2AA_UB # *** | 1.0 |
| *** # ND4L_KALIGN_TCSf_DEG_UB # *** | 1.0 |
| *** # ND4L_MAFFTLI_GBLOCKS_DEG_UB # *** | 1.0 |
| *** # ND4L_MAFFT_NOISY_DEG_UB # *** | 1.0 |
| *** # ND4L_MUSCLE_TRIMALA_DEG_UB # *** | 1.0 |
| *** # ND4L_MUSCLE_TRIMALA_RYt_UB # *** | 1.0 |
| *** # ND4L_MUSCLE_TRIMALG_DEG_UB # *** | 1.0 |
| *** # ND4L_MUSCLE_TRIMALG_RYt_UB # *** | 1.0 |
| *** # ND4L_PRANKCD_GBLOCKSC_2AA_UB # *** | 1.0 |
| # ND4L_FSA_NOISY_DNA_UB # | 0.0 |
| # ND4L_GRAMALIGN_TRIMALP_RYt_UB # | 0.0 |
| # ND4L_MAFFTGI_TRIMALS_RYt_UB # | 0.0 |
| # ND4L_MAFFTLI_TRIMALS_DEG_UB # | 0.0 |
| # ND4L_PRANKCDF_GBLOCKSC_CDN_UB # | 0.0 |
| # ND4L_PRANKCD_TRIMALA_CDN_UB # | 0.0 |
7/55
### Chart
| Category | Total Topological Support |
|---|---|
| *** # ND4L_CLUSTALW_DNA_UB # *** | 31.0 |
| *** # ND4L_GRAMALIGN_CDN_UB # *** | 7.0 |
| *** # ND4L_KALIGN_DEG_UB # *** | 4.0 |
| *** # ND4L_OPAL_RYt_UB # *** | 1.0 |
| # ND4L_GRAMALIGN_2AA_UB # | 0.0 |
1/5
CephaResults (File 3)
CephaResultsARC (File 4)

## Slide 12
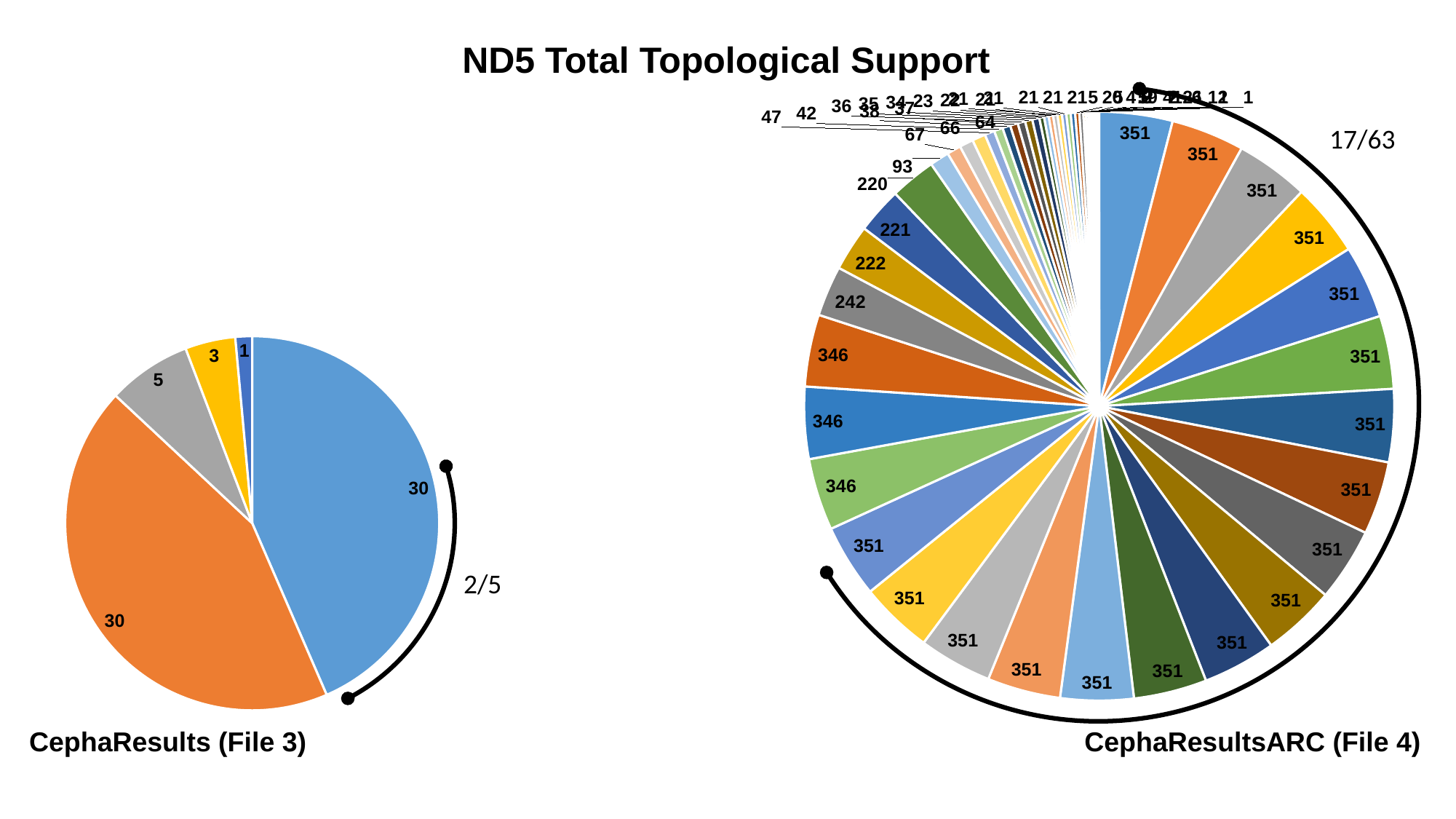

ND5 Total Topological Support
### Chart
| Category | Total Topological Support |
|---|---|
| *** # ND5_CLUSTALW_GBLOCKSC_RYt_UB # *** | 351.0 |
| *** # ND5_FSA_TRIMALA_DNA_UB # *** | 351.0 |
| *** # ND5_FSA_TRIMALA_RYt_UB # *** | 351.0 |
| *** # ND5_FSA_TRIMALG_DNA_UB # *** | 351.0 |
| *** # ND5_FSA_TRIMALG_RYt_UB # *** | 351.0 |
| *** # ND5_GRAMALIGN_MAXALIGN_RYt_UB # *** | 351.0 |
| *** # ND5_GRAMALIGN_PSARALIGN_RYt_UB # *** | 351.0 |
| *** # ND5_GRAMALIGN_TCSFMf_RYt_UB # *** | 351.0 |
| *** # ND5_GRAMALIGN_TCSf_RYt_UB # *** | 351.0 |
| *** # ND5_GRAMALIGN_TRIMAL_RYt_UB # *** | 351.0 |
| *** # ND5_MACSE_GBLOCKS_RYt_UB # *** | 351.0 |
| *** # ND5_MACSE_MAXALIGN_DNA_UB # *** | 351.0 |
| *** # ND5_MACSE_PSARALIGN_DNA_UB # *** | 351.0 |
| *** # ND5_MACSE_TCSFMf_DNA_UB # *** | 351.0 |
| *** # ND5_MACSE_TCSf_DNA_UB # *** | 351.0 |
| *** # ND5_MACSE_TRIMAL_DNA_UB # *** | 351.0 |
| *** # ND5_MAFFTGI_GBLOCKS_DNA_UB # *** | 351.0 |
| *** ND5_ALL_TRIMALC_DNA_UB *** | 346.0 |
| *** ND5_ALL_TRIMALC_RYt_UB *** | 346.0 |
| *** ND5_ALL_WEAVEALIGN_DNA_UB *** | 346.0 |
| *** # ND5_TCOFFEEPL_TCSOGw_RYt_UB # *** | 242.0 |
| *** # ND5_TCOFFEE_TCSFMw_DEG_UB # *** | 222.0 |
| *** # ND5_TCOFFEE_TCSFMw_DNA_UB # *** | 221.0 |
| *** # ND5_TCOFFEE_TCSFMw_RYt_UB # *** | 220.0 |
| *** # ND5_MAFFTGI_GBLOCKSC_DNA_UB # *** | 93.0 |
| *** # ND5_TCOFFEEPL_TCSw_DEG_UB # *** | 67.0 |
| *** # ND5_TCOFFEEPL_TCSw_DNA_UB # *** | 66.0 |
| *** # ND5_TCOFFEEPL_TCSOGw_DNA_UB # *** | 64.0 |
| *** # ND5_TCOFFEEPL_TCSw_RYt_UB # *** | 47.0 |
| *** # ND5_TCOFFEE_GBLOCKS_DEG_UB # *** | 42.0 |
| *** # ND5_OPAL_MAXALIGN_CDN_UB # *** | 38.0 |
| *** # ND5_OPAL_PSARALIGN_CDN_UB # *** | 37.0 |
| *** # ND5_OPAL_TCSFMf_CDN_UB # *** | 36.0 |
| *** # ND5_OPAL_TCSf_CDN_UB # *** | 35.0 |
| *** # ND5_OPAL_TRIMAL_CDN_UB # *** | 34.0 |
| *** # ND5_OPAL_MAXALIGN_2AA_UB # *** | 23.0 |
| *** # ND5_OPAL_PSARALIGN_2AA_UB # *** | 22.0 |
| *** # ND5_MACSE_MAXALIGN_DEG_UB # *** | 21.0 |
| *** # ND5_MACSE_PSARALIGN_DEG_UB # *** | 21.0 |
| *** # ND5_MACSE_TCSFMf_DEG_UB # *** | 21.0 |
| *** # ND5_MACSE_TCSf_DEG_UB # *** | 21.0 |
| *** # ND5_MACSE_TRIMAL_DEG_UB # *** | 21.0 |
| *** # ND5_OPAL_TCSFMf_2AA_UB # *** | 21.0 |
| *** # ND5_OPAL_TCSf_2AA_UB # *** | 20.0 |
| *** # ND5_OPAL_TRIMAL_2AA_UB # *** | 19.0 |
| *** # ND5_FSA_TRIMALA_DEG_UB # *** | 12.0 |
| *** # ND5_FSA_TRIMALG_DEG_UB # *** | 12.0 |
| *** # ND5_MAFFTA_GBLOCKSC_2AA_UB # *** | 8.0 |
| *** ND5_ALL_WEAVEALIGN_DEG_UB *** | 7.0 |
| *** # ND5_GRAMALIGN_TRIMALS_DNA_UB # *** | 6.0 |
| *** # ND5_MAFFTGI_GBLOCKSC_CDN_UB # *** | 5.0 |
| *** ND5_ALL_TRIMALC_2AA_UB *** | 5.0 |
| *** ND5_ALL_TRIMALC_CDN_UB *** | 5.0 |
| *** # ND5_MAFFTGI_GBLOCKSC_DEG_UB # *** | 4.0 |
| *** # ND5_PROBCONS_TRIMALP_DNA_UB # *** | 4.0 |
| *** # ND5_MAFFTA_TRIMALS_DEG_UB # *** | 2.0 |
| *** # ND5_TCOFFEEPL_NOISY_DNA_UB # *** | 2.0 |
| *** # ND5_MAFFTF1_TRIMALA_2AA_UB # *** | 1.0 |
| *** # ND5_TCOFFEEPL_NOISY_DEG_UB # *** | 1.0 |
| *** # ND5_TCOFFEEPL_NOISY_RYt_UB # *** | 1.0 |
| # ND5_PROBCONS_TRIMALP_DEG_UB # | 0.0 |
| # ND5_PROBCONS_TRIMALP_RYt_UB # | 0.0 |
| *** # ND5_TCOFFEE_TRIMALS_RYt_UB # *** | 0.0 |
17/63
### Chart
| Category | Total Topological Support |
|---|---|
| *** # ND5_GRAMALIGN_RYt_UB # *** | 30.0 |
| *** # ND5_MACSE_DNA_UB # *** | 30.0 |
| *** # ND5_OPAL_CDN_UB # *** | 5.0 |
| *** # ND5_MACSE_DEG_UB # *** | 3.0 |
| *** # ND5_OPAL_2AA_UB # *** | 1.0 |
2/5
CephaResults (File 3)
CephaResultsARC (File 4)

## Slide 13
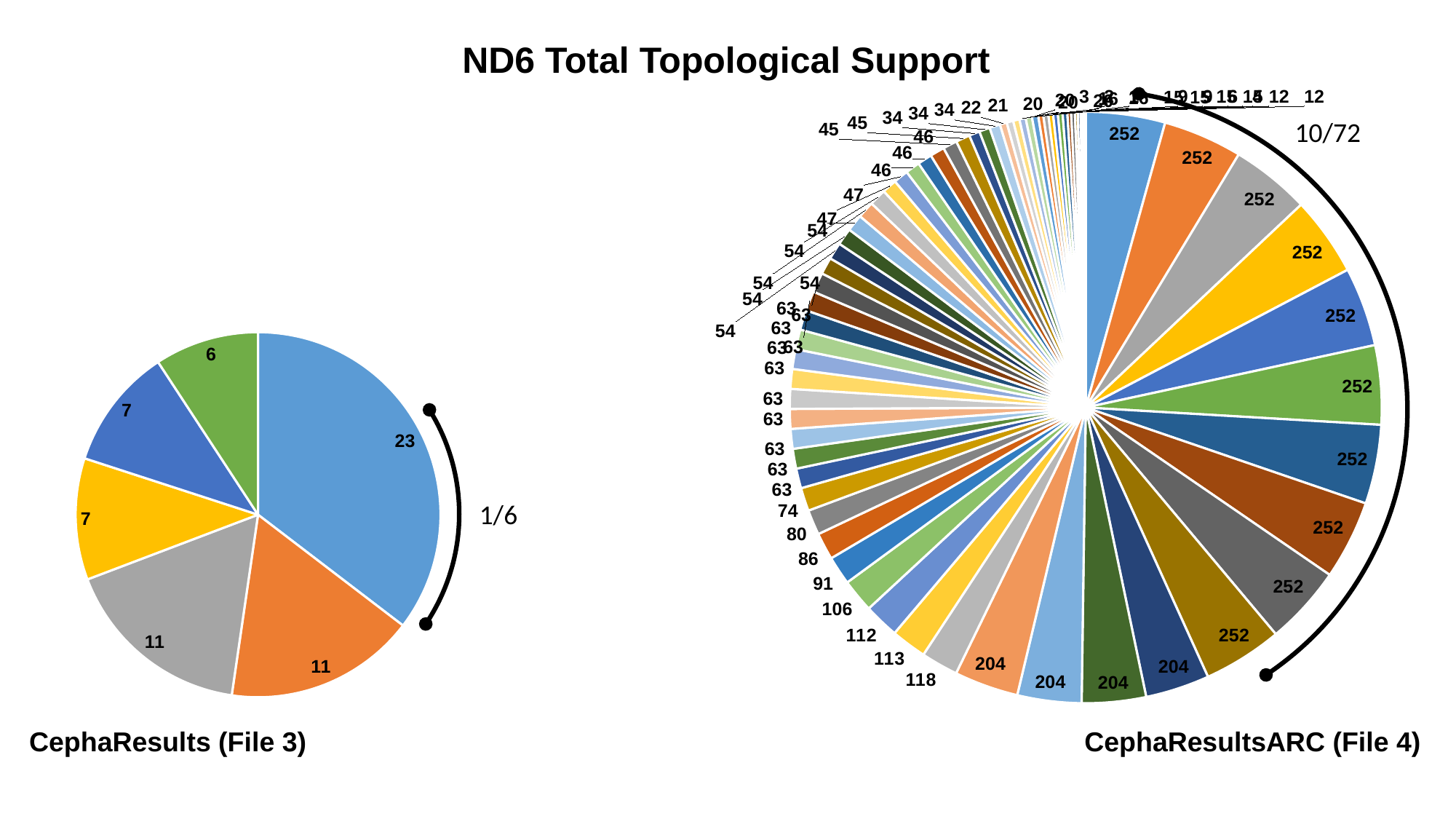

ND6 Total Topological Support
### Chart
| Category | Total Topological Support |
|---|---|
| *** # ND6_GRAMALIGN_MAXALIGN_DNA_UB # *** | 252.0 |
| *** # ND6_GRAMALIGN_TCSFMw_DNA_UB # *** | 252.0 |
| *** # ND6_MAFFTA_GBLOCKS_DNA_UB # *** | 252.0 |
| *** # ND6_MAFFTA_TCSw_DNA_UB # *** | 252.0 |
| *** # ND6_MAFFTGI_TCSFMf_DNA_UB # *** | 252.0 |
| *** # ND6_OPAL_GBLOCKSC_RYt_UB # *** | 252.0 |
| *** # ND6_OPAL_GBLOCKS_RYt_UB # *** | 252.0 |
| *** # ND6_OPAL_PSARALIGN_RYt_UB # *** | 252.0 |
| *** # ND6_OPAL_TCSFMf_RYt_UB # *** | 252.0 |
| *** # ND6_OPAL_TCSFMw_RYt_UB # *** | 252.0 |
| *** ND6_ALL_TRIMALC_DNA_UB *** | 204.0 |
| *** ND6_ALL_TRIMALC_RYt_UB *** | 204.0 |
| *** ND6_ALL_WEAVEALIGN_DNA_UB *** | 204.0 |
| *** ND6_ALL_WEAVEALIGN_RYt_UB *** | 204.0 |
| *** # ND6_TCOFFEEPL_PSARALIGN_DNA_UB # *** | 118.0 |
| *** # ND6_TCOFFEEPL_TCSOGw_DNA_UB # *** | 113.0 |
| *** # ND6_TCOFFEEPL_TCSf_DNA_UB # *** | 112.0 |
| *** # ND6_TCOFFEEPL_TRIMAL_DNA_UB # *** | 106.0 |
| *** # ND6_MAFFTA_TCSFMw_DEG_UB # *** | 91.0 |
| *** # ND6_TCOFFEE_PSARALIGN_DNA_UB # *** | 86.0 |
| *** # ND6_TCOFFEE_TCSf_DNA_UB # *** | 80.0 |
| *** # ND6_TCOFFEE_TRIMAL_DNA_UB # *** | 74.0 |
| *** # ND6_MACSE_TCSFMf_DEG_UB # *** | 63.0 |
| *** # ND6_MACSE_TCSf_DEG_UB # *** | 63.0 |
| *** # ND6_MAFFTA_TCSFMf_CDN_UB # *** | 63.0 |
| *** # ND6_MAFFTA_TRIMAL_CDN_UB # *** | 63.0 |
| *** # ND6_MAFFTA_TRIMAL_DEG_UB # *** | 63.0 |
| *** # ND6_MAFFTGI_GBLOCKSC_CDN_UB # *** | 63.0 |
| *** # ND6_MAFFTGI_MAXALIGN_CDN_UB # *** | 63.0 |
| *** # ND6_MAFFTLI_GBLOCKS_DEG_UB # *** | 63.0 |
| *** # ND6_OPAL_PSARALIGN_CDN_UB # *** | 63.0 |
| *** # ND6_OPAL_PSARALIGN_DEG_UB # *** | 63.0 |
| *** # ND6_OPAL_TCSf_CDN_UB # *** | 63.0 |
| *** # ND6_GRAMALIGN_PSARALIGN_2AA_UB # *** | 54.0 |
| *** # ND6_MAFFTA_TCSFMf_2AA_UB # *** | 54.0 |
| *** # ND6_MAFFTA_TRIMAL_2AA_UB # *** | 54.0 |
| *** # ND6_MAFFTGI_MAXALIGN_2AA_UB # *** | 54.0 |
| *** # ND6_MAFFTGI_TCSf_2AA_UB # *** | 54.0 |
| *** # ND6_OPAL_GBLOCKSC_2AA_UB # *** | 54.0 |
| *** ND6_ALL_TRIMALC_2AA_UB *** | 47.0 |
| *** ND6_ALL_WEAVEALIGN_2AA_UB *** | 47.0 |
| *** ND6_ALL_MERGEALIGN_DEG_UB *** | 46.0 |
| *** ND6_ALL_TRIMALC_DEG_UB *** | 46.0 |
| *** ND6_ALL_WEAVEALIGN_DEG_UB *** | 46.0 |
| *** ND6_ALL_TRIMALC_CDN_UB *** | 45.0 |
| *** ND6_ALL_WEAVEALIGN_CDN_UB *** | 45.0 |
| *** # ND6_MACSE_MAXALIGN_RYt_UB # *** | 34.0 |
| *** # ND6_MACSE_TCSf_RYt_UB # *** | 34.0 |
| *** # ND6_MACSE_TRIMAL_RYt_UB # *** | 34.0 |
| *** # ND6_PRANKF_TRIMALP_DNA_UB # *** | 22.0 |
| *** # ND6_PRANKF_TRIMALS_DNA_UB # *** | 21.0 |
| *** # ND6_GRAMALIGN_TRIMALP_RYt_UB # *** | 20.0 |
| *** # ND6_OPAL_TRIMALS_RYt_UB # *** | 20.0 |
| *** # ND6_PRANKCDF_TRIMALA_CDN_UB # *** | 20.0 |
| *** # ND6_PRANKCDF_TRIMALG_CDN_UB # *** | 20.0 |
| *** # ND6_PRANKCDF_TRIMALA_2AA_UB # *** | 16.0 |
| *** # ND6_PRANKCDF_TRIMALG_2AA_UB # *** | 16.0 |
| *** # ND6_PRANKCDF_TRIMALA_DNA_UB # *** | 15.0 |
| *** # ND6_PRANKCDF_TRIMALA_RYt_UB # *** | 15.0 |
| *** # ND6_PRANKCDF_TRIMALG_DNA_UB # *** | 15.0 |
| *** # ND6_PRANKCDF_TRIMALG_RYt_UB # *** | 15.0 |
| *** # ND6_GRAMALIGN_TCSw_DEG_UB # *** | 12.0 |
| *** # ND6_OPAL_TCSw_RYt_UB # *** | 12.0 |
| *** # ND6_MAFFTLI_TRIMALP_DEG_UB # *** | 9.0 |
| *** # ND6_OPAL_TRIMALS_DEG_UB # *** | 9.0 |
| *** # ND6_MAFFTGI_TCSOGw_DEG_UB # *** | 6.0 |
| *** # ND6_MACSE_MAXALIGN_DEG_UB # *** | 4.0 |
| *** # ND6_PRANKCDF_TRIMALA_DEG_UB # *** | 3.0 |
| *** # ND6_MACSE_TCSOGw_RYt_UB # *** | 2.0 |
| *** # ND6_TCOFFEE_GBLOCKSC_DNA_UB # *** | 1.0 |
| # ND6_PRANKCDF_TRIMALG_DEG_UB # | 0.0 |
| *** # ND6_PRANK_GBLOCKSC_DEG_UB # *** | 0.0 |
10/72
### Chart
| Category | Total Topological Support |
|---|---|
| *** # ND6_GRAMALIGN_RYt_UB # *** | 23.0 |
| *** # ND6_TCOFFEEPL_DNA_UB # *** | 11.0 |
| *** # ND6_TCOFFEE_DNA_UB # *** | 11.0 |
| *** # ND6_MACSE_DEG_UB # *** | 7.0 |
| *** # ND6_MAFFTA_CDN_UB # *** | 7.0 |
| *** # ND6_GRAMALIGN_2AA_UB # *** | 6.0 |
1/6
CephaResults (File 3)
CephaResultsARC (File 4)
